# Intramolecular exciton coupling modulates the convergent singlet-triplet energy gap toward NIR-emissive heavy-atom-free Oligo-BODIPY photosensitizers

**DOI:** 10.1039/d5sc08201c

**Published:** 2025-12-09

**Authors:** Chunyan Pan, Jinsong Shao, Zhengxin Kang, Fan Lv, Xiankang Zhang, Jiangang Gao, Xinsheng Xu, Yaxiong Wei, Erhong Hao, Lijuan Jiao

**Affiliations:** a Key Laboratory of Functional Molecular Solids of Ministry of Education, College of Chemistry and Materials Science, School of Physics and Electronic Information, Anhui Normal University Wuhu 241002 China kangzx@mail.ahpu.edu.cn Davidl@mail.ustc.edu.cn haoehong@ahnu.edu.cn jiao421@ahnu.edu.cn; b School of Chemical and Environmental Engineering, Anhui Polytechnic University Wuhu 241000 China

## Abstract

Heavy-atom-free triplet photosensitizers excited by long-wavelength light are essential for advancing applications in biomedicine and energy. However, the rational design of efficient heavy-atom-free triplet dyes becomes increasingly challenging as molecular engineering shifts absorption to the near-infrared (NIR) region, while still requiring retention of appreciable fluorescence. Herein, we introduce a novel strategy leveraging intramolecular exciton coupling to construct a series of covalently β,β-linked, coplanar BODIPY oligomers (dimer to tetramer), featuring multiple transition dipole moments in a head-to-tail chromophore arrangement. These architectures, lacking pyrrolic substituents, were regioselectively synthesized *via* a novel palladium(ii)-catalyzed dehydrogenative strategy in one-pot. They exhibit intense intramolecular J-type exciton coupling (3584 cm^−1^), not only enabling tunable absorption/emission (500–800 nm) and good fluorescence quantum yields (0.36–0.44), but also yielding significantly long-lived triplet excited states up to 143 µs, efficient reactive oxygen species (ROS) generation, and highly efficient single-photon absorption-based upconversion (SPA-UC). Theoretical calculations reveal that convergent singlet-triplet energy gaps (Δ*E*_S–T_, from 0.849 to 0.154 eV) are key factors for enhancing the spin–orbit coupling mediated intersystem crossing (SOC-ISC), driven by reduced singlet state energies without significant triplet state perturbation. This work establishes intramolecular exciton coupling in conjugated BODIPY oligomers as a versatile strategy to design heavy-atom-free photosensitizers with simultaneous NIR emission and high triplet yields, unlocking potential in biomedicine, photocatalysis, and beyond.

## Abstract 2

This work establishes intramolecular exciton coupling in conjugated β,β-linked BODIPY oligomers (up to tetramer) as a versatile strategy to design heavy-atom-free photosensitizers with simultaneous NIR emission and long-lived triplet states.

## Graphical abstract

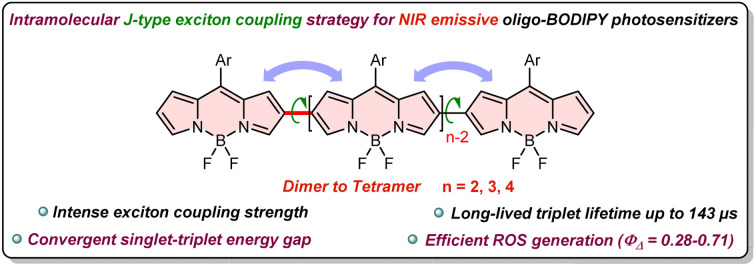

## Introduction

Heavy-atom-free triplet photosensitizers are pivotal to advancing photochemical technologies owing to their efficient intersystem crossing (ISC), long-lived triplet excited states, and versatile energy/electron transfer capabilities.^1^ These properties drive diverse applications ranging from photocatalysis^2,3^ and photovoltaics^4^ to photodynamic therapy^5–7^ and luminescent materials.^8,9^ A critical challenge lies in designing heavy-atom-free systems that combine strong near-infrared (NIR, *λ* > 700 nm) emission with efficient reactive oxygen species (ROS) generation under long-wavelength excitation.^10,11^ Such systems are highly desirable, as they avoid dark toxicity while leveraging prolonged triplet-state lifetimes for enhanced therapeutic and catalytic performance. Despite progress, achieving this balance remains elusive, necessitating innovative molecular design strategies.

Enhancing spin–orbit coupling (SOC) to promote ISC in organic dyes without heavy atoms requires precise molecular engineering.^12–15^ Strategies guided by El-Sayed's rule, such as introducing low-lying *n*π*–ππ* transitions,^16^ rigidifying/extending π-conjugation^17–22^ or constructing donor–acceptor (D–A) dyads^23–26^ to reduce singlet-triplet energy gaps (Δ*E*_S–T_), and suppressing exciton-vibrational coupling to enhance SOC by minimizing nonradiative decay,^27,28^ have shown promise. However, persistent challenges, including competitive excited-state relaxation pathways (*e.g.*, internal conversion, vibrational relaxation) and excessively small Δ*E*_S–T_ dictated by the energy gap law,^21^ limit SOC matrix elements below the threshold required for efficient ISC. Consequently, few heavy atom-free NIR triplet photosensitizers exist, highlighting the need for novel approaches to amplify SOC while maintaining desirable photophysical properties.

Boron dipyrromethene (BODIPY) dyes,^29–33^ have become one of the most pivotal fluorescent materials owing to their exceptional photophysical properties, tunable redox characteristics, and remarkable photochemical stability.^34–37^ However, their intrinsic spin-forbidden ISC results in negligible triplet-state population, limiting their utility as triplet photosensitizers.^38–46^ Recent efforts to address this have focused on extending π-conjugation through fusing heteroatomic or twisted rings,^12,47–54^ or appending specific electron-donor groups to the BODIPY core to activate charge recombination.^55–57^ While these modifications enhance ISC through SOC activation, they often compromise solubility, redshift absorption insufficiently, or quench fluorescence—critical drawbacks for applications that demand both imaging capability and therapeutic function. Thus, a key unmet challenge is to engineer BODIPY derivatives that simultaneously achieve NIR absorption, efficient ISC, and retained fluorescence through a balanced interplay of localized and charge-transfer excited states.

**Fig. 1:**
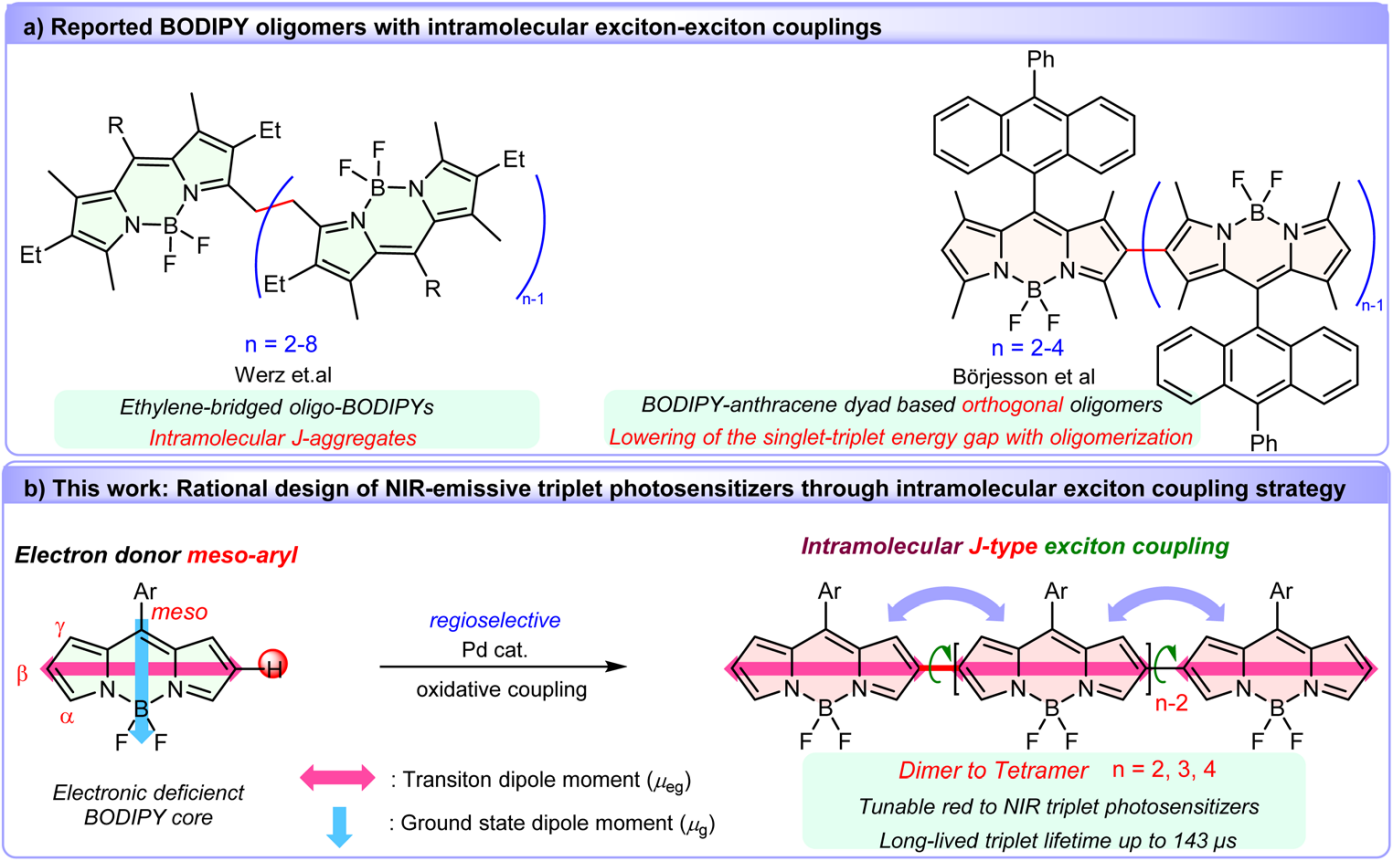
(a) Previously reported BODIPY oligomers with intramolecular exciton coupling. (b) Pd-catalyzed oxidative oligomerization of BODIPYs reported in this work, and schematic representation of the proposed concept for multiple intramolecular J-type exciton coupling in this covalently β,β-linked BODIPY oligomers.

Exciton coupling offers a compelling solution. By aligning transition dipole moments in J-aggregate configurations, this strategy has emerged as a viable approach to not only narrow the energy gap for allowing a red-shifted excitation wavelength *via* Coulombic coupling of localized transition dipole oscillators,^58–61^ but also enhance the radiative rate due to the increase of the transition dipole moments of the lowest excited state.^62^ As a proof concept, numerous covalently linked BODIPY dimers featuring favorable characteristics have been reported.^63–69^ In particular, a remarkable series of ethylene-bridged oligo-BODIPYs (Fig. 1a) exhibit intense J-type excitonic coupling, yielding red-shifted, narrowed absorption/emission bands and near-unity fluorescence quantum yields.^70–72^ On the other hand, exciton coupling systems of triplet state photosensitizers can selectively lower singlet-state energies without perturbing triplet or charge-separated states, thereby reducing Δ*E*_S–T_ to enhance SOC-driven ISC.^73–77^ Such resultant convergent in the singlet-triplet energy gap (Δ*E*_S–T_) are well conducive to enhancing SOC-ISC. Recent work by Börjesson *et al.* demonstrated this principle using BODIPY-anthracene dyad oligomers (Fig. 1a),^73^ achieving progressively reduced Δ*E*_S–T_. However, steric hindrance from 1,3,5,7-tetramethyl groups enforced orthogonal conformations, limiting red-shifted absorption. Notably, very recently, Fan, Du and coworkers developed cyanine dimer based NIR photosensitizers through elegantly tuning exciton coupling effects by connecting two cyanine molecules at the *N*-indole site with nonconjugated linkers.^77^

These insights motivated us to leverage precise control of intramolecular exciton coupling as a powerful molecular design strategy to develop NIR-emissive BODIPY-based photosensitizers. Herein, we present a class of covalently β,β-linked, coplanar BODIPY oligomers (up to tetramer) synthesized *via* a novel one-pot Pd(ii)-catalyzed oxidative C–H cross-coupling. This direct tethering strategy aligns the S_0_–S_1_ transition dipole moments of the BODIPY chromophores in a flexible head-to-tail connection (Fig. 1b), inducing intense intramolecular J-type exciton coupling. Crucially, oligomerization transforms the highly fluorescent BODIPY monomer into efficient triplet generators with long triplet lifetimes. The resulting oligomers feature stepwise red-shifted absorption (up to 710 nm) and reduced singlet-state energies (without significant triplet state perturbation) while retaining moderate fluorescence quantum yields (0.36–0.44)—a rare combination for NIR triplet photosensitizers. This work establishes intramolecular exciton coupling strategy in conjugated BODIPY oligomers for designing heavy atom-free NIR photosensitizers, merging synthetic accessibility with programmable photophysical properties for applications in biomedicine, photocatalysis, and beyond.

## Results and discussion

### Synthesis and characterization

**Scheme 1:**
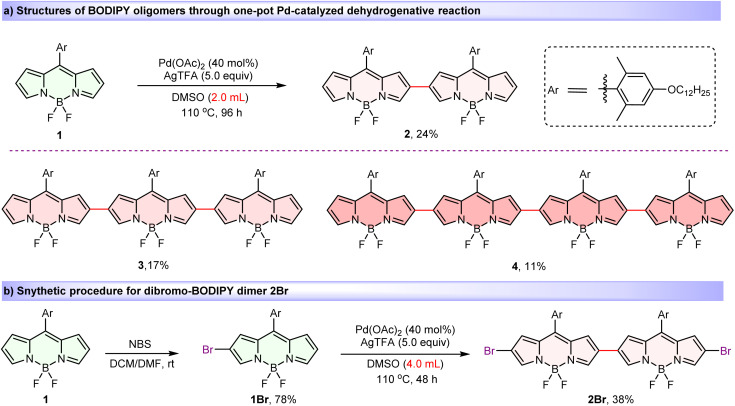
Synthesis and structures of BODIPY oligomers 2–4 and dimer 2Br*via* regioselective Pd-catalyzed dehydrogenative strategy.

Introducing substantial steric hindrance and lengthy substituents during the BODIPY oligomer synthesis effectively mitigates detrimental aggregation while concurrently promoting superior solubility.^78,79^ Thus, key BODIPY monomer 1, bearing long alkoxy chain on the bulky meso-aryl group was designed and prepared (see the ESI for synthetic details). Then, by modifying our previously reported regioselective Pd(ii)-catalyzed oxidative C–H cross-coupling reaction by specifically replacing reaction solvent from pivalic acid to dimethylsulfoxide (DMSO),^80^ we obtained a series of covalently β,β-linked BODIPY oligomers. When BODIPY 1 was reacted in DMSO at 110 °C in the presence of a catalytic amount of Pd(OAc)_2_ and 5.0 equiv. of AgTFA for 96 h (Scheme 1a), dimer 2 (24%), trimer 3 (17%) and tetramer 4 (11%) were isolated in one-pot. Moreover, mono-brominated precursor 1Br, obtained from the bromination reaction of monomer 1 and *N*-bromosuccinimide (NBS), also proceeded smoothly in this reaction, yielding the desired dibromo-dimer 2Br in 38% (Scheme 1b). Significantly, all oligomers exhibited excellent ambient stability and were unequivocally characterized by nuclear magnetic resonance spectroscopy (NMR) and HRMS (MALDI-TOF). The observed high β,β-regioselectivity is attributed to the high nucleophilicity of the β-position on the BODIPY core, which is analogous to the Pd(ii)-catalyzed oxidative dimerization of indoles to form 3,3′-biindoles.^81^

**Fig. 2:**
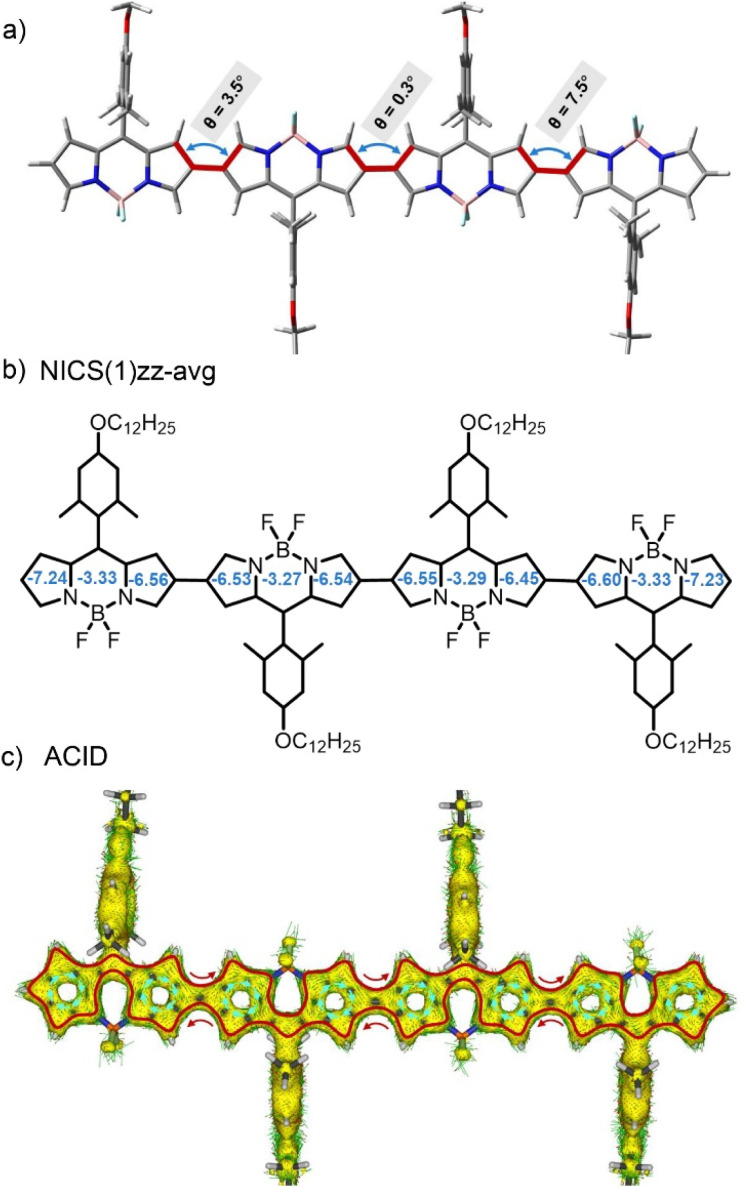
(a) Optimal ground-state conformation and dihedral angles of different BODIPY units. (b) Calculated NICS(1)zz-avg values and (c) ACID plot of tetrameric BODIPY 4.

Next, in order to gain a better understanding of the molecular structures, we first performed ground-state structural optimization of BODIPY oligomer molecules using density functional theory (DFT) calculations at the B3LYP/6-31G(d,p) level.^74,82^ As illustrated in Fig. 2a, taking the tetrameric BODIPY 4 as an example, the dihedral angles between BODIPY units in the oligomer approach 0° due to minimal steric hindrance between adjacent BODIPY moieties, resulting in a coplanar configuration. This planar geometry enhances π–π interactions and facilitates electronic coupling among BODIPY subunits through strengthened orbital overlap. Moreover, the orthogonal conformation between the planes defined by the meso-aryl substituent and each linked BODIPY cores, are crucial for inhibiting excessive π–π interaction in solid state.

Then, to further investigate the aromaticity patterns of these BODIPY oligomers, we performed nucleus-independent chemical shift (NICS) and anisotropy of current-induced density (ACID) analyses at the B3LYP/6-31G(d,p) level, using tetramer 4 as a representative case. For the 1,3-azaborine moieties, the NICS(1)zz-avg values ranged from −3.27 to −3.33 ppm (Fig. 2b), indicating weak aromatic character. In contrast, the adjacent pyrrole rings exhibited enhanced aromaticity, with NICS values of −6.45 to −7.24 ppm, although still lower than those of typical aromatic systems (*e.g.*, benzene: −15 to −20 ppm). ACID calculations (Fig. 2c) demonstrated that, despite the near-perfect coplanarity between the 1,3-azaborine and pyrrole rings, the electrons of the boron atom predominantly occupied p-orbitals with weak π-orbital interactions from nitrogen atoms, resulting in negligible contributions to ACID and thereby inhibiting diamagnetic ring current formation in the 1,3-azaborine region. This finding is consistent with the weak aromaticity indicated by the NICS(1)zz-avg calculations (−3.27 to −3.33 ppm). Conversely, the nitrogen lone pairs in pyrroles fully participated in the conjugation network *via* resonance effects (green arrows, Fig. 2c), sustaining both electron delocalization and diamagnetic current pathways essential for aromaticity. This marked difference highlights the regulatory role of heteroatom orbital characteristics in conjugation continuity. Notably, parallel local current vortices emerged along the upper and lower edges of β-carbon-connected C–C bonds between BODIPY units. The synergistic interaction of these local currents established a global macrocyclic current (clockwise direction, red lines, Fig. 2c), enabling multiple BODIPY units to form an integrated aromatic system through long-range conjugation. This global conjugation significantly enhanced optical absorption intensity and induced substantial bathochromic shifts in both absorption and emission spectra.

### Steady-state absorption

All target BODIPY dyes demonstrated good solubility in common organic solvents, including toluene, dichloromethane, chloroform, and tetrahydrofuran (THF) (Fig. S1–11). Photophysical data for the oligomers are summarized in Tables 1 and S1. Compared to BODIPY monomer (absorption/emission: 503/518 nm in toluene, Table 1), this β,β-linked BODIPY dimer (2) showed significant bathochromic shift in absorption exceeding 110 nm, indicating effective electron delocalization after direct dimerization as result of strong exciton coupling interactions within dual chromophores where the transition dipole moments are arranged in an inherent head-to-tail configuration with flexible single-bond connectivity. Moreover, dimer 2Br with electron-withdrawing group exhibited further red-shifted absorption of 634 nm.

**Table 1:** Photophysical Properties of BODIPY Oligomers and Monomer in Toluene

dyes	*λ* ^max^_abs_ [nm]	*ε* [M^−1^ cm^−1^]^a^	*λ*_em_ [nm]	Stokes shift [cm^−1^]	*f* b	*µ*_eg_ [D]^c^	*Φ* d	*Φ*_Δ_ e
1	503	64500	518	580	0.58	6.66	0.94	—f
2	616	65500	668	1260	1.33	10.63	0.39	0.28
2Br	634	88600	688	1240	1.64	12.01	0.27	0.71
3	670	118400	718	1000	2.45	14.86	0.44	0.36
4	710	176000	751	770	3.05	16.89	0.36	0.36

a Corresponding to the strongest absorption maximum.

b Oscillator strength according to eqn (S3).

c Transition dipole moment referring to the S_1_ excited state according the eqn (S4).

d Absolute fluorescence quantum yields.

e Relative reactive oxygen quantum yields in chloroform.

f means not tested.

**Fig. 3:**
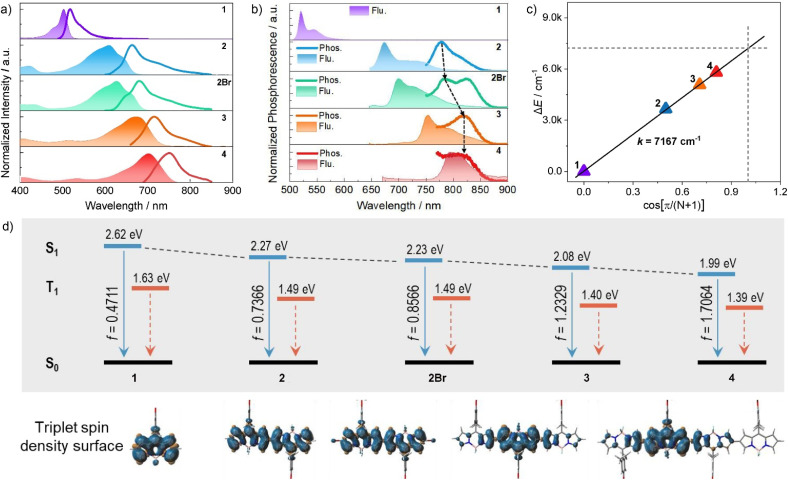
(a) Normalized absorption and fluorescence spectra of compounds 1–4 and 2Br in chloroform. (b) Phosphorescence (delay time of 0.2 s) and fluorescence emission spectra of oligo-BODIPYs at 77 K, *λ*_ex_ = 620 nm (464 nm for 1). (c) Plot of the exciton splitting energy as a function of the number of BODIPY units *N* in the β,β-linked BODIPY arrays. A linear correlation is observed for the energy difference between the low-energy bands of BODIPY oligomers. (d) Molecular energy levels of singlet and triplet excited states, and the triplet spin density surface.

As expected, the absorption energy converges with an increasing number of BODIPY units. The trimeric BODIPY 3 exhibited further red-shifted absorption peaking at 670 nm, along with broad spectral features in toluene (Fig. 3a and Table 1). Especially, the BODIPY tetramer 4 displayed intense absorption spanning from 600 to 800 nm, with a significantly red-shifted maximum at 710 nm and a large molar extinction coefficient (*ε* = 1.76 × 10^5^ M^−1^ cm^−1^), underscoring its exceptional light-harvesting properties as a NIR excitable dye. Notably, BODIPY dimers (2 and 2Br) and trimer (3) all displayed broad absorption bands across the visible to NIR spectral region with a full width at half-maximum (FWHM) exceeding 92 nm, whereas tetramer 4 showed a slightly decreased FWHM of 85 nm (Fig. 3a and SI). These results suggest that involvement of multiple transition dipole moments in BODIPY array with conformational flexibility, leads to the effective J-aggregate-like Davydov splitting^83,84^ superposition in solution.

### Exciton coupling analysis

To further understand the observed steady-state absorption features and splitting energy induced by intramolecular exciton coupling effects, given the strictly linear orientation of transition dipole moments in BODIPY oligomers 1–4, a simple point-dipole exciton coupled model of Kasha's theory was used to evaluate the intramolecular exciton coupling strength (V). This was calculated using the simplified eqn (1):^85,86^

1 Δ*E* = 2*V* cos[π/(*N* + 1)]

where Δ*E* is the red-shifted energy of the lowest excited state between BODIPY oligomers and the monomer derived from the corresponding experimental absorption spectrum in toluene, *N* is the number of BODIPY units. Remarkably, a positive linear fitting was observed between the energy difference (Δ*E*) and the numbers of BODIPY units, smoothly giving a calculated slope of 7167 cm^−1^, corresponding to an average coupling strength *V* = 3584 cm^−1^ (Fig. 3c). This exceptionally large value, which is far exceeding those reported for cyclic BODIPY systems,^87–90^ confirms robust intramolecular J-type exciton coupling arising from the head-to-tail alignment of transition dipole moments. The results highlight the critical role of covalent β,β-linkages in enabling intense excitonic interactions, which drive the pronounced red-shifts and splitting observed in these oligomers. Computational analyses reveal that as the oligomerization degree of BODIPY assemblies approaches infinity, the S_1_ excited-state energy level asymptotically converges to a limiting value of 1.58 eV (784 nm).

To quantify electronic coupling in BODIPY oligomers, we calculated the oscillator strength (*f*) and transition dipole moment (*µ*_eg_) within the exciton coupled state from respective molar absorptivity spectra using eqn (S3) and (S4) (SI).^79,91^ It was noted that both the calculated oscillator strengths (0.58–3.05, consistent with DFT calculation results, Fig. 3c) and transition dipole moments (6.66–16.89 D) exhibited a progressive increase with oligomer size (Table 1), which confirmed the existence of strong intramolecular J-type exciton coupling in BODIPY oligomers. Furthermore, the squared transition dipole moments and oscillator strength values scaled linearly with the number of BODIPY units (Fig. S19 and Table S3), suggesting that increasing the number of BODIPY units in oligomers connecting in a head-to-tail arrangement could statistically enhance electronic coupling and improve light-absorption capabilities compared to monomeric BODIPY, which is consistent with ACID calculations (Fig. 2c).

### Steady-state emission efficiency

Compared to the monomer, BODIPY dimer 2 exhibited lowered yet good fluorescence quantum yield of 0.39 in toluene, showing the red-shifted emission maximum at 668 nm along with large Stokes shifts of 1260 cm^−1^ (Fig. 3a and Table 1). Dimer 2Br displayed the stabilized fluorescence quantum yield of 0.27, despite of the involvement of heavy-atom Br. Remarkably, BODIPY trimer 3 manifested red-shifted emission with maxima in 718 nm, and achieved much higher fluorescence quantum yield (*Φ* = 0.44) compared to their dimer counterparts. Notably, the BODIPY tetramer 4 exhibited favorable NIR emission at 751 nm with a good fluorescence quantum yield of 0.36. Due to their high molar extinction coefficients and good fluorescence quantum yields, these trimer and tetramer exhibited high fluorescence brightness (52 100–63400 cm^−1^).

### Formation and energy of the triplet state

To investigate the triplet-state energetics of oligomeric BODIPY systems, we systematically analyzed their low-temperature (77 K) phosphorescence and fluorescence emission profiles. As shown in Fig. 3b, phosphorescence emission was undetectable for monomer 1 due to inefficient ISC, though theoretical calculations predicted a triplet energy (*T*_1_) of 1.63 eV. Dimers 2 and 2Br exhibited phosphorescent maxima at 778 nm (1.59 eV) and 782 nm (1.58 eV), respectively, closely aligned with their computed *T*_1_ levels (1.49 eV for both; Fig. 3d). This agreement validates the computational model while demonstrating that interchromophore exciton coupling reduces triplet-state energies. Notably, trimer 3 and tetramer 4 displayed bathochromically shifted phosphorescence spectra relative to dimer 2, yet their emission peaks converged near 824 nm (1.50 eV), approaching the theoretical limit of 1.40 eV (Fig. 3d). This spectral stabilization suggests triplet-state localization as the conjugation length increases, with energy levels saturating around 1.50 eV. This value demonstrates close agreement with the saturation energy level (1.58 eV, Fig. 3c) derived from intramolecular exciton coupling analysis, revealing that the S_1_ and T_1_ energy levels asymptotically converge toward a unified threshold as the number of coupled BODIPY units increases.

The adiabatic energy gap between the S_1_ and T_1_ states was determined to decrease significantly from 0.248 eV in dimer 2 to 0.048 eV, as revealed by low-temperature photoluminescence and phosphorescence emission measurements. Such a small energy gap may facilitate the ISC process. Furthermore, the SOC matrix elements (*ξ*) for the S_1_–T_1_ channel were estimated for all oligomers, with values of 0.0658 cm^−1^ of 2, 23.908 cm^−1^ of 2Br, 0.0177 cm^−1^ of 3, and 0.0264 cm^−1^ of 4, respectively (Table S6). These results indicate that the bromine atoms effectively enhance the SOC strength. Additionally, the ISC rate was evaluated using eqn (2):2
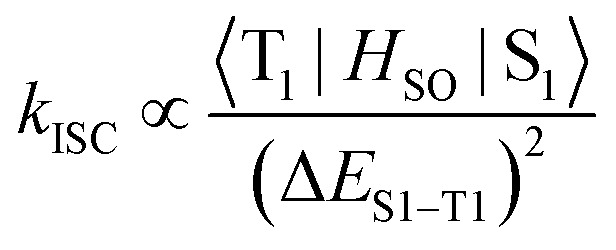


The results show that the ISC rate of 2Br is significantly faster than that of the other oligomers, suggesting that 2Br should exhibit a higher ISC efficiency.

### Triplet-state spin density distribution and energy

To elucidate this localization mechanism, we mapped triplet-state spin density distributions (Fig. 3d). Monomer (1) and dimers (2, 2Br) showed delocalized spin density across all BODIPY units, where extended conjugation progressively lowers triplet energy through electron cloud redistribution. However, in compounds 3 and 4, spin density became confined to a central BODIPY core and adjacent pyrrole groups, unchanged with further oligomer extension. This abrupt transition to spatial localization—independent of conjugation length—reveals a critical threshold in triplet-state delocalization for extended BODIPY architectures.

Triplet-state localization has emerged as a novel molecular design strategy for precise regulation of excited-state energy levels, with recent advancements demonstrating its feasibility. Börjesson *et al.* first observed this phenomenon in a C–C single-bond-coupled BODIPY-An tetramer, where steric hindrance enforced an orthogonal arrangement between subunits to achieve triplet-state confinement.^68^ However, this geometric constraint concurrently suppressed singlet-state electronic coupling and delocalization, resulting in limited absorption redshift from 509 nm (monomer) to 586 nm (tetramer). In an alternative approach, C–C double-bond-linked dimers incorporating triplet-state restrictors localized triplet excitons within individual BODIPY units while enabling singlet-state delocalization across the entire dimeric system. This strategy achieved a more pronounced absorption shift from 534 nm (monomer) to 634 nm (dimer). Despite these successes, both methodologies faced intrinsic limitations: restricted π-conjugation lengths prevented absorption spectra from exceeding 700 nm.

Our work addresses this challenge through steric-minimized C–C single-bond coupling, yielding near-planar BODIPY oligomers with rigid aromatic conjugation frameworks. This design simultaneously accomplishes: spatially confined triplet states *via* controlled electronic localization and unprecedented spectral extension with absorption maxima surpassing 700 nm. Notably, this contrasts with benzene-anthracene-fused BODIPY systems reported elsewhere.^34,74^ The strong aromaticity inherent to fused benzene/anthracene moieties fundamentally disrupts triplet localization capabilities, highlighting the critical role of conjugation topology in excited-state engineering.

### Transient absorption and triplet-state lifetimes

**Fig. 4:**
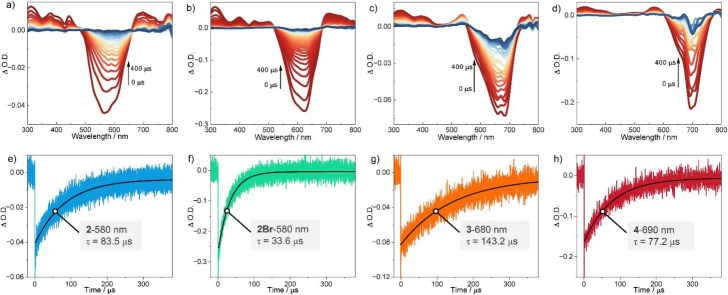
The nanosecond transient absorption spectra of 2 (a), 2Br (b), 3 (c) and 4 (d). The decay kinetic curves of 2 at 647 nm (e), 2Br at 756 nm (f), 3 at 800 nm (g) and 4 at 822 nm (h), [oligo-BODIPYs] = 0.02 mM, *λ*_ex_ = 532 nm (Pulse width 8 ns, 10 Hz), chloroform as solvent.

The triplet-state characteristics of BODIPY oligomers were then investigated using transient absorption spectroscopy (TAS). As shown in Fig. 4, TAS of BODIPYs 2, 2Br, 3, and 4 in chloroform were recorded under 532 nm excitation. All four compounds exhibited analogous spectral features. For 2 (Fig. 4a), four positive bands at 311 nm, 378 nm, 442 nm, and 699 nm were assigned to excited-state absorption (ESA) transitions. A broad negative signal spanning 504–645 nm correlated precisely with the steady-state absorption profile (Fig. 3a), confirming ground-state bleaching (GSB). TAS demonstrated identical temporal evolution for all spectral features (Fig. 4a), unambiguously linking them to triplet-state dynamics. Triplet-state lifetimes (*τ*_T_) were quantified *via* exponential fitting at 580 nm (Fig. 4e), yielding *τ*_T_ = 83.5 µs for 2.

Compound 2Br (Fig. 4b) displayed ESA maxima at 313 nm, 400 nm, 450 nm, and 707 nm, with GSB centered at 622 nm. Despite matching kinetic coherence across all bands, 2Br exhibited a significantly shorter *τ*_T_ = 33.6 µs, attributable to enhanced spin–orbit coupling (SOC) between T_1_ and S_0_ states induced by bromine's heavy-atom effect. Higher oligomers 3 and 4 manifested analogous ESA signatures, with GSB signals redshifted to 690 nm and 705 nm, respectively—consistent with their steady-state spectra (Fig. 3a). Kinetic analysis at 680 nm (3) and 690 nm (4) revealed exceptionally long *τ*_T_ values of 143.2 µs and 77.2 µs, respectively. This longevity arises from a critical balance: interchromophore exciton coupling enhances S_1_ → T_1_ SOC^92–94^ while maintaining weak T_1_ → S_0_ SOC, thereby suppressing non-radiative decay pathways.

Interestingly, the increase in conjugation length results in the absorption peaks extending into the NIR region (>700 nm), while the oligo-BODIPYs still maintain long triplet lifetimes. Therefore, those oligomers demonstrate remarkably efficient energy transfer capabilities to ground-state molecular oxygen (^3^O_2_), generating potent reactive oxygen species (^1^O_2_) with elevated oxidative potentials. This mechanistic pathway enables critical applications in photodynamic therapy and photooxidation catalysis through controlled singlet oxygen sensitization.

### Evaluation of ROS efficiency and type

**Fig. 5:**
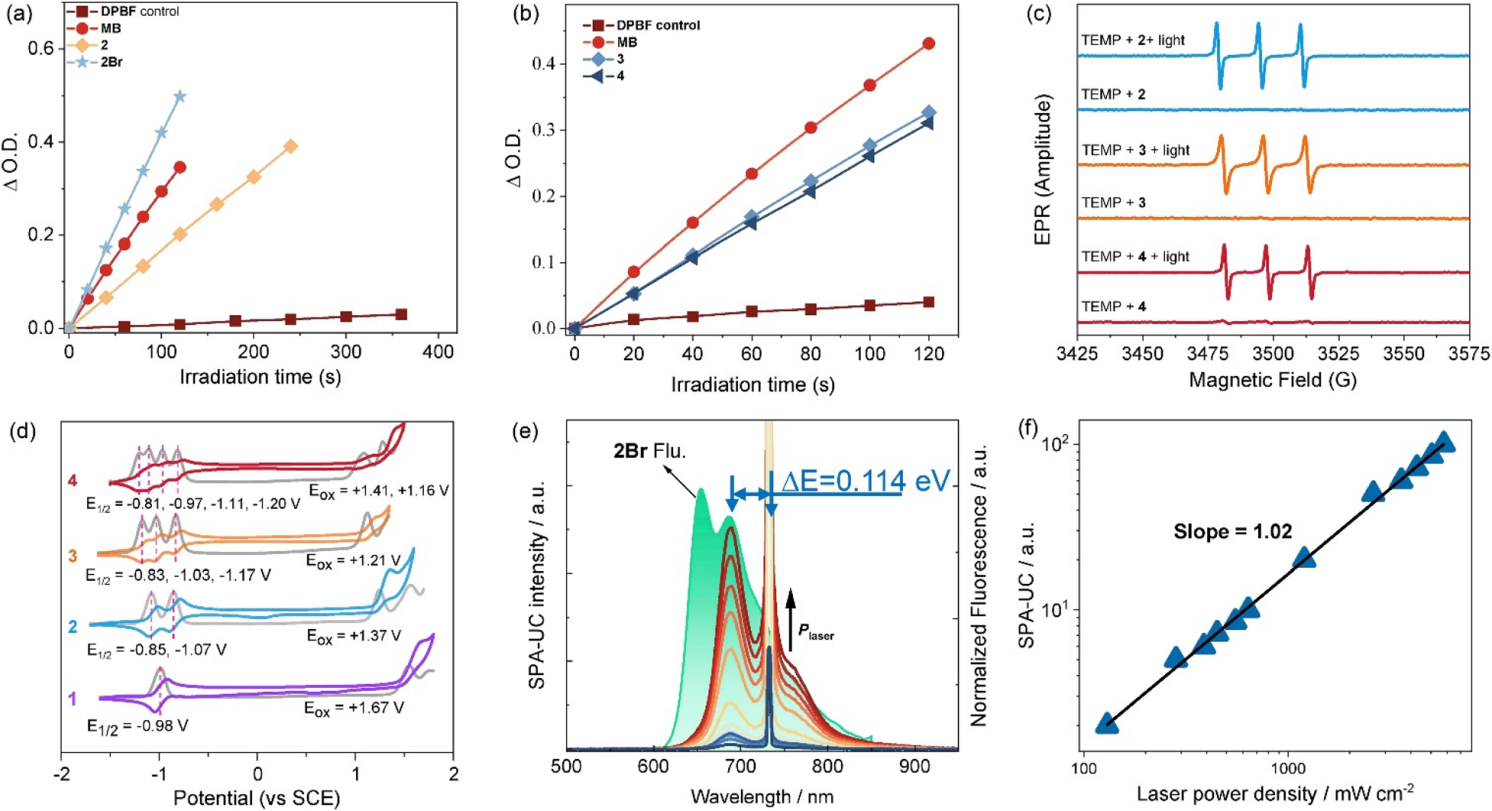
Photochemical properties of BODIPY oligomers. Comparative DPBF degradation profiles (absorbance at 415 nm) in chloroform by dimers 2 and 2Br (a), and trimer and tetramer (b). (c) EPR spectra of 2–4 (1.0 mM in chloroform) with TEMP (50 mM) as the spin-trap agent under different conditions. (d) Cyclic voltammograms and differential pulse voltammetry of BODIPYs 1–4 in dichloromethane at rt. (e) SPA-UC emission intensity as a function of excitation power density of 2Br. (f) Double logarithmic plot of the SPA-UC intensity as a function of excitation power density, [2Br] = 0.02 mM, toluene as solvent.

Subsequently, the classic 1,3-diphenylisobenzofuran (DPBF) degradation assay^66–69,95^ was performed to evaluate the reactive oxygen species (ROS) generation ability (Fig. 5a, b and Table 1). Selecting methylene blue (MB) as a reference, a ROS quantum yield of 0.28 for dimer 2 was determined in chloroform. Significantly, trimer 3 and tetramer 4 achieved enhanced ROS yields with the same value of 0.36, underscoring their superior performance as NIR-absorbing photosensitizers. Interestingly, although there is a significant difference in the triplet lifetimes of compounds 3 and 4, their ROS generation efficiencies are comparable. This is mainly due to the long triplet lifetimes of the photosensitizers, which result in nearly 100% triplet energy transfer efficiency with ground-state oxygen in the solvent. Strikingly, brominated dimer 2Br demonstrated significantly higher ROS yield of 0.71, attributable to the heavy-atom effect boosting ISC process. Moreover, electron paramagnetic resonance (EPR) spectroscopy was used to confirm triplet-state-mediated ROS generation in representative oligomers 2–4. The observed EPR signals corresponding to the paramagnetic adduct (Fig. 5c and S16–18), confirmed that these oligomers undergo ISC process upon photoexcitation, thereby populating the triplet excited state. The subsequent quenching primarily proceeds *via* an energy-transfer pathway for generating singlet oxygen (^1^O_2_, type-II), with a minor contribution from electron transfer leading to superoxide radical formation (O_2_˙^−^, type-I).

### Energy of charge separated state from electrochemical data

Cyclic voltammetry (CV) of these oligomers was investigated (Fig. 5d). The monomer, dimer, trimer and tetramer showed one, two, three and four reversible reduction peaks, respectively, with gradually increased potentials from −0.98 V to −0.81 V. The splitting reductions indicated strong electronic communications between the BODIPY subunits in these oligomers, which were clearly confirmed by the well-separated reduction peaks from differential pulse (DPV) voltammetry (Fig. S21). On the oxidation side, a marked escalation in oxidative vulnerability was evidenced by the decrease from 1.67 V for monomer 1 to 1.16 V for tetramer 4. Moreover, further electrochemical investigation revealed that the energies of charge separated states (*E*_cs_)^73^ are well influenced by oligomerization and involving solvents (Table S5), and calculated energies of charge *E*_cs_ are consistent with corresponding findings of excited state absorption. Notably, oligomers 2–4 displayed the stabilized Gibbs free energy changes of charge separation (Δ*G*_cs_) with calculated values of 0.14 eV, which well supported the observable fluorescence emission capability in the experimental studies.

### Application of single-photon absorption-based upconversion

Single-photon absorption-based upconversion (SPA-UC) arises from the thermal excitation of high vibrational levels (*v* > 0) in the ground electronic state (S_0_), enabling absorption of low-energy photons and subsequent emission of higher-energy photons.^96^ This mechanism holds promise for optical refrigeration and bioimaging applications^97,98^ Effective SPA-UC materials require rich vibrational substructures, as evidenced by the significant spectral overlap between absorption and emission profiles of these BODIPY oligomers (Fig. 3a). This overlap correlates with increased vibrational mode density in extended π-conjugated systems, motivating our investigation of their SPA-UC properties.

As demonstrated for dimer 2Br (Fig. 5e and f), fluorescence emission in toluene exhibits two distinct vibronic peaks at 654 nm (*v*_S_1__ = 0 → *v*_S_0__ = 0) and 687 nm (*v*_S_1__ = 0 → *v*_S_0__ = 1). Under 733 nm excitation, intense emission at 687 nm precisely matches the *v*_S_1__ = 0 → *v*_S_0__ = 1 transition, confirming a unique SPA-UC pathway: *v*_S_0__ = 1 → *v*_S_1__ = 0 photon absorption followed by *v*_S_1__ = 0 → *v*_S_0__ = 1 emission. The kinetic process of this SPA-UC differs from previously reported SPA-UC systems (*e.g.*, DAES-C, CzBN, BNOSe),^96,99,100^ which typically exhibit *v*_S_0__ = 1 → *v*_S_1__ = 0 absorption coupled with *v*_S_1__ = 0 → *v*_S_0__ = 0 emission. The unconventional vibronic progression in these BODIPY oligomers expands the operational scope of SPA-UC materials while enriching the mechanistic framework for photon upconversion processes.

## Conclusions

In conclusion, we have developed a series of covalently β,β-linked BODIPY oligomers *via* a regioselective Pd(ii)-catalyzed oxidative C–H cross-coupling protocol. The head-to-tail arrangement in chromophore arrays induces intense intramolecular J-type exciton coupling. Owing to the minimal steric hindrance between BODIPY units, the oligomers adopt a planar configuration, significantly lowering singlet-state energies while preserving triplet-state energies, thereby enabling precise modulation of the singlet-triplet energy gaps. These oligomers exhibit tunable absorption and emission extended to NIR region, good fluorescence quantum yields, and large Stokes shifts, alongside exceptional triplet properties including long-lived triplet lifetimes and efficient ROS generation of both ^1^O_2_ and O_2_˙^−^. Furthermore, dimer 2Br serves as a prototypical example to demonstrate the potential of these BODIPY oligomers in high-efficiency SPA-UC applications. This work establishes intramolecular exciton coupling as a versatile strategy to design heavy-atom-free photosensitizers of conjugated BODIPY oligomers with progressively reduced HOMO–LUMO bandgaps and Δ*E*_S–T_ values, driven by reduced singlet state energies without significant triplet state perturbation. These oligomers show simultaneous NIR emission and high triplet yields, unlocking potentials in biomedicine, photocatalysis, and beyond.

## Author contributions

Z. K., E. H. and L. J. conceived and directed the overall project. C. P., F. L. and X. Z. performed the synthetic experiments and data characterization. C. P. and J. S. performed the steady and transient state spectral characterization. X. X. and Y. W. performed the theoretical calculations and analysed the results. J. G. contributed to data analysis and discussions. Z. K., Y. W., E. H. and L. J. wrote the paper with input from all other authors. All authors discussed the results and commented on the manuscript.

## Conflicts of interest

There are no conflicts to declare.

## Supplementary Material

SC-017-D5SC08201C-s001

## Data Availability

Supplementary information (SI): experimental details, including theoretical calculations, spectroscopic measurements, detailed synthesis and characterization of all products reported in this study; NMR and HRMS spectra of all new products. See DOI: https://doi.org/10.1039/d5sc08201c.
