# Supplementary material for: Intramolecular exciton coupling modulates the convergent singlet-triplet energy gap toward NIR-emissive heavy-atom-free Oligo-BODIPY photosensitizers
Source: Chem Sci. 2025 Dec 9;17(6):3267–77. doi: 10.1039/d5sc08201c (PMC12716148; doi:10.1039/d5sc08201c)
Supplement: SC-017-D5SC08201C-s001 [file SC-017-D5SC08201C-s001.pdf]

Supplementary Information (SI) for Chemical Science.
This journal is © The Royal Society of Chemistry 2025

Supporting Information for

Intramolecular Exciton Coupling Modulates the Convergent Singlet-Triplet Energy Gap toward NIR-Emissive Heavy-Atom-Free oligo-BODIPY Photosensitizers

Chunyan Pan,[a] Jinsong Shao,[a] Zhengxin Kang,*[a,b] Fan Lv,[a] Xiankang Zhang,[a] Jiangang Gao,[b] Xinsheng Xu,[a] Yaxiong Wei,*[a] Erhong Hao,*[a] and Lijuan Jiao*[a]

[a]*Key Laboratory of Functional Molecular Solids of Ministry of Education, College of Chemistry and Materials Science, School of Physics and Electronic Information, Anhui Normal University, Wuhu, 241002, China.*

[b]*School of Chemical and Environmental Engineering, Anhui Polytechnic University, Wuhu, 241000, China*

**To whom correspondence should be addressed.*

E-mail: kangzx@mail.ahpu.edu.cn, Davidl@mail.ustc.edu.cn, haoehong@ahnu.edu.cn, jiao421@ahnu.edu.cn

Contents

1. General information 1
2. Synthesis and characterization 3
3. Photophysical data 8
4. Reactive oxygen species (ROS) properties 15
5. Calculations of excitonic couplings 18
6. Electrochemical data 19
7. DFT calculations 22
8. ^{1}H and ^{13}C NMR spectra for all new compounds 44
9. High resolution mass spectra for all new compounds 51
10. Reference 55

1. General information

Reagents and solvents were used as received from commercial suppliers (Energy Chemicals, Shanghai, China) unless noted otherwise. All reactions were performed in oven-dried or flame-dried glassware unless stated otherwise and were monitored by TLC using 0.25 mm silica gel plates with UV indicator (60F-254). ^{1}H and ^{13}C NMR were recorded on a 400 MHz NMR spectrometer at room temperature. Chemical shifts (δ) are given in ppm relative to $CDCl_3$ (7.26 ppm for ^{1}H and 77.16 ppm for ^{13}C) to internal TMS. High-resolution mass spectra (HRMS) were obtained using ESI-TOF or MALDI-TOF in positive mode.

Absorption and emission measurements. UV-visible absorption and fluorescence emission spectra were recorded on commercial spectrophotometers (Shimadzu UV-2450 and Edinburgh FS5 spectrometers). All measurements were made at 25 °C, using 5 × 10 mm cuvettes. Non-degassed, spectroscopic grade solvents and a 10 mm quartz cuvette were used. Absolute fluorescence quantum efficiencies of BODIPY derivatives were measured by absolute PL quantum yield spectrometer (Hamamatsu, C11347) in integrating sphere, using Equation (S1) given below[1]:

$$\Phi_F = \frac{N_{em}}{N_{abs}} = \frac{\alpha\int\frac{\lambda}{hc}I_{em}(\lambda)\mathrm{d}\lambda}{\alpha\int\frac{\lambda}{hc}[I_{em}(\lambda)-I'_{ex}(\lambda)]\mathrm{d}\lambda}\text{....................(S1)}$$

where N_{em} and N_{abs} are the numbers of emitted and absorbed photons, respectively, α is the calibration factor for the measurement setup, λ is the wavelength, h is the Plank's constant, c is the speed of light, I_{em} (λ) is the emission intensity at λ, I_{ex} (λ) and I'_{ex} (λ) are the intensities of the excitation laser beam with λ in the absence and presence of the sample, respectively. The measured Φ_F value is independent of sharp and thickness of sample and power of excitation laser.

Electrochemical measurements. Cyclic voltammograms were obtained on a CH Instruments electrochemical workstation (CHI 610E, USA). Cyclic voltammograms of 1.0 mM BODIPYs **1-4** were measured in dichloromethane solution, containing 0.1 M tetrabutylammonium hexafluorophosphate ($TBAPF_6$) as the supporting electrolyte, glassy carbon electrode as a working electrode, Pt wire as a counter electrode, and saturated calomel electrode (SCE) as reference electrode at 100 mV s^{-1} of scanning rate at room temperature. Before each experiment, the working electrode was polished on a felt pad with 0.3 μm alumina (Buehler, Ltd., Lake Bluff, IL) and sonicated in Milli-Q deionized water and then in ethanol. The counter and reference electrodes were cleaned by rinsing and sonicating in water, and ethanol.

Reactive oxygen species (ROS) generation efficiency. A comparative study of the relative singlet oxygen generating efficiency of these dyes was performed in air-saturated solvents under light at 635

nm and 660 nm laser irradiation condition using 1,3-diphenylisobenzofuran (DPBF, 4×10^{-5} M) as a trap molecule.[2] A commercial photosensitizer against Methyl Blue (**MB**) was used as reference.[3] The absorbance of BODIPY dyes and the reference **MB** at 635 nm or 660 nm was kept around 0.15. The decrease of the absorbance band of DPBF at 415 nm was monitored. Reactive oxygen species quantum yield (Φ_Δ) determinations were carried out using the chemical trapping method, and the Φ_Δ value was obtained by the relative method using **MB** as the reference as shown in following Equation (S2).

$$\Phi_{\Delta sam} = \Phi_{\Delta ref}[(m_{sam}/m_{ref})(L_{ref}/L_{sam})] \quad \text{...(S2)}$$

Where $\Phi_{\Delta ref}$ and $\Phi_{\Delta sam}$ are the singlet oxygen quantum yields for the standard **MB** and photosensitizer (**2**, **2Br**, **3** and **4**). m_{sam} and m_{ref} are the slope of the difference (Δ_{OD}) in the change in the absorption maximum wavelength of DPBF (415 nm), which are plotted against the photoirradiation time, L_{ref} and L_{sam} are the light harvesting efficiency, which is given by $L = 1 - 10^{-A}$ (“A” is the absorbance at the laser irradiation wavelength 635 nm or 660 nm).

Electron paramagnetic resonance (EPR) experiments. CW X-band EPR spectra for radicals were acquired on Bruker EMX instrument. EPR spectra of various mixtures were recorded after irradiation at room temperature.

Transient absorption spectra. Nanosecond time-resolved transient absorption spectra and decay kinetics were measured on LFP instrument (LP 980, Edinburgh Instruments Ltd). The pump laser beam and the probe beam crossed perpendicularly through the liquid sample in a quartz cuvette (10 mm × 10 mm). 532 nm laser pulses as light source (10 Hz, 5.0 mJ/pulse) which were delivered by the second harmonic of a Nd: YAG laser (Surelite II-10, Continum Inc.). A dynamic decay curve was recorded with a digital phosphor oscilloscope (TDS 3012C, Tektronix Inc.). All the solutions were degassed by purging with high purity nitrogen (99.99%) for about 30 minutes prior to measurements to ensure complete removal of oxygen.

2. Synthesis and characterization

Synthesis of precursor **BDP-OH**

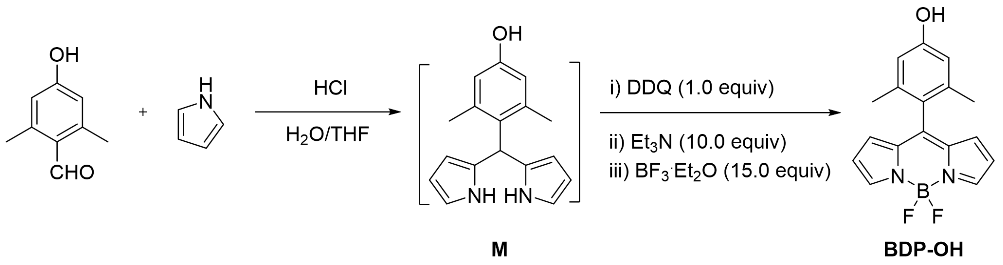

To 180 mL of aqueous HCl (2/180, v/v) was added dropwise a solution of 2,6-dimethyl-4-hydroxybenzaldehyde (3.0 g, 20 mmol) in THF (60 mL) at room temperature. Followed by the addition of the pyrrole (4.0 mL, 60 mmol). The reaction mixture was stirred at room temperature and the reaction progress was monitored by TLC. After 60 h, the THF was evaporated to dryness, resulting in a solid precipitate that often adhered to the flask walls. The aqueous phase was then removed, followed by thrice extractions using ethyl acetate (3 × 150 mL). The organic layer was collected, dried over anhydrous Na_2SO_4, and organic solvent was evaporated under vacuum. The crude product **M**, obtained from this step, was immediately utilized in the subsequent reaction. THF (80 mL) was employed as the solvent, to which DDQ (2.7 g, 12 mmol) was added. The reaction mixture was stirred at room temperature for 2 h. Subsequently, triethylamine (17 mL, 120 mmol) was added. The reaction mixture was stirred at room temperature for 1 h. Boron trifluoride ether (23 mL, 180 mmol) was slowly added under an ice water bath. The reaction mixture was then stirred at room temperature for 8 h. Upon completion, the crude product was purified by column chromatography (silica; petroleum ether/ethyl acetate = 4:1, v/v) to give the red powder **BDP-OH** in 26% isolated yield (1.6 g). ^{1}H NMR (400 MHz, $CDCl_3$) δ 7.91 (s, 2H), 6.70 (d, J = 4.0 Hz, 2H), 6.63 (s, 2H), 6.48 (d, J = 2.8 Hz, 2H), 5.07 (brs, 1H), 2.08 (s, 6H). ^{13}C NMR (100 MHz, $CDCl_3$) δ 156.2, 147.5, 144.4, 138.5, 135.8, 130.4, 125.1, 118.7, 114.4, 20.3. HRMS (ESI) m/z calcd for $C_{17}H_{16}BF_2N_2O$ $[M + H]^+$: 313.1324, found 313.1322.

Synthesis of BODIPY **1**

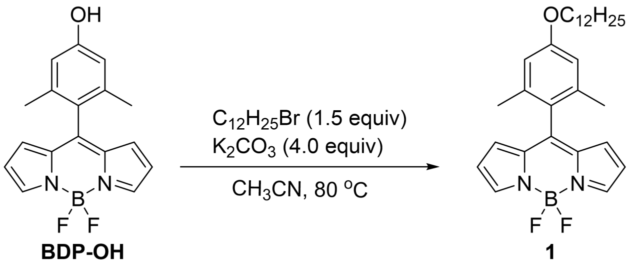

To a 100 mL round-bottom flask, **BDP-OH** (1.0 g, 2.1 mmol), K_2CO_3 (1.2 g, 8.4 mmol) and 1-bromododecane (554 μL, 2.3 mmol) were dissolved in acetonitrile (20.0 mL). The reaction mixture was stirred at 80 °C for 4 h. Upon completion, the cooled reaction mixture was transferred to a 1000 mL separatory funnel, then extracted thrice with dichloromethane (3 × 100 mL). The organic layer was collected, dried over anhydrous Na_2SO_4, and organic solvent was evaporated under vacuum. The crude product was purified by column chromatography (silica; petroleum ether/dichloromethane = 2:1, v/v) to give the powder **1** in 88% isolated yield (888 mg). ^{1}H NMR (400 MHz, $CDCl_3$) δ 7.90 (s, 2H), 6.70 (d, J = 4.0 Hz, 2H), 6.68 (s, 2H), 6.47 (d, J = 3.2 Hz, 2H), 3.99 (t, J = 6.8 Hz, 2H), 2.11 (s, 6H), 1.85 – 1.78 (m, 2H), 1.55 – 1.45 (m, 2H), 1.37 – 1.28 (m, 16H), 0.89 (t, J = 6.4 Hz, 3H). ^{13}C NMR (100 MHz, $CDCl_3$) δ 159.6, 147.7, 144.3, 138.1, 135.9, 130.4, 124.9, 118.6, 113.5, 68.1, 32.1, 29.8(2), 29.7(9), 29.7(6), 29.7, 29.6, 29.5(0), 29.4(5), 26.2, 22.8, 20.5, 14.3. HRMS (ESI) m/z calcd for $C_{29}H_{40}BF_2N_2O$ $[M + H]^+$: 481.3202, found 481.3210.

Synthesis of BODIPY **1Br**

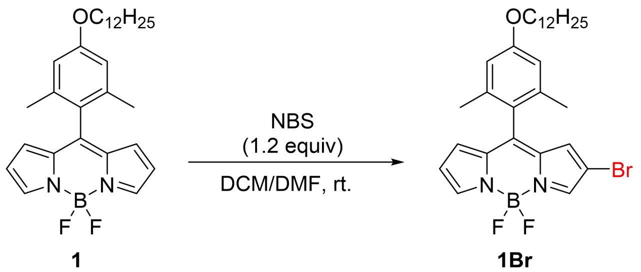

To a solution of BODIPY **1** (480 mg, 1.0 mmol) in DMF/DCM (10 mL/10 mL) was added dropwise a solution of NBS (214 mg, 1.20 mmol) in DCM (5 mL) at room temperature. The mixture was stirred at room temperature for 4 h. The reaction was monitored by TLC analysis, which finally indicated the presence of **1Br** as a major product. The resulting mixture was quenched with a saturated solution of sodium thiosulfate ($Na_2S_2O_3$). Then the reaction mixture was transferred to a

500 mL separatory funnel, then extracted thrice with dichloromethane (3×50 mL). The organic layer was collected, dried with anhydrous Na_2SO_4. After removal of solvents in vacuo, the mixture was purified by silica-gel column chromatography (silica; petroleum ether/dichloromethane = 4:1, v/v) to give the powder **1Br** in 78% isolated yield (435 mg). ^{1}H NMR (400 MHz, $CDCl_3$) δ 7.97 (s, 1H), 7.75 (s, 1H), 6.78 (d, J = 4.0 Hz, 1H), 6.67 (s, 2H), 6.64 (s, 1H), 6.53 (d, J = 4.4 Hz, 1H), 3.98 (t, J = 6.8 Hz, 2H), 2.10 (s, 6H), 1.84 – 1.77 (m, 2H), 1.56 – 1.45 (m, 2H), 1.37 – 1.27 (m, 16H), 0.89 (t, J = 6.8 Hz, 3H). ^{13}C NMR (100 MHz, $CDCl_3$) δ 159.8, 147.7, 146.5, 142.4, 138.1, 136.5, 135.2, 132.0, 129.0, 124.3, 119.8, 113.6, 111.9, 68.1, 32.1, 29.8(2), 29.7(9), 29.7(6), 29.7, 29.5(4), 29.5(0), 29.4, 26.2, 22.8, 20.5, 14.3. HRMS (ESI) m/z calcd for $C_{29}H_{38}BBrFN_2O$ [M - F]$^+$: 539.2245, found 539.2250.

Synthesis of dimer **2Br**

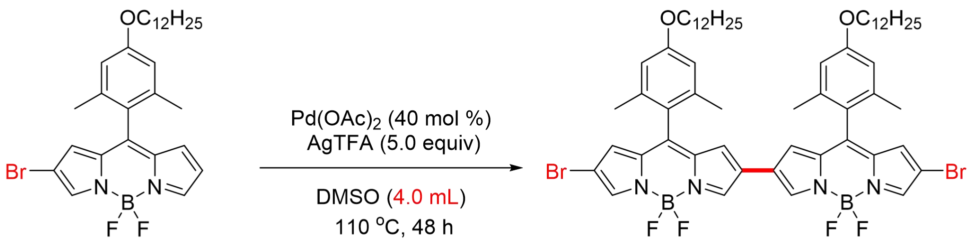

1Br **2Br**, 38%

To a 35 mL tube, BODIPY **1Br** (168 mg, 0.3 mmol), $Pd(OAc)_2$ (27 mg, 0.12 mmol) and AgTFA (331 mg, 1.5 mmol) were dissolved in DMSO (4.0 mL). The reaction mixture was stirred at 110 °C for 48 h. Upon completion, the reaction mixture was transferred to a 500 mL separatory funnel, then extracted thrice with dichloromethane (3 × 50 mL). The organic layer was collected and washed twice with water, dried over anhydrous Na_2SO_4, and organic solvent was evaporated under vacuum. The crude product was purified by column chromatography (silica; petroleum ether/dichloromethane = 2:1, v/v) to give the powder **2Br** in 38% isolated yield (64 mg).^{1}H NMR (400 MHz, $CDCl_3$) δ 8.11 (s, 2H), 7.77 (s, 2H), 6.69 (s, 4H), 6.68 (s, 2H), 6.66 (s, 2H), 3.99 (t, J = 6.0 Hz, 4H), 2.11 (s, 12H), 1.85 – 1.78 (m, 4H), 1.52 – 1.45 (m, 4H), 1.40 – 1.28 (m, 32H), 0.89 (t, J = 6.8 Hz, 6H); ^{13}C NMR (100 MHz, $CDCl_3$) δ 160.0, 147.3, 143.6, 143.3, 138.1, 136.8, 135.7, 129.4, 127.0, 124.4, 124.0, 113.7, 106.8, 68.1, 32.1, 29.8(4), 29.8(1), 29.7(7), 29.7(5), 29.6, 29.5, 29.4, 26.3, 22.9, 20.6, 14.3. HRMS (MALD-TOF) m/z: calcd for $C_{58}H_{74}B_2Br_2F_4N_4O_2^+$ [M]$^+$ 1116.4300, found 1116.4329.

Synthesis of BODIPY oligomers **2-4**

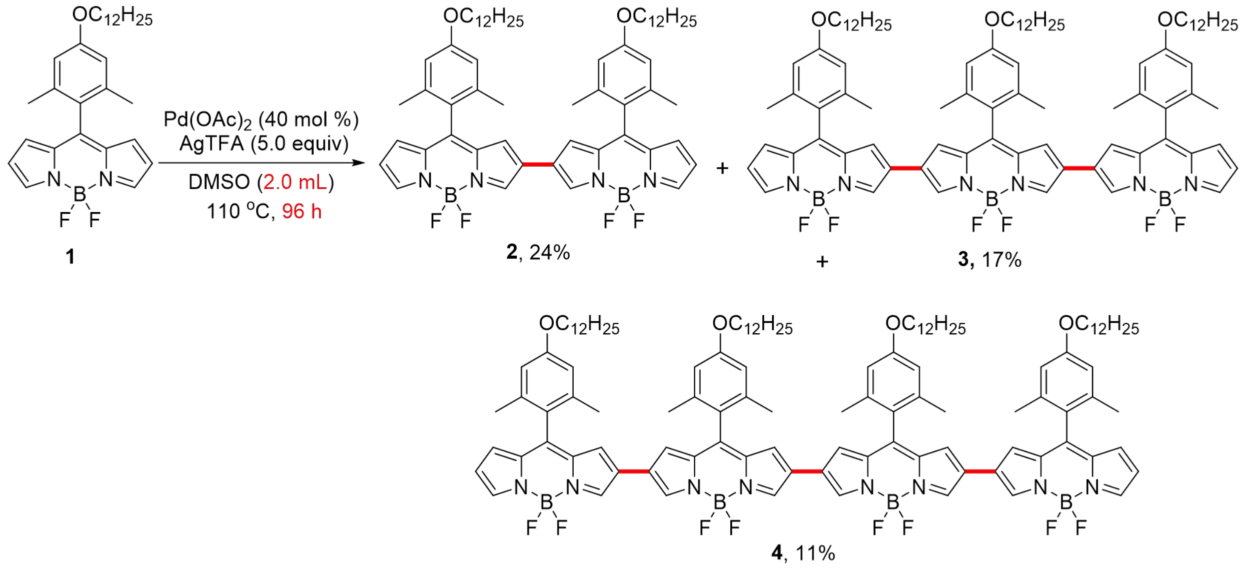

To a 35 mL tube, BODIPY **1** (144 mg, 0.3 mmol), $Pd(OAc)_2$ (27 mg, 0.12 mmol) and AgTFA (331 mg, 1.5 mmol) were dissolved in DMSO (2.0 mL). The reaction mixture was stirred at 110 °C for 96 h. Upon completion, the reaction mixture was transferred to a 500 mL separatory funnel, then extracted thrice with dichloromethane (3×50 mL). The organic layer was collected and washed twice with water, dried over anhydrous Na_2SO_4, and organic solvent was evaporated under vacuum. The crude product was purified by column chromatography (silica; petroleum ether/dichloromethane = 1:1-1:3, v/v) to give the powder dimer **2** (35 mg, 24%), trimer **3** (24 mg, 17%) and tetramer **4** (16 mg, 11%).

2: ^{1}H NMR (400 MHz, $CDCl_3$) δ 8.05 (s, 2H), 7.91 (s, 2H), 6.70 (d, J = 5.6 Hz, 6H), 6.62 (s, 2H), 6.48 (d, J = 2.4 Hz, 2H), 4.00 (t, J = 6.8 Hz, 4H), 2.11 (s, 12H), 1.85 – 1.78 (m, 4H), 1.53 – 1.46 (m, 4H), 1.38 – 1.26 (m, 32H), 0.89 (t, J = 6.4 Hz, 6H); ^{13}C NMR (100 MHz, $CDCl_3$) δ 159.7, 147.2, 144.9, 141.6, 138.1, 136.3, 130.5, 126.5, 124.7, 123.2, 118.9, 113.5, 68.1, 32.1, 29.8(4), 29.8(1), 29.7(8), 29.7(6), 29.6, 29.5(2), 29.4(7), 26.3, 22.9, 20.6, 14.3. HRMS (ESI) m/z calcd for $C_{58}H_{76}B_2F_4N_4O_2^+$ [M + H]$^+$: 959.6163, found 959.6157.

3: ^{1}H NMR (400 MHz, $CDCl_3$) δ 8.09 (s, 4H), 7.92 (s, 2H), 6.70 (d, J = 5.2 Hz, 8H), 6.60 (s, 4H), 6.48 (dd, J = 4.0, 1.6 Hz, 2H), 3.99 (t, J = 6.4 Hz, 6H), 2.11 (s, 18H), 1.85 – 1.78 (m, 6H), 1.53 – 1.47 (m, 6H), 1.38 – 1.26 (m, 48H), 0.89 (t, J = 6.4, 9H); ^{13}C NMR (100 MHz, $CDCl_3$) δ 159.8, 159.7, 147.0, 146.2, 145.0, 142.6, 142.0, 138.2, 136.7, 136.3(3), 136.2(7), 130.5, 126.9, 126.5, 124.8, 124.6, 123.2, 123.1, 119.0, 114.2, 113.5, 68.1, 32.1, 29.8(4), 29.8(1), 29.7(8), 29.7(6), 29.6, 29.5(2),

29.4(7), 26.3, 22.9, 20.6, 14.3. HRMS (MALD-TOF) m/z: calcd for $C_{87}H_{113}B_3F_6N_6O_3^+$ $[M]^+$ 1436.9086, found 1436.9081.

4: ^{1}H NMR (400 MHz, $CDCl_3$) δ 8.57 (s, 6H), 8.08 (s, 2H), 6.70 (d, J = 6.4 Hz, 10H), 6.60 (d, J = 8.4 Hz, 6H), 6.52 (d, J = 4.0 Hz, 2H), 3.99 (d, J = 6.0 Hz, 8H), 2.13 (s, 24H), 1.85 – 1.79 (m, 8H), 1.48 (d, J = 6.0 Hz, 8H), 1.30 – 1.26 (m, 64H), 0.90 (t, J = 3.2 Hz, 12H). ^{13}C NMR (100 MHz, $CDCl_3$) δ 159.7, 159.6, 146.5, 145.4, 145.1, 143.3, 138.4, 138.3, 136.7, 136.4, 136.2, 130.1, 127.2, 127.0, 125.0(1), 124.9(6), 123.3, 123.1, 118.9, 113.5, 68.1, 32.1(1), 32.0(9), 29.8(7), 29.8(5), 29.8(2), 29.8(1), 29.7(9), 29.7(7), 29.6(1), 29.5(9), 29.5(5), 29.5(3), 29.5(0), 26.3(2), 26.2(8), 22.8(7), 22.8(6), 20.6(3), 20.6(0), 14.3. HRMS (MALD-TOF) m/z: calcd for $C_{116}H_{150}B_4F_8N_8O_4^+$ $[M]^+$ 1915.2084, found 1915.2076.

Scheme S1. Possible reaction mechanism.[4] (L: ligand).

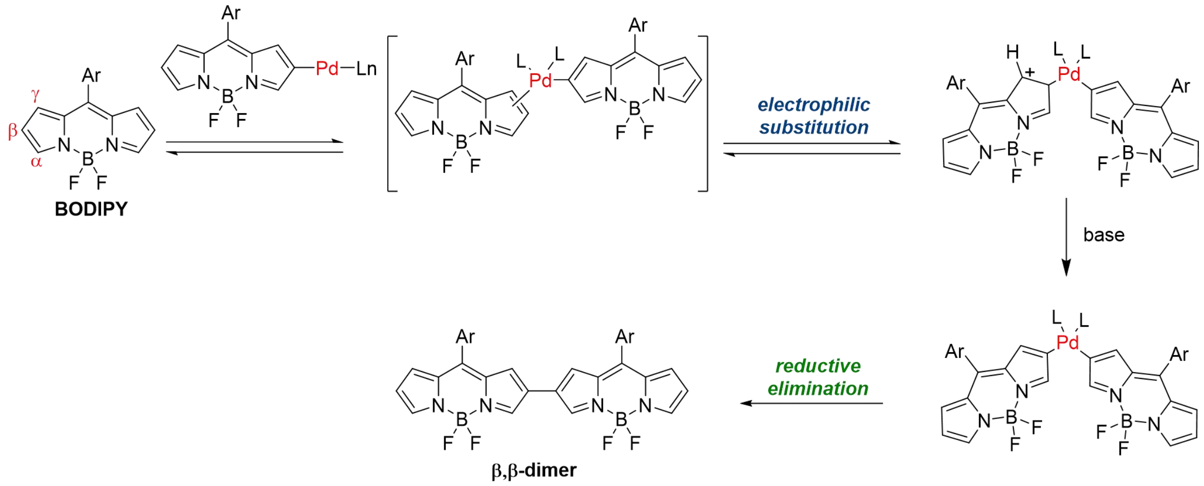

3. Photophysical data

Table S1: Spectroscopic and photophysical properties of BODIPYs in different solvents.

dyes	solvent	λ_{abs}^{max} (nm)	λ_{em}^{max} (nm)	Stokes shift (cm^{-1})	ε [a]	Φ [b]
**BDP-OH**	Hexane	499	512	510	64600	0.92
	Toluene	503	518	580	64600	0.91
	DCM	501	515	540	60300	0.47
	THF	500	515	580	61700	0.04
	MeCN	497	513	630	56200	0.02
**1**	Hexane	498	512	550	66500	0.94
	Toluene	503	518	580	64500	0.94
	$CHCl_3$	502	515	500	61800	0.85
	DCM	500	517	660	61800	0.31
	THF	500	517	660	61400	0.63
	MeCN	497	516	740	56800	0.02
**1Br**	Hexane	517	532	550	60300	0.24
	Toluene	520	538	640	58900	0.25
	DCM	517	536	690	56200	0.07
	THF	516	534	650	55000	0.09
	MeCN	513	534	770	51300	0.01
**2**	Hexane	609	658	1220	69300	0.47
	Toluene	616	668	1260	65500	0.39
	$CHCl_3$	613	659	1140	60800	0.33
	DCM	609	664	1360	58900	0.22
	THF	614	666	1270	66600	0.19
	MeCN	603	662	1480	58400	0.09
**2Br**	Hexane	627	678	1200	95000	0.31
	Toluene	634	688	1240	88600	0.27
	DCM	627	680	1240	85400	0.15
	THF	631	684	1230	86300	0.10
	MeCN	621	678	1350	77900	0.05
**3**	Hexane	665	708	910	119700	0.44
	Toluene	671	718	980	119000	0.44
	$CHCl_3$	669	722	1100	120700	0.38
	DCM	675	720	930	119700	0.33

	THF	688	720	650	125000	0.32
	MeCN	677	724	960	122300	0.05
	Hexane	670, 729	780	900	124700, 116300	0.04
	Toluene	710	750	750	176000	0.36
**4**	$CHCl_3$	706	747	780	180300	0.35
	DCM	703	751	910	178300	0.32
	THF	713	758	830	194300	0.29
	MeCN	655	780	2450	87300	0.01

[a] Corresponding to the strongest absorption maximum, the unit for ε is M^{-1} cm^{-1}. [b] Fluorescence quantum yields were measured by absolute PL quantum yield spectrometer (Hamamatsu) using integrating sphere. The standard errors are less than 5%.

UV-vis and fluorescence spectroscopies

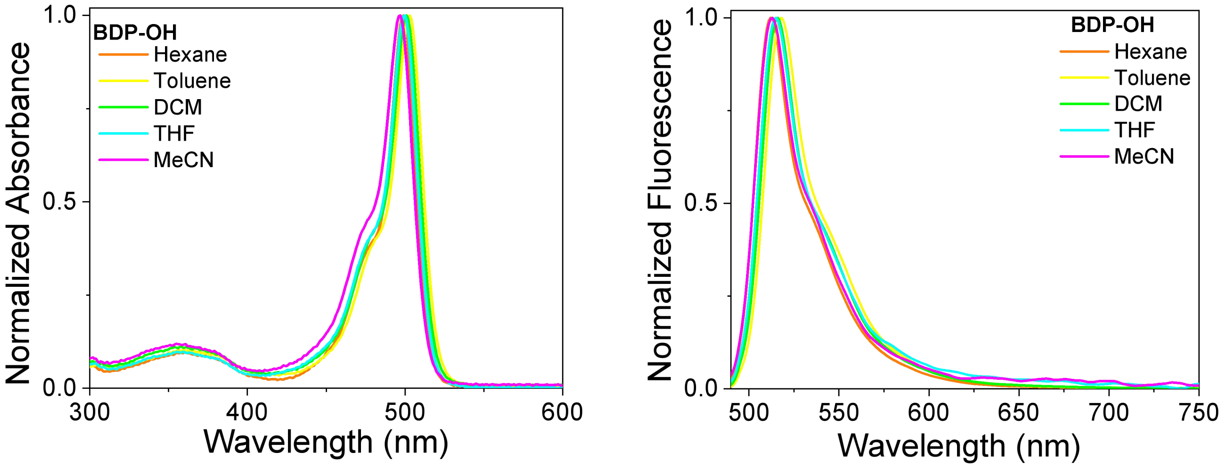

Figure S1. Absorption (left) and emission (right) spectra of compound **BDP-OH** recorded in different solvents. Excited at 480 nm.

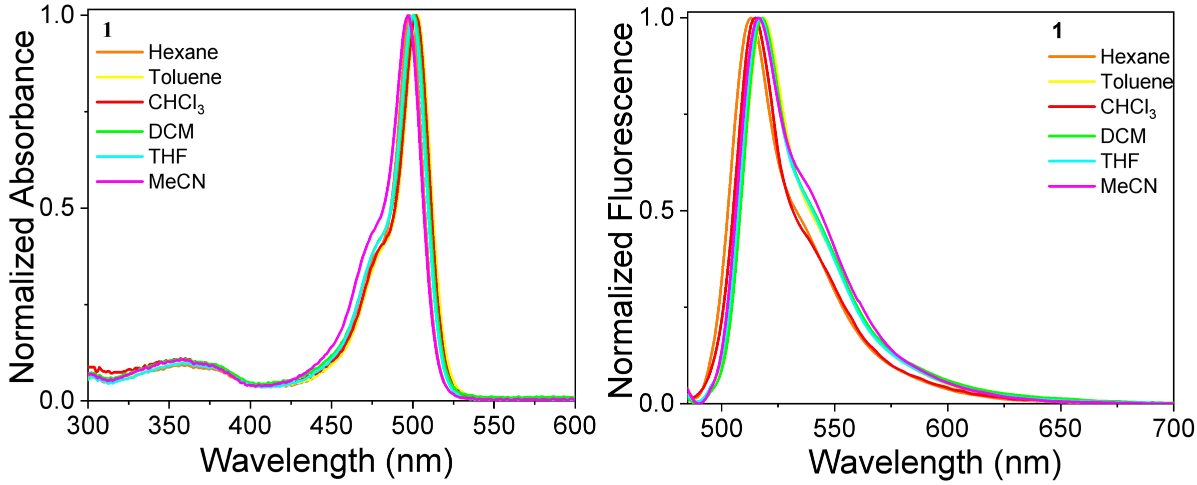

Figure S2. Absorption (left) and emission (right) spectra of compound **1** recorded in different solvents. Excited at 480 nm.

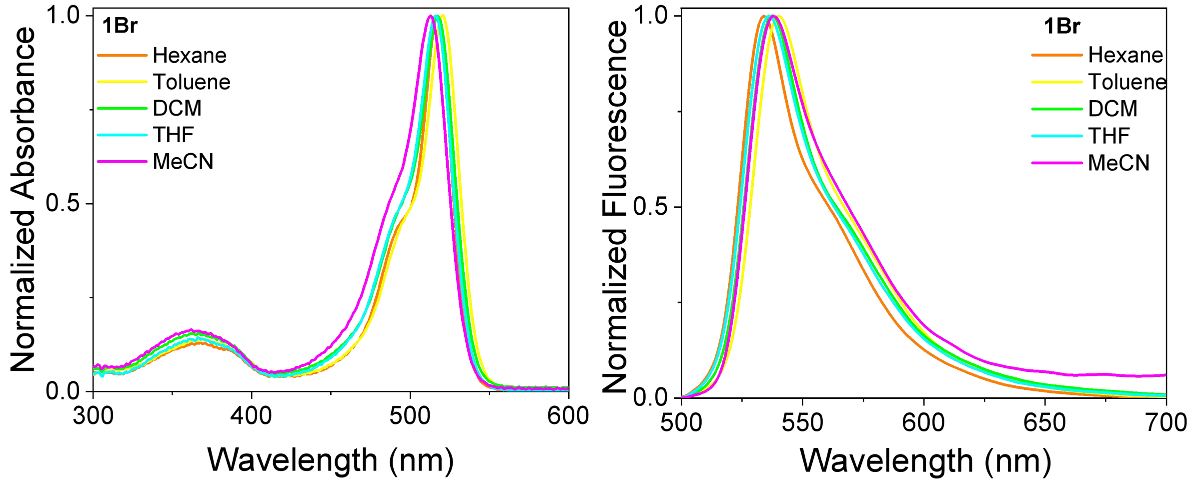

Figure S3. Absorption (left) and emission (right) spectra of compound **1Br** recorded in different solvents. Excited at 490 nm.

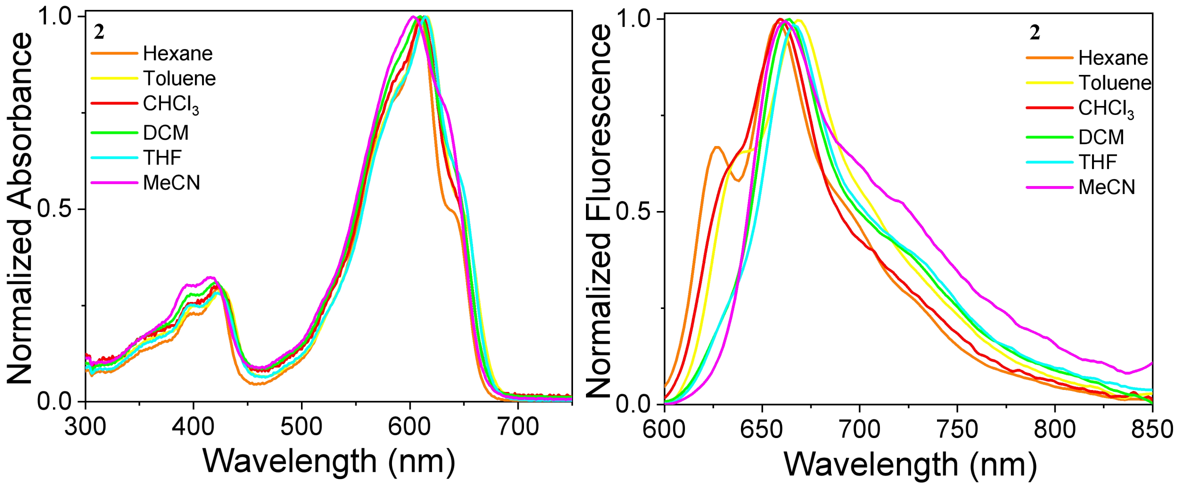

Figure S4. Absorption (left) and emission (right) spectra of compound **2** recorded in different solvents. Excited at 590 nm.

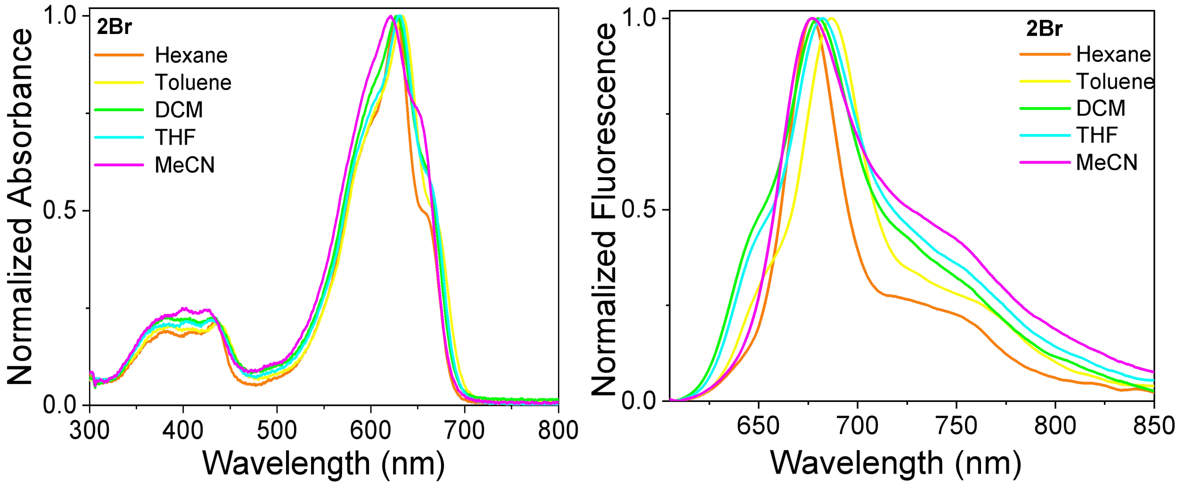

Figure S5. Absorption (left) and emission (right) spectra of compound **2Br** recorded in different solvents. Excited at 590 nm.

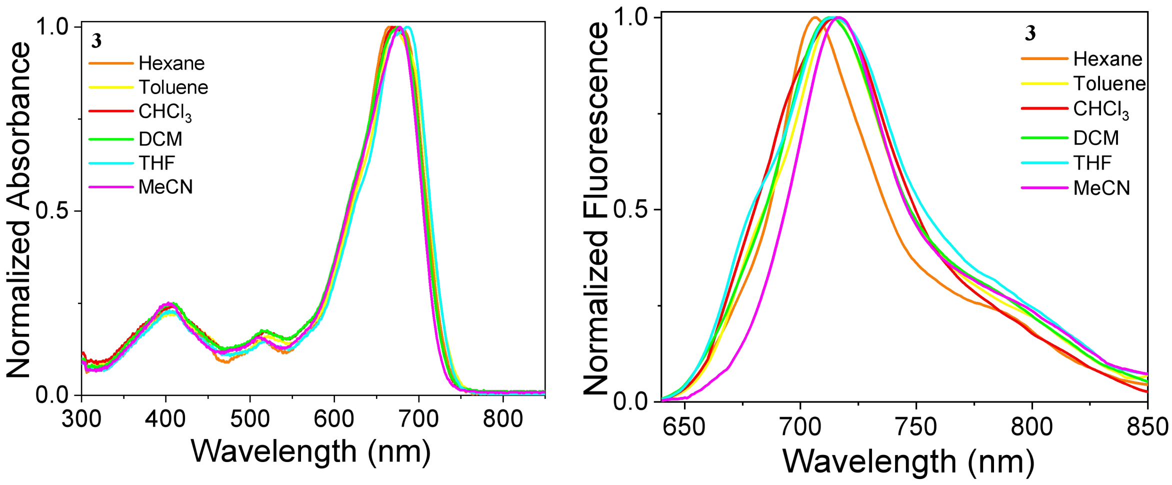

Figure S6. Absorption (left) and emission (right) spectra of compound **3** recorded in different solvents. Excited at 650 nm.

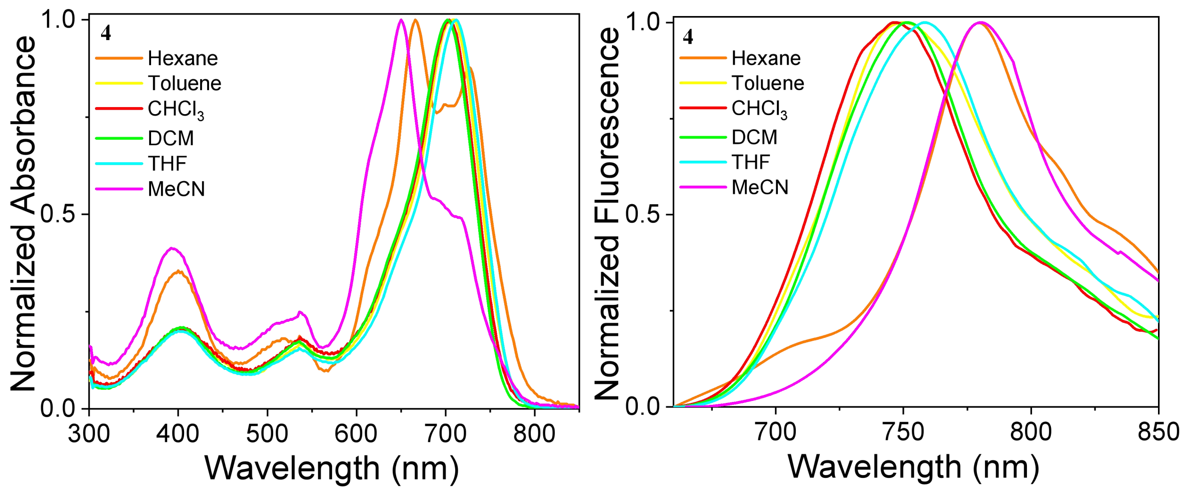

Figure S7. Absorption (left) and emission (right) spectra of compound **4** recorded in different solvents. Excited at 680 nm.

Solubilities

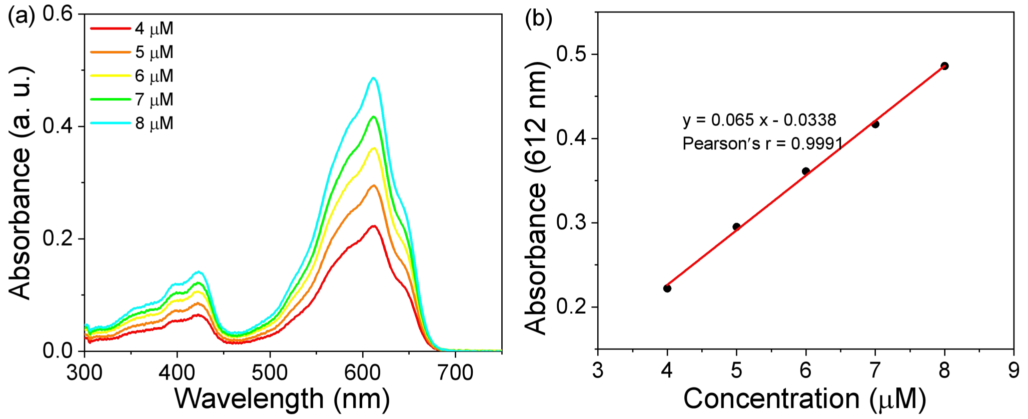

Figure S8. Absorption spectra of **2** at different concentration in chloroform. (a) Overlay of absorption spectra of different concentrations. (b) The linear relationship of absorbance at 612 nm vs concentration of **2**.

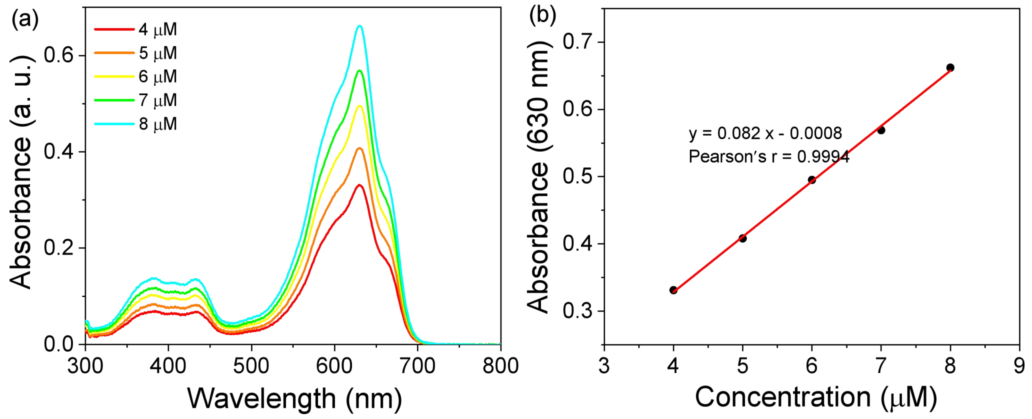

Figure S9. Absorption spectra of **2Br** at different concentration in chloroform. (a) Overlay of absorption spectra of different concentrations. (b) The linear relationship of absorbance at 630 nm vs concentration of **2Br**.

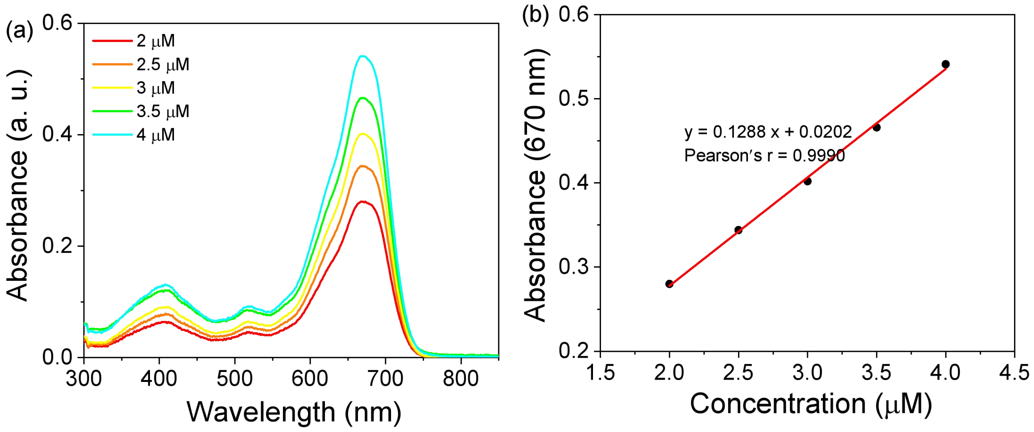

Figure S10. Absorption spectra of **3** at different concentration in chloroform. (a) Overlay of absorption spectra of different concentrations. (b) The linear relationship of absorbance at 670 nm vs concentration of **3**.

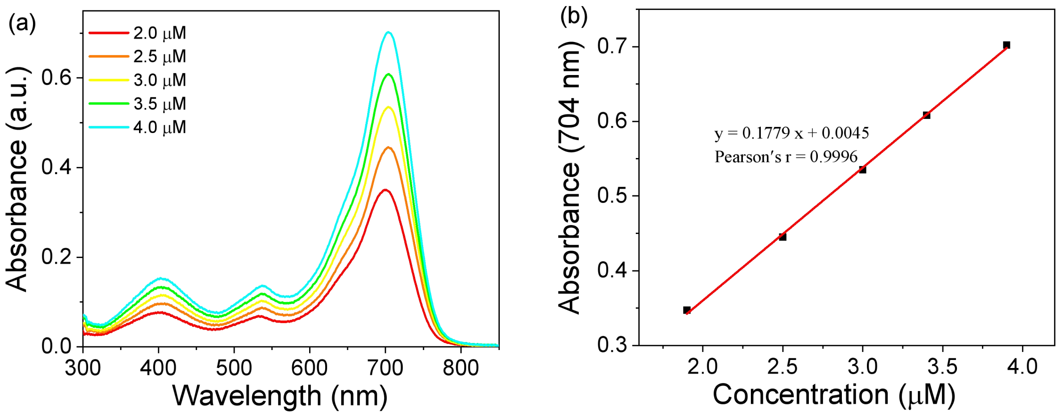

Figure S11. Absorption spectra of **4** at different concentration in chloroform. (a) Overlay of absorption spectra of different concentrations. (b) The linear relationship of absorbance at 704 nm vs concentration of **4**.

4. Reactive oxygen species (ROS) properties

Table S2. Relative efficiency of reactive oxygen quantum yield of BODIPY oligomers in $CHCl_3$.

dyes	Φ_Δ
**2**	0.28
**2Br**	0.71
**3**	0.36
**4**	0.36

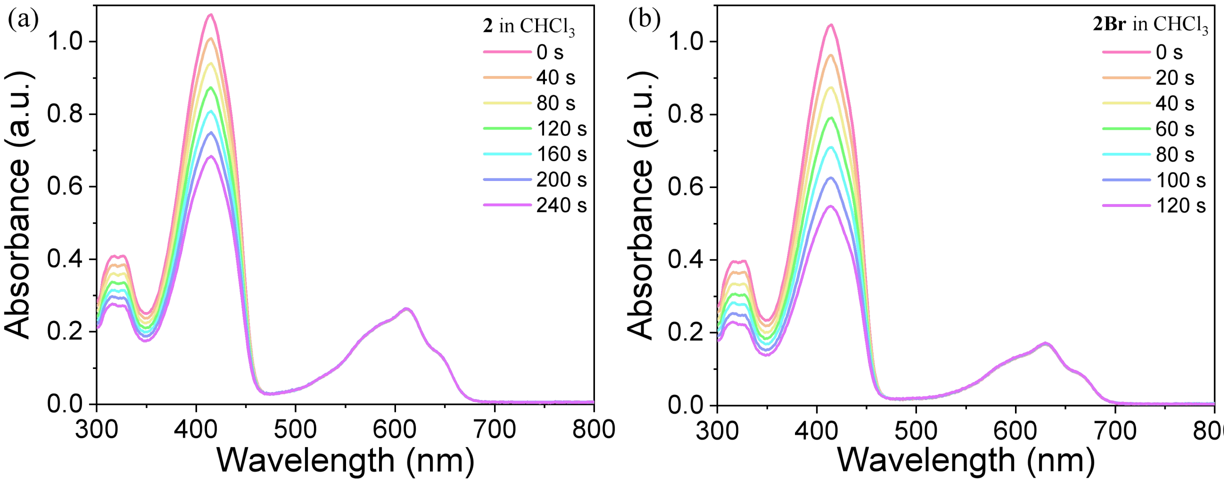

Figure S12. Absorption spectra of DPBF (initial absorbance around 1.0 at 415 nm) upon irradiation in the presence of BODIPY all dimers with initial absorbance around 0.15 at 635 nm in chloroform. (a) **2** for 240 s (recorded at 40 s intervals) in chloroform. (b) **2Br** for 120 s (recorded at 20 s intervals) in chloroform.

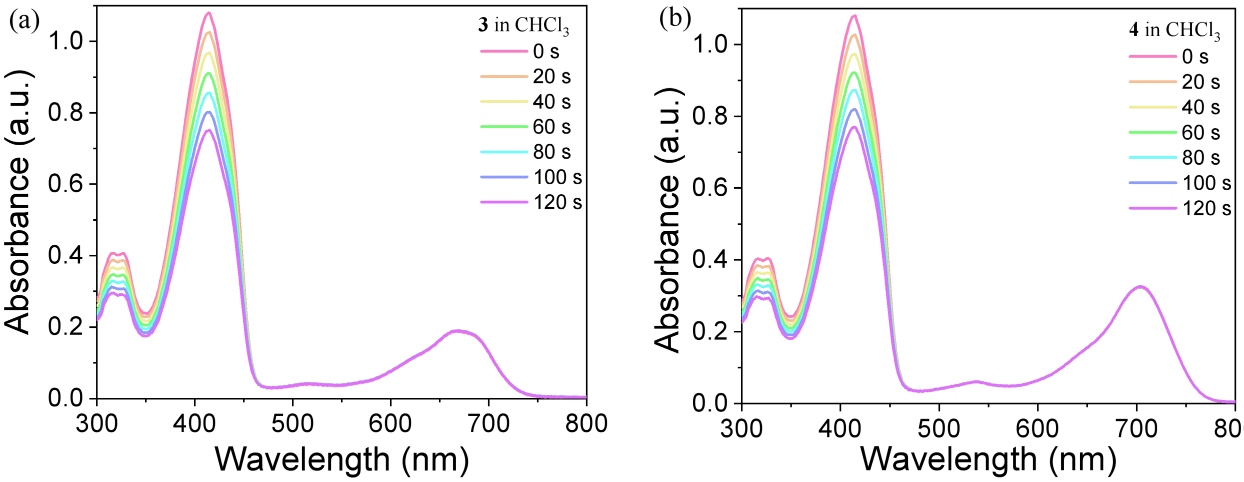

Figure S13. Absorption spectra of DPBF (initial absorbance around 1.0 at 415 nm) upon irradiation in the presence of BODIPYs **3** and **4** with initial absorbance around 0.15 at 660 nm in chloroform. (a) **3** for 120 s (recorded at 20 s intervals) in chloroform. (b) **4** for 120 s (recorded at 20 s intervals) in chloroform.

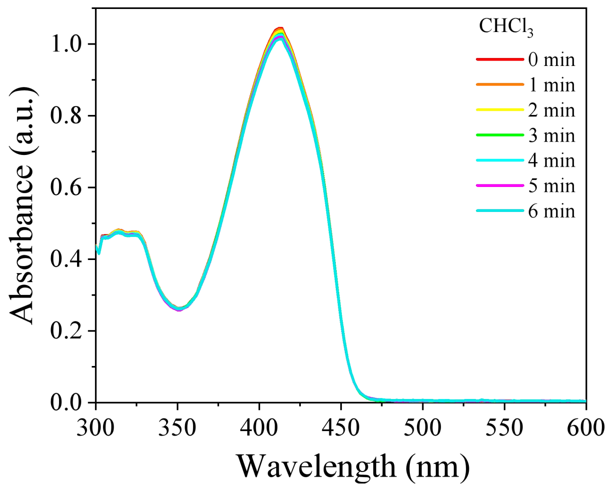

Figure S14. Control experiments: absorption spectra of DPBF (initial absorbance around 1.1 at 415 nm) after 6 min irradiation with a 635 nm laser in $CHCl_3$.

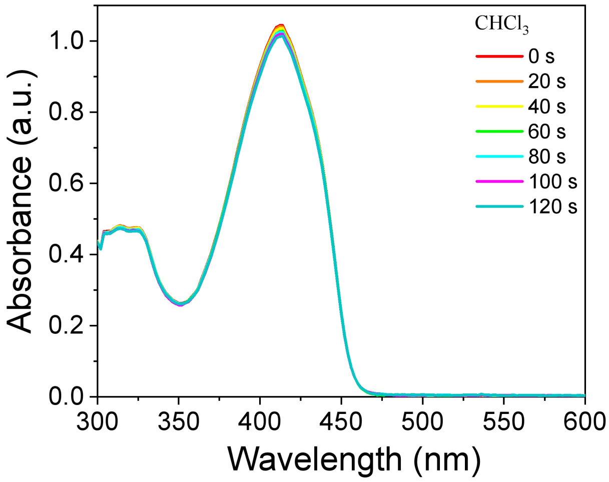

Figure S15. Control experiments: absorption spectra of DPBF (initial absorbance around 1.1 at 415 nm) after 120 s irradiation with a 660 nm laser in $CHCl_3$.

EPR characterizes the type of reactive oxygen species

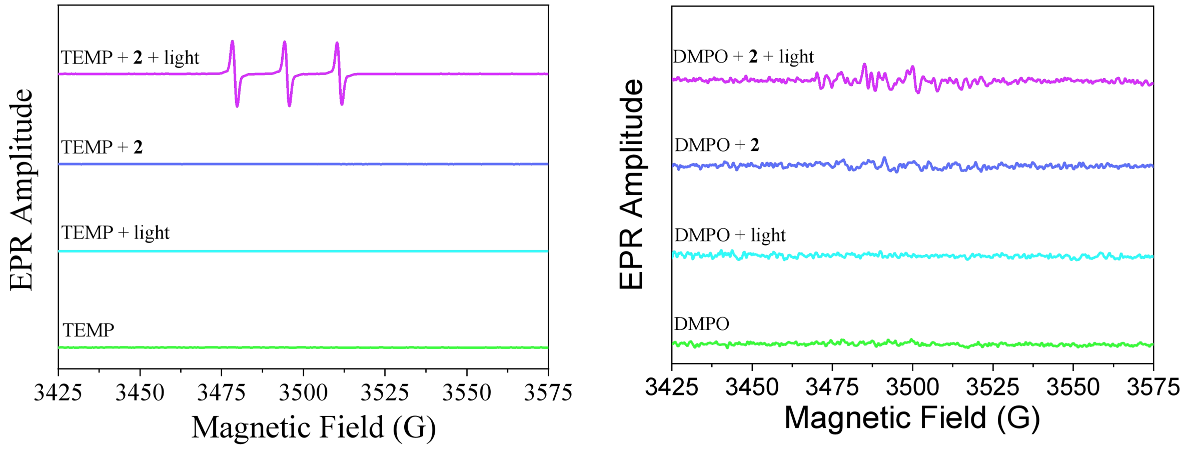

Figure S16. EPR spectra of **2** (1.0 mM in chloroform) for $^1O_2$ characterization with 2,2,6,6-tetramethyl-4-piperidone (TEMP, 50 mM) or for $O_2^{\bullet -}$ characterization with 5,5-dimethyl-1-pyrroline-*N*-oxide (DMPO, 200 mM).

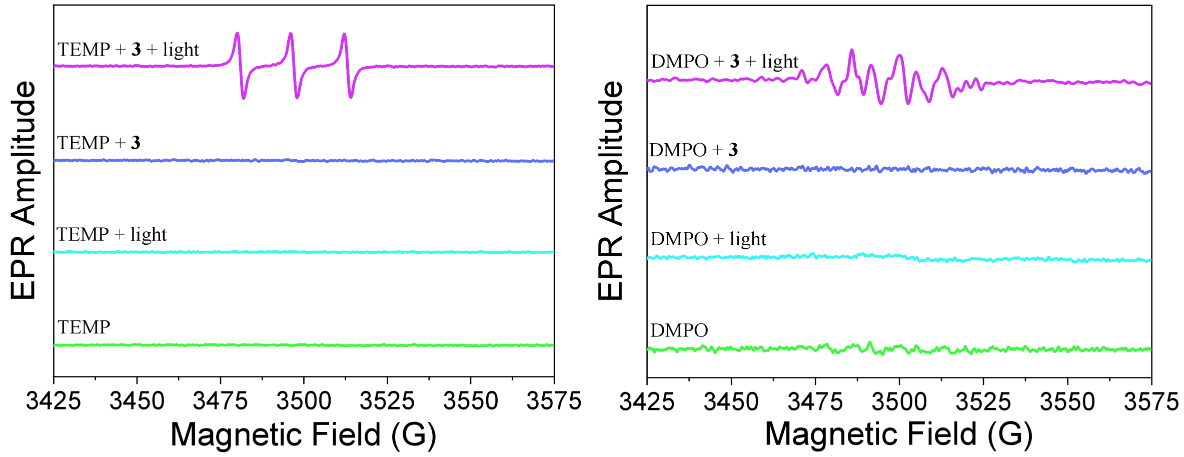

Figure S17. EPR spectra of **3** (1.0 mM in dichloromethane) for $^1O_2$ characterization with 2,2,6,6-tetramethyl-4-piperidone (TEMP, 50 mM) or for $O_2^{\bullet -}$ characterization with 5,5-dimethyl-1-pyrroline-*N*-oxide (DMPO, 200 mM).

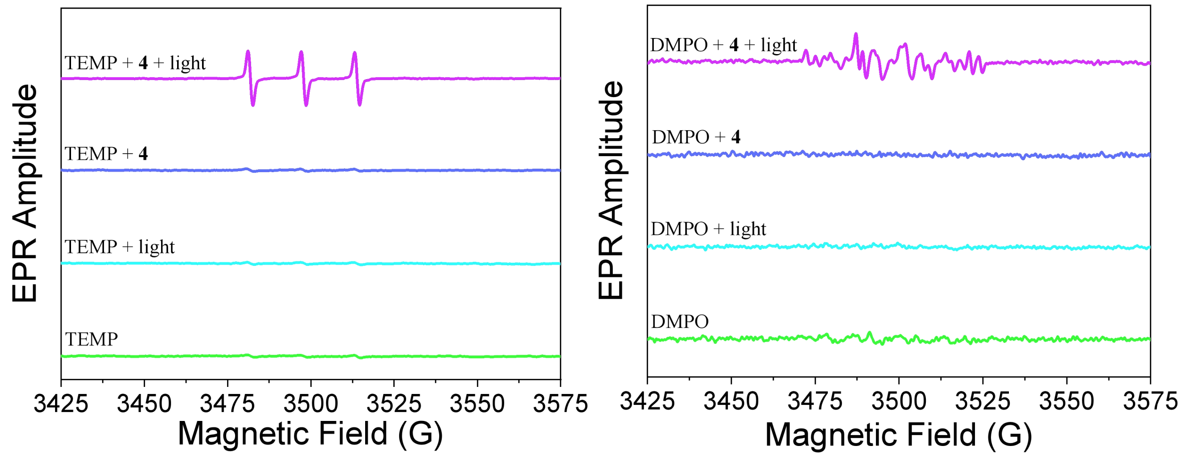

Figure S18. EPR spectra of **4** (1.0 mM in dichloromethane) for $^1O_2$ characterization with 2,2,6,6-tetramethyl-4-piperidone (TEMP, 50 mM) or for $O_2^{\bullet -}$ characterization with 5,5-dimethyl-1-pyrroline-*N*-oxide (DMPO, 200 mM).

5. Calculations of excitonic couplings

The oscillator strength f was calculated according to equation (S3) with $\varepsilon(\tilde{\nu})$ being the attenuation coefficient as a function of the wavenumber $\tilde{\nu}$.[5a]

$$f = 4.319 \cdot 10^{-9} \int \varepsilon(\tilde{\nu})\, d\tilde{\nu} \qquad \text{(S3)}$$

Transition dipole moments were obtained from equation (S4) with h being Planck's constant (6.63 × 10^{-34} J s), c is the speed of light (3 × 10^{10} cm s^{-1}), ε_0 is the electric field constant (8.8542 × 10^{-12} C^2 J^{-1} m^{-1}), n is the refractive index of the solvent, N_{Av} is the Avogadro constant (6.02 × 10^{23} mol^{-1}) and $\varepsilon(\tilde{\nu})$ is the attenuation coefficient as a function of the wavenumber $\tilde{\nu}$ (except for c, $\varepsilon(\tilde{\nu})$) and $\tilde{\nu}$, all constants in SI units). one Debye (D) = 3.3356 × 10^{-30} C m.[5]

$$\mu_{eg}{}^2 = \frac{3hc\varepsilon_0 \cdot \ln(10) \cdot 9n}{2000\pi^2 \cdot N_{Av} \cdot (n^2+2)^2} \cdot \int \frac{\varepsilon(\tilde{\nu})d\tilde{\nu}}{\tilde{\nu}} \qquad \text{(S4)}$$

Table S3. Calculated values of oscillator strength and transition dipole moment for BODIPYs in toluene.

dyes	f	μ_{eg} (×10^{-29} C m)	μ_{eg} (D)[a]	$\mu_{eg}{}^2$ (D^2)
**1**	0.58	2.22	6.66	44.36
**2**	1.33	3.54	10.63	113.00
**2Br**	1.64	4.01	12.01	144.24
**3**	2.45	4.95	14.86	220.82
**4**	3.05	5.63	16.89	285.27

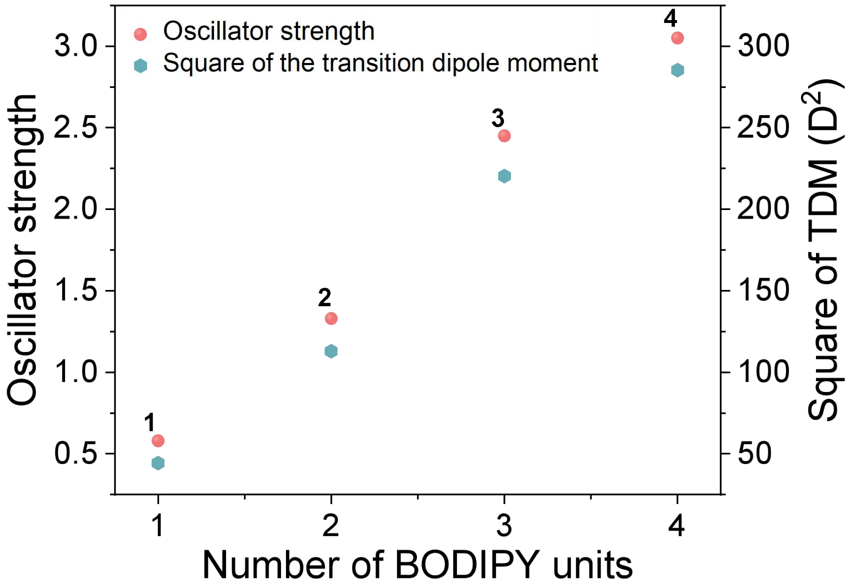

Figure S19. Oscillator strength and the squared transition dipole moment (TDM) of compounds **1-4**.

6. Electrochemical data

Table S4. Electrochemical data acquired at 100 mV/s, and HOMO-LUMO Gaps determined from spectroscopy of dyes 1-4 [a].

dyes	$E_{1/2}^{red}$ (V)	E_{red}^{onest} (V)	E_{pa}^{ox} (V)	E_{ox}^{onest} (V)	LUMO (eV)	HOMO (eV)	E_g^e (eV)	E_g^o (eV)
**1**	- 0.98	-0.91	1.67	1.44	- 3.49	- 5.84	2.35	2.35
**2**	- 0.85; - 1.07	-0.75	1.37	1.19	- 3.65	- 5.59	1.94	1.98
**3**	- 0.83; - 1.03; - 1.17	-0.72	1.21	1.08	- 3.68	- 5.48	1.80	1.82
**4**	- 0.81; - 0.97; - 1.11; - 1.20	-0.70	1.41; 1.16	0.98	- 3.70	- 5.38	1.68	1.65

[a] $E_{1/2}^{red}$ = reversible reduction peak potentials; E_{pa}^{ox} = irreversible oxidation peak potentials; E_{red}^{onset} = the onset reduction potentials; E_{ox}^{onset} = the onset oxidation potentials; E_{LUMO} = -e (E_{red}^{onset} + 4.4); E_{HOMO} = -e (E_{ox}^{onset} + 4.4); E_g^e = bandgap, obtained from the intercept of the electrochemical data; E_g^e = E_{LUMO} - E_{HOMO}; E_g^o = bandgap, obtained from the intercept of the absorption spectra.

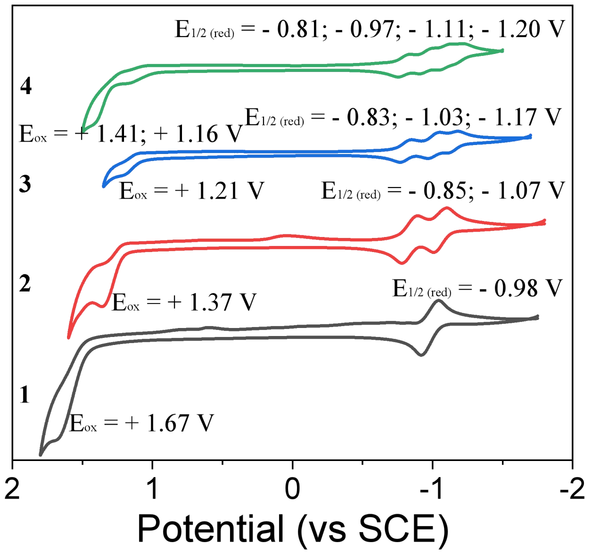

Figure S20. Cyclic voltammograms (CV) of **1-4** measured in dichloromethane solution containing 0.1 M $TBAPF_6$ as the supporting electrolyte at room temperature. Glassy carbon electrode as a working electrode, and the scan rate at 100 mV s^{-1}.

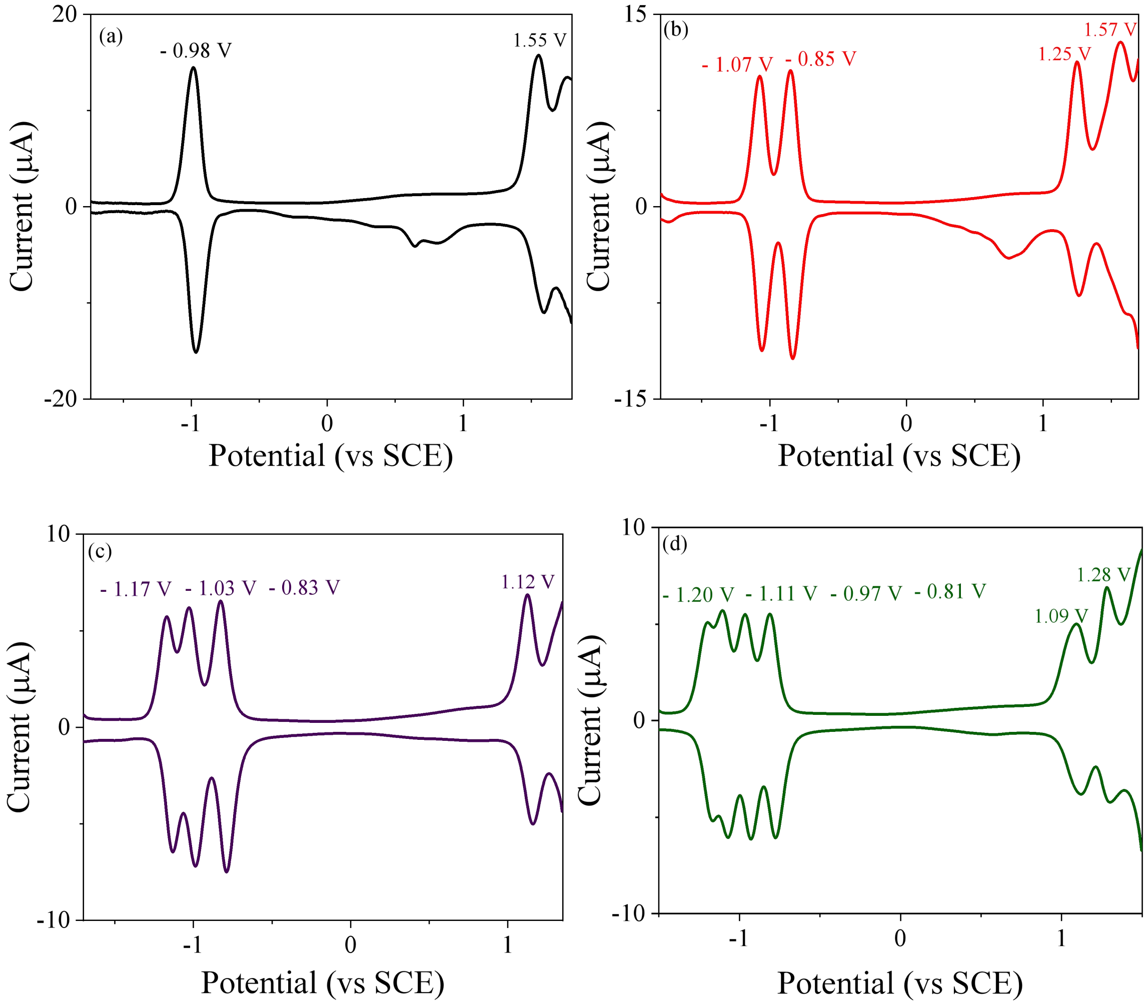

Figure S21. Differential pulse voltammograms (DPV) of **1** (a), **2** (b), **3** (c) and **4** (d) measured in dichloromethane solution containing 0.1 M $TBAPF_6$ as the supporting electrolyte at room temperature. Glassy carbon electrode as a working electrode, and the scan rate at 100 mV s^{-1}.

The Gibbs free energy changes of charge separation (ΔG_{cs}) were calculated with Rehm-Weller Equation (S5). The energy levels of the charge separated states (E_{cs}) can be calculated with Equation (S6), where the static Coulombic energy (ΔG_s) can be calculated using Equation (S7).[6]

$$\Delta G_{CS} = e[E_{ox} - E_{red}] - E_{00} + \Delta G_S \quad \text{(S5)}$$

$$E_{CS} = e[E_{ox} - E_{red}] + \Delta G_S \quad \text{(S6)}$$

$$\Delta G_S = -\frac{e^2}{4\pi\varepsilon_S\varepsilon_0 R_{CC}} - \frac{e^2}{8\pi\varepsilon_0}\left(\frac{1}{R_D} + \frac{1}{R_A}\right)\left(\frac{1}{\varepsilon_{ref}} - \frac{1}{\varepsilon_S}\right) \quad \text{(S7)}$$

Where E_{ox} = half-wave potential for one-electron oxidation of the electron-donor unit (*meso*-aryl$^{0/\bullet+}$), E_{red} = half-wave potential for one-electron reduction of the electron-acceptor moiety (BODIPY $^{0/\bullet-}$), determined by the data of differential pulse voltammograms (DPV). E_{00} is the energy level of singlet excited state with the cross point of the normalized absorption and fluorescence emission spectra. ε_S = static dielectric constant of the solvent, R_{CC} = center-to-center separation distance between the electron donor (*meso*-aryl) and electron acceptor (BODIPY), determined by the structural analysis from single-crystal X-ray diffraction analysis[7] (R_{CC} = 4.32 Å), R_D is the radius of the electron donor (1.39 Å), R_A is the radius of the electron acceptor (2.96 Å), ε_{ref} is the static dielectric constant of the solvent used for the electrochemical studies, e is the elementary charge (1.602 × 10^{-19} C). ε_0 is permittivity of free space (8.854 × 10^{-12} C^2 J^{-1} m^{-1}).

The solvent used in the calculation of free energy of the electron transfer is, *n*-hexane (ε_S = 1.88), toluene (ε_S = 2.38), DCM (ε_S = 8.93), THF (ε_S = 7.58) and MeCN (ε_S = 37.5).

Table S5. Calculated charge separation free energy (ΔG_{CS}) and charge separation state energies (E_{CS}) for BODIPYs 1-4.

	E_{00} (eV)	ΔG_{CS} (eV)					E_{CS} (eV)				
		hexane	toluene	DCM	THF	MeCN	hexane	toluene	DCM	THF	MeCN
**1**	2.43	0.23	0.18	0.06	0.07	0.03	2.65	2.61	2.49	2.50	2.46
**2**	1.92	0.31	0.26	0.14	0.15	0.11	2.22	2.18	2.06	2.07	2.03
**3**	1.78	0.30	0.25	0.13	0.14	0.10	2.07	2.03	1.91	1.92	1.88
**4**	1.71	0.32	0.27	0.15	0.16	0.12	2.02	1.98	1.86	1.87	1.83

7. DFT calculations

Geometry was optimized using density functional theory (DFT) with B3LYP function and the 6-31G(d,p) basis set. The excited state energy levels were calculated with time-dependent DFT (TD-DFT) level at the same basis set, solvent effects (chloroform) were incorporated via the SMD implicit solvation model. The spin density surfaces were calculated at the B3LYP/6-31G(d,p) level. All these calculations were performed with the Gaussian 09W program package.[8]

The spin-orbit coupling (SOC) matrix elements between S_n and T_n (n = 1, 2, 3, 4, 5) states were calculated with PySOC by considering that the three T_n substrates (m = 1, 0, -1) are degenerate, *i.e.*, $\langle S_1|\hat{H}_{soc}|T_1\rangle = \sqrt{\sum_{m=0,\pm1} \langle S_1|\hat{H}_{so}|T_1{}^m\rangle^2}$, where The $\hat{H}_{soc}$ represents the interaction of the SOC.[9] All SOCs were investigated at B3LYP/def2svp level.

Table S6. Theoretical calculation results of SOC matrix elements and ISC rate.

	S_1 [a]	T_1 [a]	$\Delta E(S_1\text{-}T_1)$ [a]	SOC(S_1-T_1)	$k_{ISC}\propto$
**1**	2.384	-	-	-	-
**2**	1.842	1.594	0.248	0.0658	0.070
**2Br**	1.771	1.581	0.190	23.908	15833
**3**	1.647	1.514	0.133	0.0177	0.018
**4**	1.560	1.512	0.048	0.0264	0.303

[a] The data are derived from fluorescence and phosphorescence emission peaks measured at low temperature.

The k_{ISC} can be calculated by the Equation (S8):

$$k_{ISC} \propto \frac{\langle T_1|H_{SO}|S_1\rangle}{(\Delta E_{S1-T1})^2} \qquad \text{(S8)}$$

Where the $\langle T_1|H_{SO}|S_1\rangle$ is the SOC matrix elements, ΔE_{S1-T1} is the adiabatic energy difference between S_1 and T_1.

Table S7. Cartesian coordinates of **1** at the optimized geometry.

Center Number	Atomic Number	Atomic Type	Coordinates (Angstroms) X	Y	Z
1	6	0	-3.684193	-0.001190	0.306020
2	6	0	-5.149732	0.000011	0.006770
3	6	0	-5.839696	1.213472	-0.127351
4	7	0	-7.208097	1.246295	-0.399662
5	5	0	-8.135049	0.002408	-0.605458
6	7	0	-7.208733	-1.242993	-0.406360
7	6	0	-5.840274	-1.212354	-0.134085
8	6	0	-7.583689	-2.529940	-0.477529
9	6	0	-6.478365	-3.376612	-0.255586
10	6	0	-5.381486	-2.552287	-0.039448
11	6	0	-5.380217	2.552640	-0.025419
12	6	0	-6.476618	3.378688	-0.237338
13	6	0	-7.582358	2.533797	-0.463994
14	9	0	-8.659027	0.006042	-1.890108
15	9	0	-9.148348	0.000124	0.341525
16	6	0	-3.243370	-0.007065	1.650381
17	6	0	-1.875891	-0.008087	1.911977
18	6	0	-0.939344	-0.003523	0.870579
19	6	0	-1.380728	0.002272	-0.455215
20	6	0	-2.751291	0.003526	-0.747952
21	6	0	-3.199970	0.009726	-2.192253
22	6	0	-4.225876	-0.012066	2.799491
23	8	0	0.367981	-0.005130	1.252677
24	6	0	15.486722	0.000957	-0.262408
25	6	0	14.137964	0.003261	-0.988262
26	6	0	12.936335	0.000315	-0.035445
27	6	0	11.581560	0.002592	-0.753890
28	6	0	10.380959	-0.000513	0.200180
29	6	0	9.025215	0.001841	-0.516786
30	6	0	7.826930	-0.001525	0.440174
31	6	0	6.469293	0.001037	-0.273207
32	6	0	5.275572	-0.002686	0.689126
33	6	0	3.915477	0.000170	-0.019913
34	6	0	2.733164	-0.003872	0.956627
35	6	0	1.386751	-0.000743	0.247540
36	1	0	-8.612300	-2.794613	-0.681229
37	1	0	-6.501528	-4.456853	-0.256400
38	1	0	-4.361681	-2.844805	0.166027
39	1	0	-4.360282	2.843494	0.181770

40	1	0	-6.499232	4.458930	-0.232350
41	1	0	-8.610796	2.800112	-0.666442
42	1	0	-1.510763	-0.012428	2.934072
43	1	0	-0.673396	0.005894	-1.275841
44	1	0	-2.341489	0.012919	-2.867758
45	1	0	-3.810615	-0.868867	-2.425280
46	1	0	-3.811111	0.889954	-2.417617
47	1	0	-4.878774	0.866642	2.771900
48	1	0	-4.876560	-0.892229	2.766194
49	1	0	-3.702669	-0.014547	3.758311
50	1	0	16.322732	0.003158	-0.969534
51	1	0	15.592374	0.882694	0.379997
52	1	0	15.592340	-0.884796	0.374456
53	1	0	14.076942	-0.871907	-1.649529
54	1	0	14.076996	0.882539	-1.644060
55	1	0	12.997841	0.875968	0.626769
56	1	0	12.997810	-0.879474	0.621271
57	1	0	11.521018	-0.873042	-1.416056
58	1	0	11.520975	0.882463	-1.410413
59	1	0	10.441897	0.874990	0.862405
60	1	0	10.441899	-0.880336	0.856658
61	1	0	8.963495	-0.873699	-1.178891
62	1	0	8.963374	0.881882	-1.172888
63	1	0	7.889433	0.873794	1.102418
64	1	0	7.889419	-0.881536	1.096176
65	1	0	6.405270	-0.874437	-0.935083
66	1	0	6.405128	0.881455	-0.928483
67	1	0	5.339536	0.872483	1.350908
68	1	0	5.339475	-0.883048	1.343992
69	1	0	3.849467	-0.875584	-0.680725
70	1	0	3.849414	0.881321	-0.673509
71	1	0	2.785028	0.873241	1.613278
72	1	0	2.784875	-0.886535	1.605813
73	1	0	1.281469	-0.886364	-0.394795
74	1	0	1.281502	0.890507	-0.386962

Table S8. Cartesian coordinates of **2** at the optimized geometry.

Center Number	Atomic Number	Atomic Type	Coordinates (Angstroms) X	Y	Z
1	6	0	3.845214	-3.184245	-0.225464

2	6	0	2.443404	-3.573247	0.121312
3	6	0	2.112102	-4.921713	0.318385
4	7	0	0.812174	-5.313460	0.642418
5	5	0	-0.393257	-4.355139	0.909426
6	7	0	0.134088	-2.920465	0.560563
7	6	0	1.451409	-2.587870	0.237583
8	6	0	-0.584113	-1.792295	0.567750
9	6	0	0.231246	-0.673142	0.251407
10	6	0	1.515221	-1.187782	0.046818
11	6	0	2.910869	-6.090915	0.220785
12	6	0	2.083582	-7.175379	0.484482
13	6	0	0.799734	-6.652681	0.740135
14	9	0	-0.759646	-4.409482	2.246318
15	9	0	-1.460947	-4.680005	0.088102
16	6	0	4.250658	-3.141469	-1.580280
17	6	0	5.558006	-2.769586	-1.881913
18	6	0	6.469052	-2.440096	-0.870254
19	6	0	6.063194	-2.486591	0.466196
20	6	0	4.753515	-2.856780	0.798948
21	6	0	4.343535	-2.897615	2.254366
22	6	0	3.293363	-3.487912	-2.697989
23	8	0	7.716624	-2.090708	-1.289667
24	6	0	22.138524	2.560048	-0.092509
25	6	0	20.859207	2.174030	0.656143
26	6	0	19.707229	1.777576	-0.275238
27	6	0	18.422596	1.388974	0.466714
28	6	0	17.271931	0.989899	-0.465124
29	6	0	15.986310	0.603278	0.276479
30	6	0	14.837676	0.201519	-0.656706
31	6	0	13.549946	-0.180890	0.083405
32	6	0	12.404767	-0.585336	-0.852557
33	6	0	11.114270	-0.960827	-0.113412
34	6	0	9.978080	-1.367215	-1.059778
35	6	0	8.700127	-1.726083	-0.315669
36	1	0	-1.640682	-1.813792	0.797473
37	1	0	2.409802	-0.643634	-0.220178
38	1	0	3.963633	-6.103211	-0.022624
39	1	0	2.351329	-8.222106	0.493473
40	1	0	-0.112174	-7.179468	0.986068
41	1	0	5.894239	-2.726363	-2.912950
42	1	0	6.751520	-2.237923	1.264770
43	1	0	5.181785	-2.634121	2.903488
44	1	0	3.525220	-2.199543	2.460055
45	1	0	3.991915	-3.893513	2.543522

46	1	0	2.924265	-4.514688	-2.605340
47	1	0	2.415916	-2.832684	-2.691387
48	1	0	3.780204	-3.390061	-3.670850
49	1	0	22.940298	2.836847	0.599948
50	1	0	22.502944	1.729780	-0.708290
51	1	0	21.965543	3.412907	-0.758804
52	1	0	20.539346	3.012278	1.290130
53	1	0	21.073030	1.341735	1.340599
54	1	0	20.027962	0.938996	-0.910078
55	1	0	19.493044	2.610266	-0.960616
56	1	0	18.101940	2.228556	1.100072
57	1	0	18.638218	0.557802	1.153362
58	1	0	17.592279	0.149339	-1.097287
59	1	0	17.057331	1.820348	-1.152854
60	1	0	15.664427	1.444698	0.906687
61	1	0	16.200801	-0.225885	0.965834
62	1	0	15.158728	-0.641823	-1.284714
63	1	0	14.625357	1.029464	-1.348068
64	1	0	13.226756	0.663587	0.708726
65	1	0	13.761628	-1.007448	0.776662
66	1	0	12.725270	-1.433134	-1.474268
67	1	0	12.195261	0.239063	-1.548479
68	1	0	10.791174	-0.111165	0.504362
69	1	0	11.322955	-1.783718	0.584626
70	1	0	10.284021	-2.227442	-1.667742
71	1	0	9.759328	-0.549775	-1.757646
72	1	0	8.340835	-0.872129	0.275406
73	1	0	8.871947	-2.565117	0.373256
74	6	0	-3.868912	3.198593	0.406846
75	6	0	-2.451629	3.593819	0.138925
76	6	0	-1.442933	2.618858	0.120536
77	7	0	-0.111743	2.958485	-0.130433
78	5	0	0.443098	4.406921	-0.360172
79	7	0	-0.813635	5.336096	-0.355112
80	6	0	-2.127071	4.936585	-0.102903
81	6	0	-0.821243	6.662883	-0.562432
82	6	0	-2.132041	7.169333	-0.452760
83	6	0	-2.954907	6.087957	-0.163951
84	6	0	-1.510232	1.217865	0.304197
85	6	0	-0.215811	0.708950	0.160135
86	6	0	0.610132	1.832977	-0.107611
87	9	0	1.089167	4.476866	-1.584264
88	9	0	1.299849	4.752619	0.674246
89	6	0	-4.361123	3.205478	1.733288

90	6	0	-5.682237	2.832206	1.964451
91	6	0	-6.522729	2.452612	0.910202
92	6	0	-6.030443	2.446626	-0.397725
93	6	0	-4.705054	2.818118	-0.659845
94	6	0	-4.201252	2.804886	-2.086001
95	6	0	-3.480711	3.606200	2.895159
96	8	0	-7.793314	2.111120	1.261663
97	6	0	-22.127553	-2.546653	-0.717045
98	6	0	-20.770951	-2.286092	-1.378651
99	6	0	-19.709477	-1.766112	-0.401410
100	6	0	-18.347845	-1.502126	-1.055432
101	6	0	-17.287658	-0.980419	-0.077727
102	6	0	-15.926726	-0.712841	-0.732120
103	6	0	-14.867014	-0.193234	0.247209
104	6	0	-13.507990	0.082278	-0.407839
105	6	0	-12.448345	0.594540	0.575123
106	6	0	-11.093359	0.883865	-0.082670
107	6	0	-10.036875	1.379251	0.912545
108	6	0	-8.705540	1.687591	0.243232
109	1	0	0.096189	7.193159	-0.778587
110	1	0	-2.420675	8.203254	-0.575640
111	1	0	-4.024904	6.090094	-0.013055
112	1	0	-2.417094	0.667275	0.509688
113	1	0	1.677826	1.860110	-0.277466
114	1	0	-6.084908	2.827653	2.972322
115	1	0	-6.662994	2.156531	-1.227914
116	1	0	-4.989266	2.493902	-2.775703
117	1	0	-3.852085	3.795386	-2.396133
118	1	0	-3.357020	2.117953	-2.206199
119	1	0	-2.597513	2.962804	2.968675
120	1	0	-3.117514	4.633576	2.786783
121	1	0	-4.027335	3.538449	3.838390
122	1	0	-22.863195	-2.912555	-1.440855
123	1	0	-22.042990	-3.295692	0.078589
124	1	0	-22.530515	-1.632236	-0.266676
125	1	0	-20.895429	-1.562058	-2.195490
126	1	0	-20.408484	-3.211298	-1.847083
127	1	0	-19.585341	-2.490293	0.416603
128	1	0	-20.072872	-0.840290	0.067470
129	1	0	-18.473378	-0.778912	-1.873986
130	1	0	-17.984817	-2.428349	-1.523514
131	1	0	-17.160561	-1.704560	0.739663
132	1	0	-17.651788	-0.055451	0.391918
133	1	0	-16.053563	0.012952	-1.548139

134	1	0	-15.562721	-1.637039	-1.203316
135	1	0	-14.736441	-0.921578	1.060233
136	1	0	-15.233199	0.728006	0.722441
137	1	0	-13.637629	0.815341	-1.216841
138	1	0	-13.143094	-0.837033	-0.887656
139	1	0	-12.310569	-0.143070	1.378119
140	1	0	-12.815723	1.508621	1.062377
141	1	0	-11.229644	1.631336	-0.876923
142	1	0	-10.729694	-0.026492	-0.579421
143	1	0	-9.871455	0.625030	1.691560
144	1	0	-10.392740	2.284201	1.420104
145	1	0	-8.822420	2.481662	-0.507520
146	1	0	-8.308271	0.798023	-0.265530

Table S9. Cartesian coordinates of **2Br** at the optimized geometry.

Center Number	Atomic Number	Atomic Type	Coordinates (Angstroms) X	Y	Z
1	6	0	3.887409	-2.970719	-0.087720
2	6	0	2.525249	-3.401379	0.352290
3	6	0	2.241923	-4.766399	0.541510
4	7	0	0.979255	-5.198002	0.947993
5	5	0	-0.215334	-4.271958	1.363074
6	7	0	0.234052	-2.825426	0.961466
7	6	0	1.518694	-2.449670	0.557069
8	6	0	-0.514946	-1.721476	1.027444
9	6	0	0.244746	-0.572811	0.671941
10	6	0	1.526882	-1.044182	0.379821
11	6	0	3.064338	-5.904315	0.345154
12	6	0	2.268196	-7.001379	0.637871
13	6	0	0.991873	-6.538800	1.006050
14	9	0	-0.410812	-4.340185	2.732977
15	9	0	-1.360033	-4.625811	0.671947
16	6	0	4.186968	-2.886650	-1.468452
17	6	0	5.459347	-2.476878	-1.857885
18	6	0	6.438462	-2.149965	-0.909828
19	6	0	6.135450	-2.237353	0.452386
20	6	0	4.863252	-2.646164	0.874289
21	6	0	4.565312	-2.728982	2.355818
22	6	0	3.155293	-3.229599	-2.520611
23	8	0	7.641455	-1.762436	-1.412637
24	6	0	22.073826	3.016718	-1.170272

25	6	0	20.851931	2.614460	-0.337200
26	6	0	19.643484	2.210503	-1.192365
27	6	0	18.414611	1.806422	-0.366986
28	6	0	17.206743	1.403198	-1.223365
29	6	0	15.977055	1.000103	-0.398391
30	6	0	14.770055	0.597122	-1.256081
31	6	0	13.539554	0.194894	-0.431898
32	6	0	12.334544	-0.208099	-1.292021
33	6	0	11.103754	-0.609319	-0.467506
34	6	0	9.905513	-1.011463	-1.337761
35	6	0	8.691404	-1.405731	-0.507794
36	1	0	-1.552245	-1.776762	1.330500
37	1	0	2.382038	-0.468388	0.053966
38	1	0	4.094971	-5.895645	0.022758
39	1	0	0.119253	-7.104270	1.300454
40	1	0	5.715411	-2.402611	-2.910474
41	1	0	6.877635	-1.990525	1.202708
42	1	0	5.448821	-2.471121	2.946490
43	1	0	3.757948	-2.045135	2.643012
44	1	0	4.247671	-3.736806	2.647209
45	1	0	2.816804	-4.268314	-2.429201
46	1	0	2.265495	-2.595103	-2.433631
47	1	0	3.566432	-3.097017	-3.525102
48	1	0	22.918477	3.301405	-0.532141
49	1	0	22.404365	2.190678	-1.812268
50	1	0	21.845289	3.869753	-1.821364
51	1	0	20.566780	3.447662	0.320989
52	1	0	21.119924	1.780402	0.327007
53	1	0	19.929152	1.376849	-1.851352
54	1	0	19.375954	3.045041	-1.857789
55	1	0	18.129997	2.640382	0.291887
56	1	0	18.682767	0.972012	0.298192
57	1	0	17.491203	0.568896	-1.881814
58	1	0	16.939190	2.237383	-1.888972
59	1	0	15.692166	1.834465	0.259773
60	1	0	16.244179	0.165827	0.267299
61	1	0	15.054734	-0.237625	-1.913785
62	1	0	14.503376	1.431142	-1.922146
63	1	0	13.253790	1.029793	0.225090
64	1	0	13.805557	-0.639211	0.234354
65	1	0	12.618886	-1.043806	-1.948098
66	1	0	12.067718	0.625312	-1.958227
67	1	0	10.817968	0.227051	0.187064
68	1	0	11.370113	-1.442863	0.198798

69	1	0	10.177301	-1.854536	-1.986049
70	1	0	9.625223	-0.181360	-1.998909
71	1	0	8.365020	-0.570947	0.128494
72	1	0	8.924638	-2.258794	0.145113
73	6	0	-4.007517	3.134266	0.754938
74	6	0	-2.592700	3.590534	0.599882
75	6	0	-1.546583	2.659892	0.616955
76	7	0	-0.215415	3.059761	0.464936
77	5	0	0.296842	4.540034	0.409600
78	7	0	-0.997673	5.412758	0.269442
79	6	0	-2.307524	4.955743	0.415690
80	6	0	-1.035179	6.739594	0.070679
81	6	0	-2.375108	7.167408	0.085947
82	6	0	-3.185456	6.062988	0.301688
83	6	0	-1.568861	1.247525	0.724177
84	6	0	-0.250738	0.794731	0.628674
85	6	0	0.545928	1.962370	0.470055
86	9	0	1.118520	4.720575	-0.688146
87	9	0	0.952023	4.854168	1.589313
88	6	0	-4.590311	3.084246	2.043754
89	6	0	-5.908917	2.655379	2.170597
90	6	0	-6.659919	2.276281	1.049599
91	6	0	-6.077246	2.329561	-0.220300
92	6	0	-4.752097	2.756748	-0.379050
93	6	0	-4.151765	2.803277	-1.767687
94	6	0	-3.808698	3.482961	3.276136
95	8	0	-7.935289	1.874144	1.299600
96	6	0	-21.668558	-3.796212	-1.979502
97	6	0	-20.297563	-3.370859	-2.516183
98	6	0	-19.363849	-2.823699	-1.428510
99	6	0	-17.989679	-2.393761	-1.958805
100	6	0	-17.053671	-1.849225	-0.871677
101	6	0	-15.680717	-1.419022	-1.405421
102	6	0	-14.740526	-0.878839	-0.319736
103	6	0	-13.368945	-0.449893	-0.857979
104	6	0	-12.422950	0.081935	0.226527
105	6	0	-11.053276	0.508227	-0.318985
106	6	0	-10.103516	1.022968	0.770707
107	6	0	-8.751787	1.439733	0.207440
108	1	0	-0.134553	7.320240	-0.069721
109	1	0	-4.263072	6.028420	0.363324
110	1	0	-2.464173	0.652009	0.837871
111	1	0	1.621062	2.036959	0.373005
112	1	0	-6.379629	2.605316	3.147833

113	1	0	-6.639429	2.042698	-1.101301
114	1	0	-4.885410	2.500259	-2.519983
115	1	0	-3.804711	3.811159	-2.023315
116	1	0	-3.286209	2.136171	-1.854880
117	1	0	-2.898880	2.881610	3.387419
118	1	0	-3.493904	4.532214	3.231352
119	1	0	-4.412222	3.351353	4.178397
120	1	0	-22.310223	-4.181756	-2.780340
121	1	0	-21.571025	-4.584186	-1.222229
122	1	0	-22.190150	-2.951778	-1.511908
123	1	0	-20.431065	-2.607305	-3.295975
124	1	0	-19.815604	-4.227309	-3.009133
125	1	0	-19.230052	-3.588492	-0.648643
126	1	0	-19.847039	-1.967733	-0.933832
127	1	0	-18.124941	-1.628168	-2.737520
128	1	0	-17.508802	-3.249729	-2.455521
129	1	0	-16.916953	-2.615226	-0.093728
130	1	0	-17.534101	-0.993678	-0.373939
131	1	0	-15.817637	-0.651026	-2.181424
132	1	0	-15.202311	-2.274022	-1.906074
133	1	0	-14.602088	-1.647178	0.455516
134	1	0	-15.217601	-0.023768	0.181884
135	1	0	-13.507160	0.322272	-1.629525
136	1	0	-12.895116	-1.303935	-1.364433
137	1	0	-12.281600	-0.690440	0.996757
138	1	0	-12.893394	0.936578	0.734333
139	1	0	-11.194077	1.288148	-1.081778
140	1	0	-10.588701	-0.344540	-0.835351
141	1	0	-9.943195	0.244930	1.528251
142	1	0	-10.551480	1.881276	1.287880
143	1	0	-8.865857	2.257014	-0.518901
144	1	0	-8.266445	0.596797	-0.304652
145	35	0	2.780036	-8.868775	0.555255
146	35	0	-2.948353	9.001448	-0.167283

Table S10. Cartesian coordinates of **3** at the optimized geometry.

Center Number	Atomic Number	Atomic Type	Coordinates (Angstroms) X	Y	Z
1	6	0	5.005535	-0.189485	-0.435902
2	6	0	3.544785	-0.125363	-0.122876
3	6	0	2.796296	-1.306255	-0.005870

4	7	0	1.428536	-1.274364	0.276585
5	5	0	0.584724	0.007526	0.587867
6	7	0	1.551793	1.210674	0.326004
7	6	0	2.915303	1.118221	0.037519
8	6	0	1.233052	2.508338	0.393096
9	6	0	2.372886	3.320465	0.152316
10	6	0	3.429279	2.431412	-0.069881
11	6	0	3.180377	-2.659237	-0.155523
12	6	0	2.040725	-3.447445	0.033784
13	6	0	0.984416	-2.536129	0.298694
14	9	0	0.187039	0.000341	1.916183
15	9	0	-0.508201	0.079616	-0.259719
16	6	0	5.434824	-0.203251	-1.784105
17	6	0	6.798895	-0.256822	-2.056825
18	6	0	7.743254	-0.297192	-1.023071
19	6	0	7.312912	-0.285690	0.306501
20	6	0	5.946162	-0.231085	0.610619
21	6	0	5.509262	-0.216805	2.058493
22	6	0	4.444023	-0.157570	-2.925199
23	8	0	9.045821	-0.345728	-1.415732
24	6	0	24.175099	-0.464833	-0.009069
25	6	0	22.831082	-0.461163	0.725542
26	6	0	21.623279	-0.460307	-0.219461
27	6	0	20.273254	-0.456039	0.507875
28	6	0	19.066315	-0.454486	-0.438181
29	6	0	17.715496	-0.448278	0.288015
30	6	0	16.510586	-0.444705	-0.660599
31	6	0	15.158127	-0.434628	0.062501
32	6	0	13.957431	-0.426916	-0.891101
33	6	0	12.602879	-0.410349	-0.171710
34	6	0	11.413162	-0.395634	-1.139169
35	6	0	10.073000	-0.368812	-0.418932
36	1	0	0.219104	2.815560	0.610407
37	1	0	4.458272	2.672345	-0.294970
38	1	0	4.182933	-2.992009	-0.383144
39	1	0	-0.056014	-2.750179	0.501719
40	1	0	7.155602	-0.266412	-3.081816
41	1	0	8.026250	-0.318781	1.121190
42	1	0	6.371065	-0.278352	2.726899
43	1	0	4.958743	0.698264	2.301345
44	1	0	4.845081	-1.057386	2.285147
45	1	0	3.760052	-1.012282	-2.896431
46	1	0	3.826730	0.746114	-2.883813
47	1	0	4.959632	-0.170134	-3.887991

48	1	0	25.015742	-0.465427	0.692567
49	1	0	24.274814	-1.349701	-0.648119
50	1	0	24.278220	0.417787	-0.650683
51	1	0	22.775991	0.417198	1.383079
52	1	0	22.772682	-1.337252	1.385818
53	1	0	21.678599	-1.339307	-0.877774
54	1	0	21.682330	0.416151	-0.880839
55	1	0	20.219154	0.422896	1.166211
56	1	0	20.214948	-1.332609	1.168998
57	1	0	19.120021	-1.333799	-1.095963
58	1	0	19.125608	0.421550	-1.099858
59	1	0	17.661713	0.430802	0.946108
60	1	0	17.654793	-1.324776	0.948938
61	1	0	16.563571	-1.324563	-1.317622
62	1	0	16.573276	0.430791	-1.322602
63	1	0	15.104994	0.444693	0.720221
64	1	0	15.092600	-1.311186	0.722778
65	1	0	14.007873	-1.307517	-1.546784
66	1	0	14.024977	0.448024	-1.552842
67	1	0	12.552723	0.469465	0.485076
68	1	0	12.531028	-1.287397	0.486733
69	1	0	11.445315	-1.278813	-1.788848
70	1	0	11.474112	0.480908	-1.795799
71	1	0	9.991921	0.521292	0.220517
72	1	0	9.953719	-1.255455	0.219438
73	6	0	-0.259819	8.294237	0.549498
74	6	0	1.213628	8.224673	0.303212
75	6	0	1.852634	6.978521	0.217366
76	7	0	3.226642	6.883445	-0.013883
77	5	0	4.223027	8.085158	-0.162284
78	7	0	3.337033	9.369674	-0.075520
79	6	0	1.960236	9.402175	0.152231
80	6	0	3.764130	10.637819	-0.187858
81	6	0	2.686529	11.533897	-0.037267
82	6	0	1.551934	10.761400	0.176380
83	6	0	1.333606	5.666457	0.316214
84	6	0	2.397041	4.775569	0.141160
85	6	0	3.546519	5.585716	-0.058771
86	9	0	4.860087	8.022131	-1.391571
87	9	0	5.142615	8.064631	0.875317
88	6	0	-0.746228	8.406168	1.873427
89	6	0	-2.121012	8.467715	2.084329
90	6	0	-3.021018	8.419995	1.012110
91	6	0	-2.534445	8.308578	-0.293173

92	6	0	-1.155718	8.244898	-0.535187
93	6	0	-0.659594	8.126673	-1.959198
94	6	0	0.196131	8.452060	3.054791
95	8	0	-4.339983	8.489224	1.344333
96	6	0	-19.365375	8.505235	-1.005179
97	6	0	-17.974320	8.364952	-1.630654
98	6	0	-16.835099	8.505023	-0.613415
99	6	0	-15.438616	8.368400	-1.231935
100	6	0	-14.298988	8.507454	-0.215044
101	6	0	-12.903241	8.376699	-0.836899
102	6	0	-11.762125	8.509720	0.179107
103	6	0	-10.367787	8.388402	-0.447816
104	6	0	-9.225342	8.509136	0.567918
105	6	0	-7.833193	8.400922	-0.066638
106	6	0	-6.694847	8.501430	0.956095
107	6	0	-5.321955	8.410298	0.306049
108	1	0	4.807951	10.854820	-0.369473
109	1	0	2.751358	12.611378	-0.084417
110	1	0	0.537882	11.100934	0.331677
111	1	0	0.294336	5.427836	0.491291
112	1	0	4.569191	5.276133	-0.226120
113	1	0	-2.520548	8.550846	3.090026
114	1	0	-3.212403	8.270247	-1.137130
115	1	0	-1.492345	8.150945	-2.665794
116	1	0	0.024410	8.942830	-2.214000
117	1	0	-0.111436	7.191681	-2.115983
118	1	0	0.830449	7.560211	3.094562
119	1	0	0.866116	9.316496	3.000610
120	1	0	-0.359558	8.513430	3.993123
121	1	0	-20.155466	8.401067	-1.756084
122	1	0	-19.533380	7.741187	-0.237531
123	1	0	-19.487710	9.484072	-0.527411
124	1	0	-17.849534	9.120164	-2.418684
125	1	0	-17.894302	7.390027	-2.130631
126	1	0	-16.959312	7.748740	0.175001
127	1	0	-16.916655	9.480138	-0.111858
128	1	0	-15.315763	9.125528	-2.019638
129	1	0	-15.358453	7.393855	-1.734615
130	1	0	-14.419005	7.747639	0.570407
131	1	0	-14.380964	9.480279	0.290541
132	1	0	-12.782559	9.139453	-1.619448
133	1	0	-12.822709	7.405797	-1.346433
134	1	0	-11.878183	7.742271	0.957622
135	1	0	-11.845352	9.477628	0.693728

136	1	0	-10.249624	9.161500	-1.220484
137	1	0	-10.287335	7.424175	-0.969758
138	1	0	-9.335925	7.729116	1.334188
139	1	0	-9.308261	9.468757	1.097224
140	1	0	-7.718700	9.190376	-0.822784
141	1	0	-7.755062	7.447277	-0.607371
142	1	0	-6.785259	7.699779	1.699197
143	1	0	-6.762652	9.449145	1.504226
144	1	0	-5.175644	9.230847	-0.410370
145	1	0	-5.210661	7.462674	-0.239450
146	6	0	-1.051657	-8.153577	0.390083
147	6	0	0.411789	-8.218233	0.088970
148	6	0	1.036078	-9.458361	-0.107766
149	7	0	2.400784	-9.552580	-0.384671
150	5	0	3.399328	-8.356133	-0.503194
151	7	0	2.535586	-7.069501	-0.262967
152	6	0	1.165587	-7.037643	0.007182
153	6	0	2.979524	-5.808101	-0.281237
154	6	0	1.921069	-4.896749	-0.023826
155	6	0	0.780200	-5.684933	0.156537
156	6	0	0.503285	-10.773751	-0.076752
157	6	0	1.552132	-11.646796	-0.336318
158	6	0	2.703099	-10.853926	-0.519579
159	9	0	3.957990	-8.319142	-1.771383
160	9	0	4.382330	-8.453675	0.469555
161	6	0	-1.491390	-8.170707	1.734793
162	6	0	-2.857126	-8.113890	1.998685
163	6	0	-3.793075	-8.039884	0.959261
164	6	0	-3.352542	-8.020525	-0.366756
165	6	0	-1.983764	-8.077619	-0.661936
166	6	0	-1.536661	-8.057071	-2.106649
167	6	0	-0.509235	-8.251583	2.881332
168	8	0	-5.098669	-7.991633	1.343140
169	6	0	-20.215563	-7.752871	-0.166146
170	6	0	-18.867029	-7.753200	-0.892424
171	6	0	-17.665164	-7.768703	0.059978
172	6	0	-16.310574	-7.769522	-0.658820
173	6	0	-15.109826	-7.787754	0.294900
174	6	0	-13.754209	-7.790184	-0.422302
175	6	0	-12.555933	-7.813946	0.534391
176	6	0	-11.198381	-7.819793	-0.179118
177	6	0	-10.004866	-7.852590	0.782947
178	6	0	-8.644963	-7.864398	0.073668
179	6	0	-7.462979	-7.910123	1.049622

180	6	0	-6.117303	-7.931083	0.339491
181	1	0	4.021852	-5.593518	-0.473682
182	1	0	-0.225121	-5.351936	0.371316
183	1	0	-0.531273	-11.018459	0.116910
184	1	0	3.715451	-11.165500	-0.737800
185	1	0	-3.221438	-8.127015	3.020975
186	1	0	-4.059233	-7.960296	-1.185692
187	1	0	-2.393017	-7.966261	-2.778768
188	1	0	-0.860907	-7.218714	-2.305411
189	1	0	-0.995288	-8.971533	-2.371200
190	1	0	0.104869	-9.156221	2.819563
191	1	0	0.178621	-7.399538	2.879854
192	1	0	-1.031500	-8.262301	3.840561
193	1	0	-21.051787	-7.741658	-0.872939
194	1	0	-20.325746	-8.642092	0.465091
195	1	0	-20.316241	-6.874710	0.481930
196	1	0	-18.801557	-6.870137	-1.542685
197	1	0	-18.810861	-8.624493	-1.559208
198	1	0	-17.731341	-8.652323	0.711084
199	1	0	-17.721649	-6.896982	0.727787
200	1	0	-16.244517	-6.885146	-1.308733
201	1	0	-16.255761	-8.640532	-1.327549
202	1	0	-15.176922	-8.671920	0.944934
203	1	0	-15.164419	-6.916731	0.963527
204	1	0	-13.684619	-6.904458	-1.069920
205	1	0	-13.700315	-8.659847	-1.092788
206	1	0	-12.627581	-8.699150	1.182458
207	1	0	-12.609267	-6.944037	1.204472
208	1	0	-11.122426	-6.932029	-0.823103
209	1	0	-11.145998	-8.687567	-0.852080
210	1	0	-10.082894	-8.739325	1.427663
211	1	0	-10.054804	-6.984177	1.454677
212	1	0	-8.560985	-6.973834	-0.564884
213	1	0	-8.596630	-8.730076	-0.601815
214	1	0	-7.535736	-8.801016	1.685455
215	1	0	-7.493704	-7.041948	1.719229
216	1	0	-5.984004	-7.028923	-0.273878
217	1	0	-6.040237	-8.804338	-0.323446
218	1	0	1.514472	-12.725239	-0.390565

Table S11. Cartesian coordinates of **4** at the optimized geometry.

Center Number	Atomic Number	Atomic Type	Coordinates (Angstroms) X	Y	Z
1	6	0	0.052303	5.071398	-0.155729
2	6	0	1.251366	4.220005	-0.425221
3	6	0	1.100182	2.838228	-0.621014
4	7	0	2.200871	2.016099	-0.872653
5	5	0	3.670190	2.503618	-1.103417
6	7	0	3.667329	4.015195	-0.696372
7	6	0	2.531970	4.792642	-0.455229
8	6	0	4.737386	4.812838	-0.612874
9	6	0	4.350166	6.146512	-0.315840
10	6	0	2.954962	6.122723	-0.223088
11	6	0	-0.051891	2.019959	-0.562260
12	6	0	0.355033	0.698477	-0.773785
13	6	0	1.761353	0.755844	-0.961316
14	9	0	4.005343	2.377108	-2.443270
15	9	0	4.543890	1.791619	-0.299532
16	6	0	-0.351333	5.320233	1.177458
17	6	0	-1.478081	6.105550	1.405309
18	6	0	-2.209740	6.648668	0.341502
19	6	0	-1.802740	6.403856	-0.972885
20	6	0	-0.674230	5.614978	-1.232136
21	6	0	-0.259211	5.364114	-2.664929
22	6	0	0.411587	4.747053	2.350306
23	8	0	-3.294163	7.393955	0.691017
24	6	0	-16.087962	15.359407	-1.231419
25	6	0	-14.891166	14.696722	-1.920174
26	6	0	-13.916952	14.035362	-0.937551
27	6	0	-12.716982	13.364976	-1.617227
28	6	0	-11.742341	12.708443	-0.631807
29	6	0	-10.545047	12.030838	-1.309409
30	6	0	-9.572002	11.377152	-0.320540
31	6	0	-8.379254	10.688419	-0.994960
32	6	0	-7.410745	10.036192	-0.000991
33	6	0	-6.225748	9.330907	-0.672397
34	6	0	-5.268819	8.681344	0.334693
35	6	0	-4.111520	7.959071	-0.339481
36	1	0	5.731858	4.418225	-0.770286
37	1	0	2.294602	6.948454	0.000151
38	1	0	-1.052871	2.379947	-0.371946
39	1	0	2.450316	-0.055233	-1.153559
40	1	0	-1.812383	6.308349	2.417731
41	1	0	-2.350636	6.818982	-1.810122

42	1	0	-0.902540	5.909690	-3.359097
43	1	0	0.774361	5.678141	-2.843673
44	1	0	-0.316716	4.300097	-2.918237
45	1	0	0.472137	3.655123	2.293798
46	1	0	1.440033	5.122178	2.381380
47	1	0	-0.071664	5.010301	3.293811
48	1	0	-16.761877	15.825300	-1.957866
49	1	0	-16.670069	14.628097	-0.659074
50	1	0	-15.761283	16.138797	-0.533349
51	1	0	-14.350947	15.445170	-2.515934
52	1	0	-15.250898	13.943717	-2.634631
53	1	0	-14.458606	13.289034	-0.338672
54	1	0	-13.555737	14.790236	-0.224179
55	1	0	-12.177476	14.110645	-2.218631
56	1	0	-13.078954	12.608069	-2.327867
57	1	0	-12.283304	11.966539	-0.027101
58	1	0	-11.377323	13.466591	0.075868
59	1	0	-10.003891	12.771321	-1.915626
60	1	0	-10.910007	11.270777	-2.015075
61	1	0	-10.115523	10.642198	0.290247
62	1	0	-9.202180	12.138633	0.380943
63	1	0	-7.834041	11.421558	-1.606326
64	1	0	-8.749189	9.925434	-1.694766
65	1	0	-7.958496	9.310694	0.616723
66	1	0	-7.032137	10.800275	0.692419
67	1	0	-5.674935	10.054252	-1.289784
68	1	0	-6.605284	8.565476	-1.363899
69	1	0	-5.812630	7.961556	0.958705
70	1	0	-4.862119	9.441925	1.012304
71	1	0	-3.516812	8.654274	-0.948599
72	1	0	-4.481374	7.162566	-1.000135
73	6	0	9.567273	8.247031	0.002616
74	6	0	8.377078	9.138506	0.161056
75	6	0	7.084290	8.601551	0.064403
76	7	0	5.951557	9.404567	0.213874
77	5	0	5.946131	10.954510	0.451085
78	7	0	7.451183	11.359176	0.573845
79	6	0	8.545349	10.506890	0.415975
80	6	0	7.923914	12.591626	0.820104
81	6	0	9.333501	12.583942	0.830284
82	6	0	9.727355	11.276281	0.576219
83	6	0	6.653063	7.273296	-0.158306
84	6	0	5.254884	7.272900	-0.140734
85	6	0	4.874162	8.620921	0.093869

86	9	0	5.270463	11.250344	1.624443
87	9	0	5.364484	11.595053	-0.632590
88	6	0	10.124606	8.039474	-1.281057
89	6	0	11.225084	7.196431	-1.410785
90	6	0	11.781731	6.555850	-0.296473
91	6	0	11.229149	6.768174	0.969460
92	6	0	10.121246	7.610888	1.129617
93	6	0	9.542606	7.816712	2.511918
94	6	0	9.546290	8.711438	-2.505716
95	8	0	12.850904	5.751087	-0.549574
96	6	0	23.626363	-4.526137	2.508284
97	6	0	22.607015	-3.540297	3.087241
98	6	0	21.830542	-2.770352	2.012084
99	6	0	20.803294	-1.784674	2.582347
100	6	0	20.030397	-1.012910	1.505936
101	6	0	18.991438	-0.038606	2.074933
102	6	0	18.225154	0.738073	0.997310
103	6	0	17.164243	1.689722	1.564068
104	6	0	16.409454	2.475783	0.485348
105	6	0	15.315821	3.391123	1.049937
106	6	0	14.579523	4.187813	-0.034071
107	6	0	13.458413	5.043951	0.536494
108	1	0	7.247880	13.421028	0.977253
109	1	0	9.967213	13.441316	1.005536
110	1	0	10.733333	10.887309	0.509600
111	1	0	7.311307	6.428792	-0.303955
112	1	0	3.878841	9.036158	0.174939
113	1	0	11.670464	7.018316	-2.384386
114	1	0	11.645960	6.285624	1.845102
115	1	0	10.104510	7.250913	3.258609
116	1	0	9.563091	8.872328	2.802050
117	1	0	8.497332	7.493673	2.560678
118	1	0	8.505019	8.414924	-2.671112
119	1	0	9.554299	9.801855	-2.405723
120	1	0	10.117403	8.449207	-3.399089
121	1	0	24.163928	-5.058538	3.299827
122	1	0	23.137061	-5.276063	1.876271
123	1	0	24.370075	-4.009796	1.890619
124	1	0	23.121537	-2.824918	3.743345
125	1	0	21.897137	-4.081529	3.727636
126	1	0	21.319055	-3.486691	1.353085
127	1	0	22.541054	-2.226376	1.373141
128	1	0	21.314123	-1.070624	3.244160
129	1	0	20.091699	-2.330518	3.218344

130	1	0	19.528076	-1.727395	0.838168
131	1	0	20.741324	-0.459180	0.876155
132	1	0	19.490670	0.672461	2.748634
133	1	0	18.276676	-0.594854	2.698169
134	1	0	17.742343	0.026687	0.312132
135	1	0	18.938167	1.309862	0.386414
136	1	0	17.641662	2.394668	2.259582
137	1	0	16.444741	1.113507	2.163141
138	1	0	15.956932	1.772268	-0.227582
139	1	0	17.124289	3.077075	-0.093681
140	1	0	15.761460	4.085424	1.776082
141	1	0	14.593221	2.783466	1.612635
142	1	0	14.153229	3.504765	-0.779066
143	1	0	15.283794	4.837387	-0.567987
144	1	0	13.847872	5.758853	1.275119
145	1	0	12.709001	4.415794	1.037945
146	6	0	-9.337779	-8.356696	0.705544
147	6	0	-8.198333	-9.208331	0.243807
148	6	0	-8.350758	-10.597637	0.132780
149	7	0	-7.302019	-11.414676	-0.292780
150	5	0	-5.882415	-10.932001	-0.735039
151	7	0	-5.881764	-9.380855	-0.504941
152	6	0	-6.966541	-8.615113	-0.072279
153	6	0	-4.851903	-8.552876	-0.712764
154	6	0	-5.218004	-7.211030	-0.425913
155	6	0	-6.555748	-7.262800	-0.022475
156	6	0	-9.470463	-11.426955	0.403838
157	6	0	-9.084453	-12.735133	0.140756
158	6	0	-7.742082	-12.683200	-0.286406
159	9	0	-5.679761	-11.209815	-2.078476
160	9	0	-4.905363	-11.526257	0.047975
161	6	0	-9.527897	-8.127319	2.088707
162	6	0	-10.583996	-7.318644	2.499726
163	6	0	-11.452276	-6.733223	1.569479
164	6	0	-11.262214	-6.967498	0.204878
165	6	0	-10.207678	-7.777205	-0.237390
166	6	0	-10.026413	-8.011689	-1.720631
167	6	0	-8.605481	-8.737154	3.119717
168	8	0	-12.442667	-5.958076	2.091766
169	6	0	-24.041998	3.864441	2.026055
170	6	0	-23.080469	3.024501	1.179979
171	6	0	-22.112255	2.182145	2.019659
172	6	0	-21.146275	1.336536	1.181056
173	6	0	-20.174028	0.500073	2.021932

174	6	0	-19.207358	-0.345367	1.183659
175	6	0	-18.228365	-1.172864	2.025587
176	6	0	-17.259417	-2.016159	1.187773
177	6	0	-16.270895	-2.830026	2.031538
178	6	0	-15.298599	-3.669040	1.192996
179	6	0	-14.300281	-4.461373	2.046081
180	6	0	-13.337812	-5.283535	1.201524
181	1	0	-3.900768	-8.932651	-1.060303
182	1	0	-7.183455	-6.439716	0.287449
183	1	0	-10.431380	-11.076039	0.752095
184	1	0	-9.680417	-13.630985	0.239045
185	1	0	-7.091565	-13.494426	-0.583624
186	1	0	-10.749634	-7.122934	3.554329
187	1	0	-11.924455	-6.528392	-0.531392
188	1	0	-10.820393	-7.525975	-2.292587
189	1	0	-9.067479	-7.618334	-2.074391
190	1	0	-10.040371	-9.079671	-1.961677
191	1	0	-8.635858	-9.831338	3.086885
192	1	0	-7.564676	-8.442588	2.949084
193	1	0	-8.885477	-8.421352	4.127162
194	1	0	-24.714509	4.458537	1.398593
195	1	0	-24.661905	3.229460	2.669313
196	1	0	-23.495105	4.557016	2.676211
197	1	0	-22.503798	3.684481	0.517515
198	1	0	-23.656943	2.362092	0.519912
199	1	0	-22.689402	1.523098	2.684223
200	1	0	-21.534947	2.845702	2.679602
201	1	0	-20.572873	1.995907	0.513747
202	1	0	-21.724101	0.670476	0.524293
203	1	0	-20.746736	-0.159008	2.690038
204	1	0	-19.596248	1.166900	2.677875
205	1	0	-18.639146	0.313326	0.511338
206	1	0	-19.784361	-1.017212	0.532163
207	1	0	-18.795307	-1.832273	2.698176
208	1	0	-17.652251	-0.499984	2.676700
209	1	0	-16.698912	-1.357326	0.509265
210	1	0	-17.834094	-2.696513	0.543191
211	1	0	-16.828540	-3.491194	2.709614
212	1	0	-15.696946	-2.149050	2.675547
213	1	0	-14.749578	-3.008379	0.507439
214	1	0	-15.870673	-4.360164	0.558138
215	1	0	-14.836234	-5.134270	2.726648
216	1	0	-13.717114	-3.776594	2.673705
217	1	0	-12.767050	-4.636705	0.520725

218	1	0	-13.882948	-6.017580	0.591534
219	6	0	-0.135700	-4.897591	-1.271837
220	6	0	-1.334300	-4.046207	-0.999534
221	6	0	-2.593612	-4.637397	-0.817795
222	7	0	-3.727657	-3.863445	-0.560040
223	5	0	-3.788912	-2.299062	-0.558327
224	7	0	-2.298736	-1.829839	-0.657351
225	6	0	-1.197975	-2.652227	-0.908506
226	6	0	-1.861511	-0.567587	-0.587565
227	6	0	-0.457570	-0.508557	-0.791536
228	6	0	-0.049799	-1.830882	-0.995398
229	6	0	-2.982136	-5.997829	-0.809358
230	6	0	-4.353882	-6.045599	-0.541785
231	6	0	-4.763463	-4.693642	-0.396759
232	9	0	-4.357338	-1.842129	0.618585
233	9	0	-4.499572	-1.853998	-1.662919
234	6	0	0.219791	-5.201242	-2.607272
235	6	0	1.345542	-5.985212	-2.844099
236	6	0	2.124108	-6.472506	-1.786562
237	6	0	1.764837	-6.173320	-0.469504
238	6	0	0.637067	-5.386184	-0.201402
239	6	0	0.274078	-5.078515	1.234481
240	6	0	-0.597838	-4.691266	-3.772254
241	8	0	3.203443	-7.221553	-2.144021
242	6	0	16.073843	-15.076160	-0.240071
243	6	0	14.879513	-14.408110	0.447777
244	6	0	13.894001	-13.767429	-0.537372
245	6	0	12.697257	-13.090691	0.141675
246	6	0	11.709775	-12.456536	-0.845705
247	6	0	10.517838	-11.769917	-0.167749
248	6	0	9.528537	-11.142385	-1.157532
249	6	0	8.344738	-10.440323	-0.481096
250	6	0	7.354372	-9.820239	-1.474219
251	6	0	6.183355	-9.096024	-0.798444
252	6	0	5.196594	-8.486471	-1.801918
253	6	0	4.059277	-7.739792	-1.120288
254	1	0	-2.550309	0.244221	-0.397757
255	1	0	0.952336	-2.191545	-1.178075
256	1	0	-2.312076	-6.830430	-0.969287
257	1	0	-5.752595	-4.307053	-0.192307
258	1	0	1.641826	-6.231170	-3.858786
259	1	0	2.350216	-6.543679	0.363294
260	1	0	0.991983	-5.529019	1.923720
261	1	0	0.257822	-3.999717	1.421527

262	1	0	-0.720674	-5.460861	1.486554
263	1	0	-1.629892	-5.054989	-3.726499
264	1	0	-0.646133	-3.597277	-3.780092
265	1	0	-0.167155	-5.017068	-4.721664
266	1	0	16.755977	-15.527105	0.488136
267	1	0	15.745212	-15.868187	-0.922833
268	1	0	16.647614	-14.351393	-0.828905
269	1	0	15.241318	-13.641853	1.146921
270	1	0	14.347766	-15.149315	1.059971
271	1	0	13.529862	-14.535693	-1.234762
272	1	0	14.427218	-13.029018	-1.153352
273	1	0	13.062317	-12.319483	0.835155
274	1	0	12.167153	-13.827987	0.761462
275	1	0	11.339736	-13.229460	-1.534507
276	1	0	12.241461	-11.724644	-1.470461
277	1	0	10.888159	-10.993054	0.516507
278	1	0	9.988094	-12.499495	0.461320
279	1	0	9.150130	-11.921440	-1.834679
280	1	0	10.060751	-10.421080	-1.793952
281	1	0	8.723773	-9.656596	0.190346
282	1	0	7.814299	-11.158452	0.160323
283	1	0	6.962277	-10.605957	-2.135135
284	1	0	7.887266	-9.113499	-2.125683
285	1	0	6.577108	-8.305044	-0.144665
286	1	0	5.652090	-9.798938	-0.141613
287	1	0	4.770969	-9.273146	-2.436664
288	1	0	5.720495	-7.791300	-2.469317
289	1	0	4.448342	-6.914929	-0.507131
290	1	0	3.489502	-8.410522	-0.461883

----

8. ^{1}H and ^{13}C NMR spectra for all new compounds

^{1}H NMR (400 MHz, $CDCl_3$) for **BDP-OH**

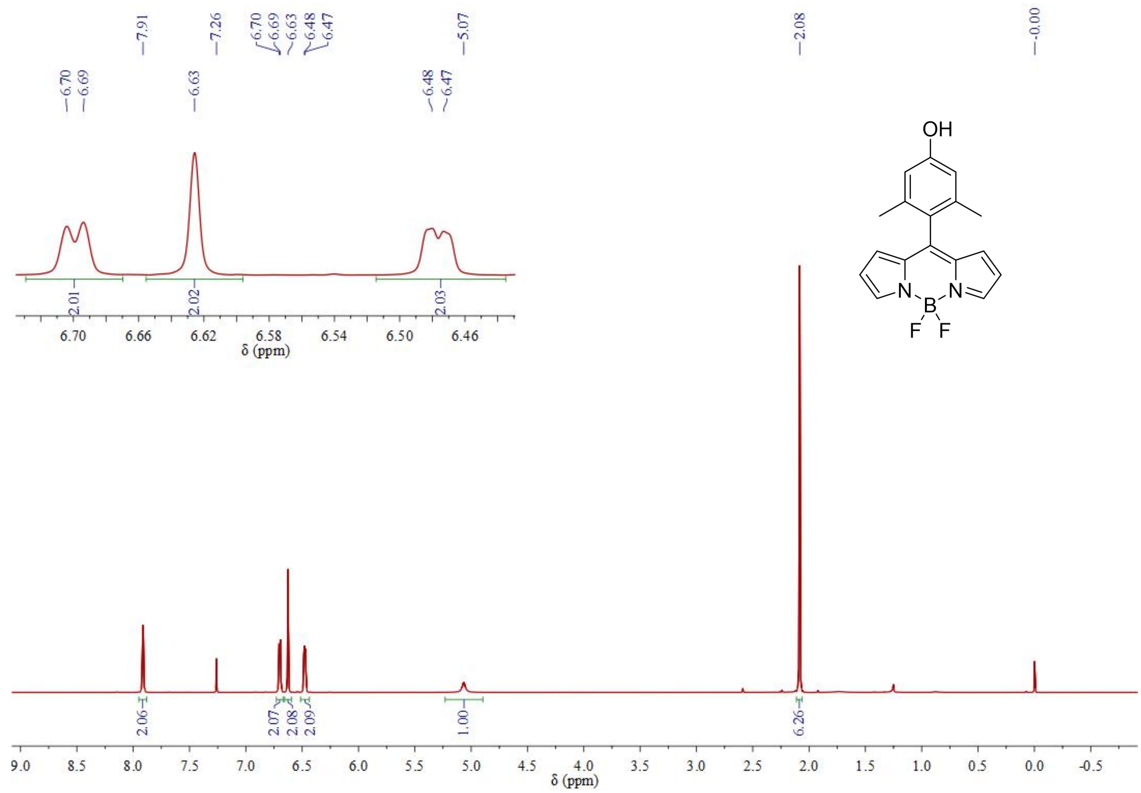

^{13}C NMR (100 MHz, $CDCl_3$) for **BDP-OH**

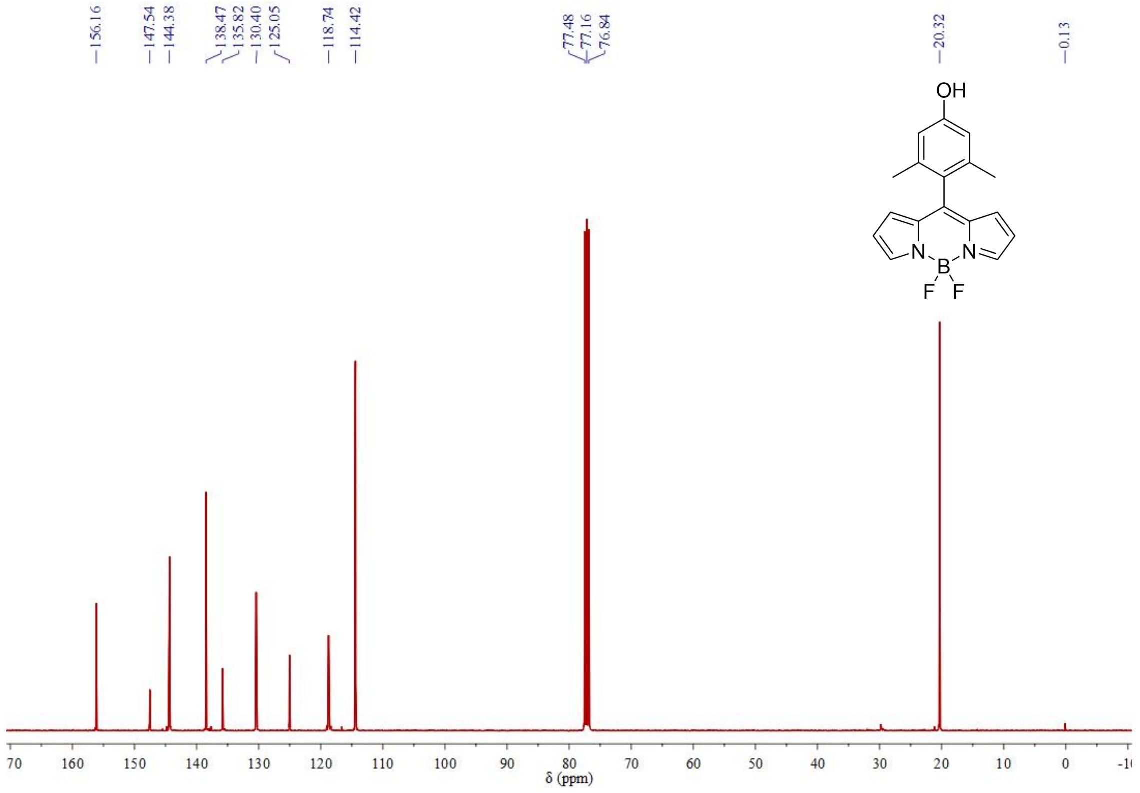

^{1}H NMR (400 MHz, $CDCl_3$) for **1**

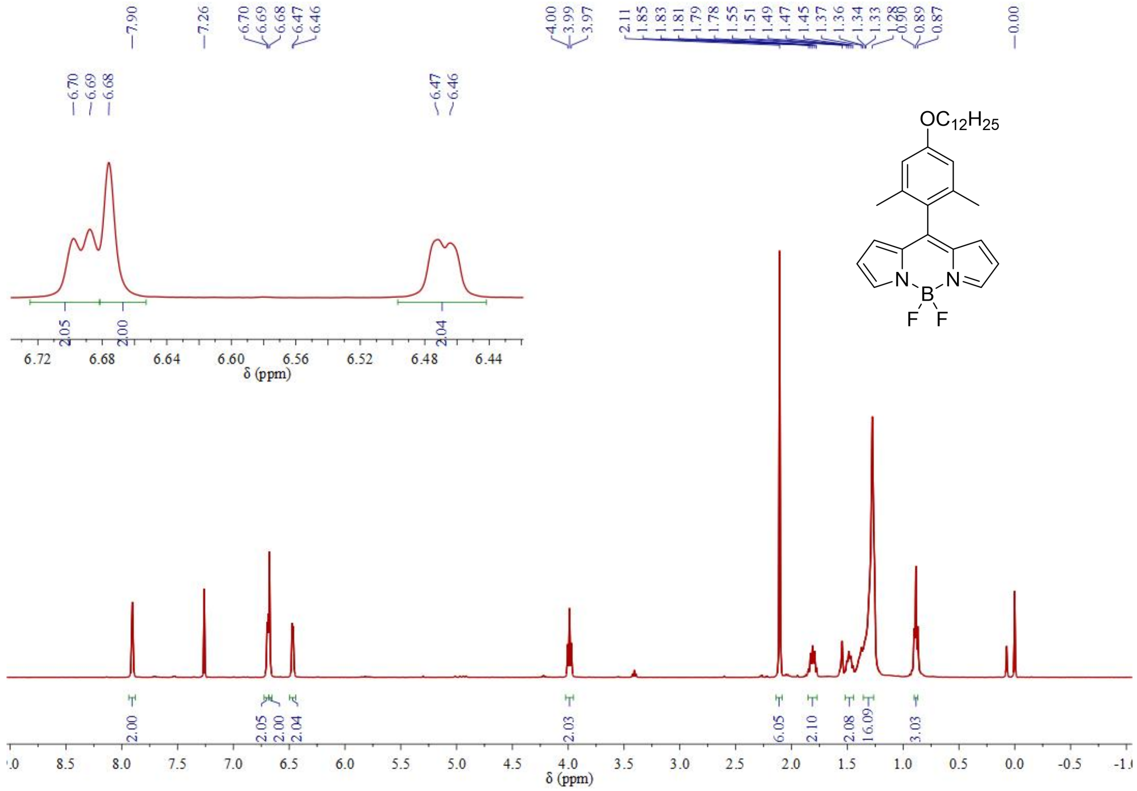

^{13}C NMR (100 MHz, $CDCl_3$) for **1**

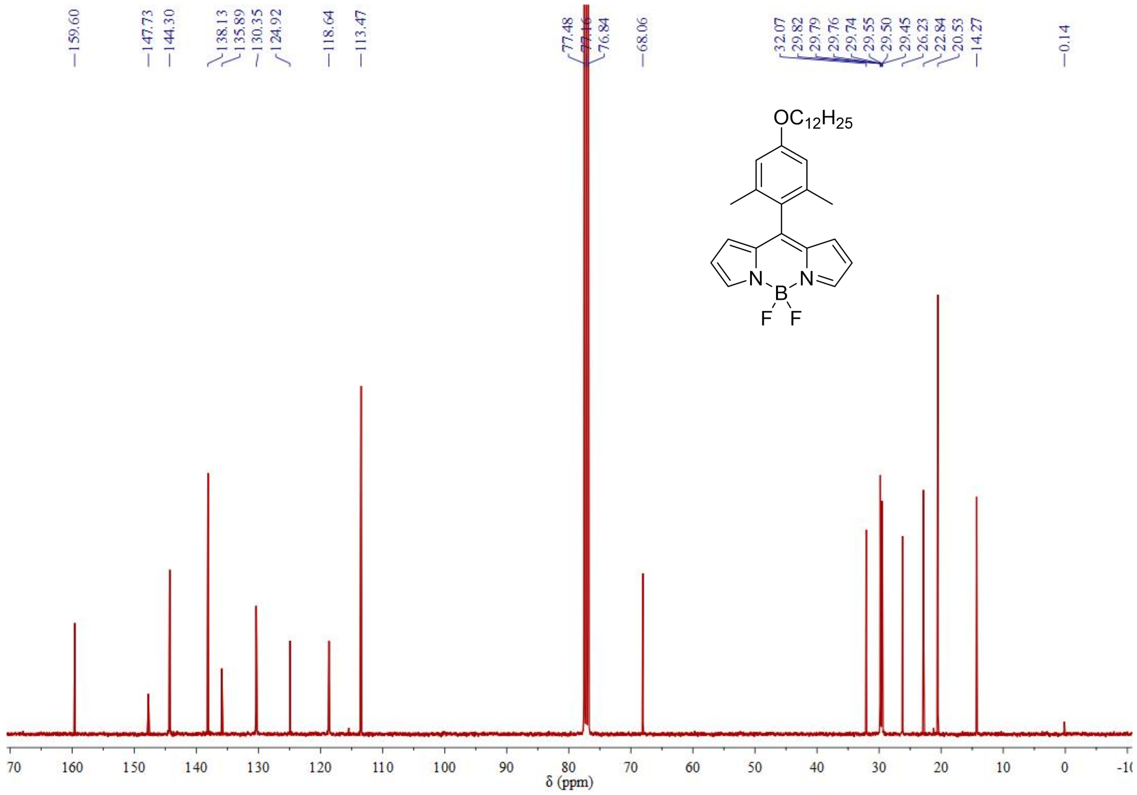

^{1}H NMR (400 MHz, $CDCl_3$) for **1Br**

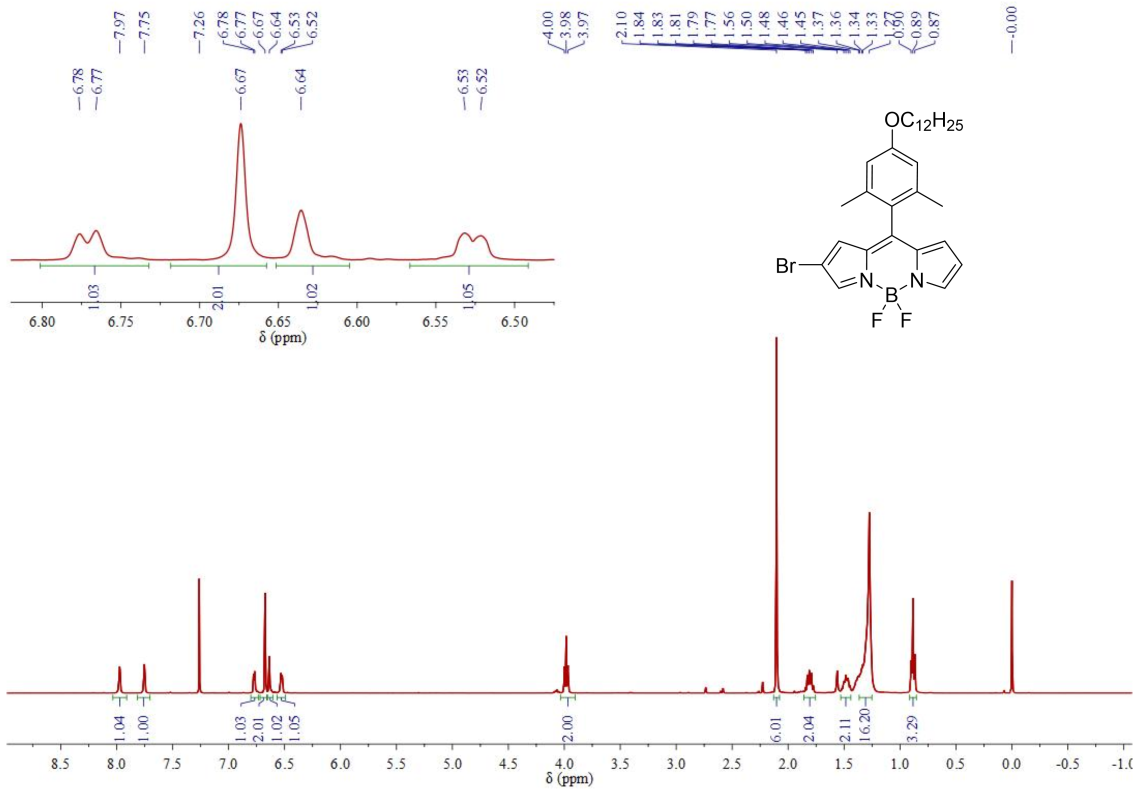

^{13}C NMR (100 MHz, $CDCl_3$) for **1Br**

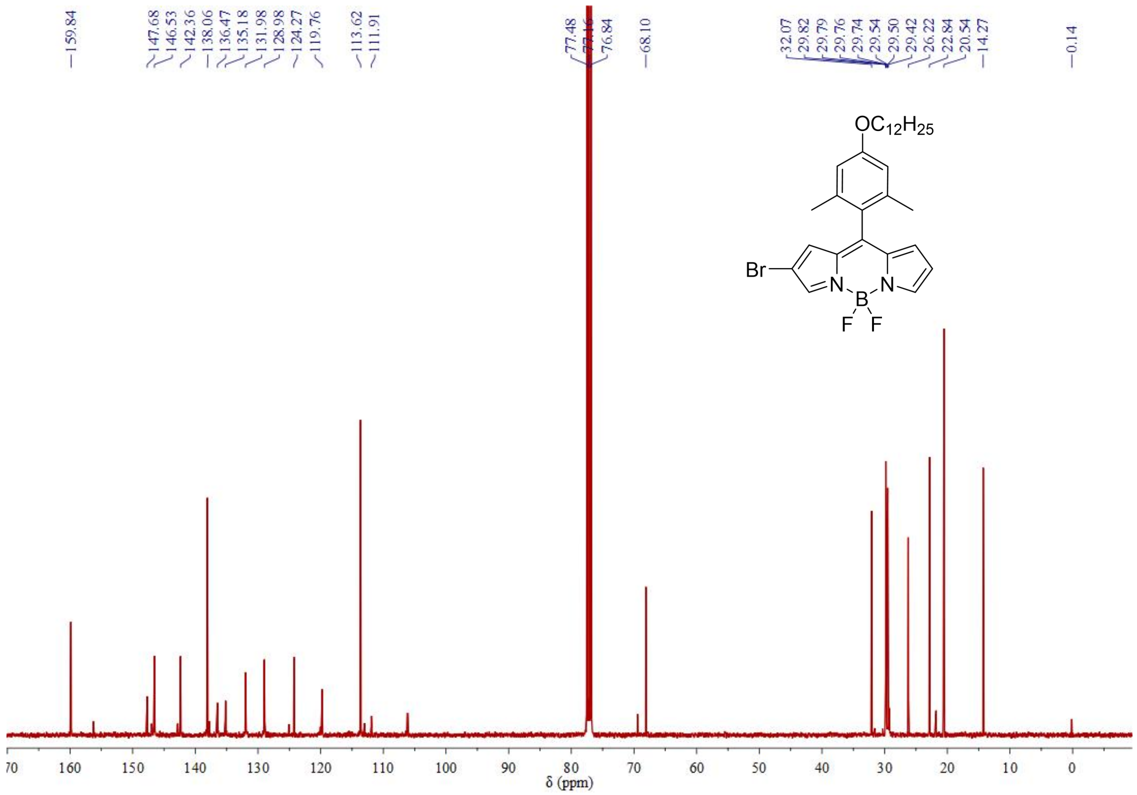

^1H NMR (400 MHz, $CDCl_3$) for **2**

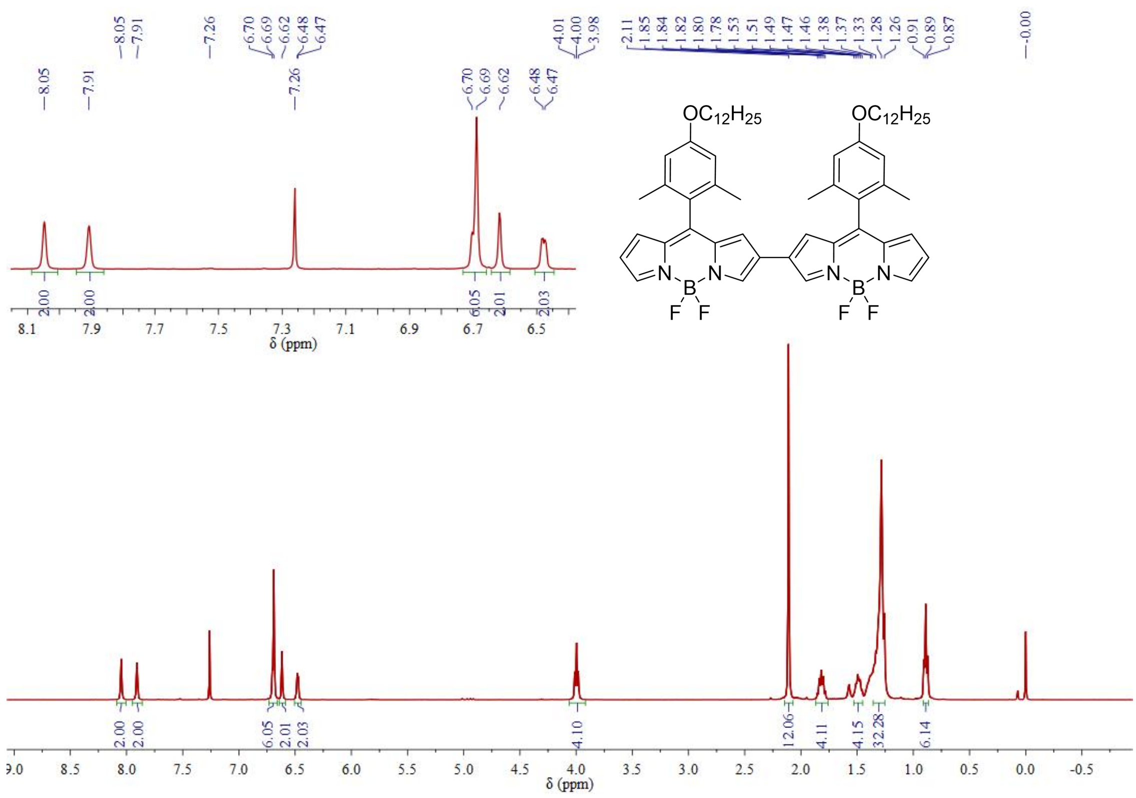

^{13}C NMR (100 MHz, $CDCl_3$) for **2**

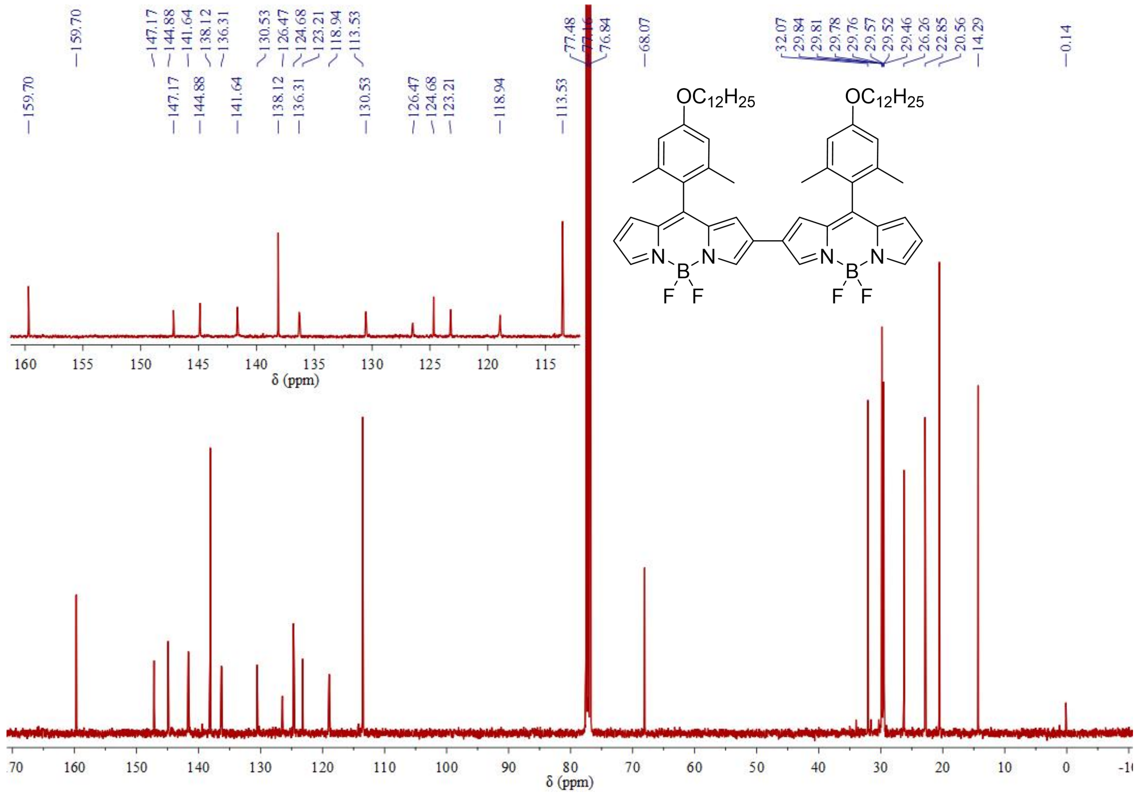

^{1}H NMR (400 MHz, $CDCl_3$) for **2Br**

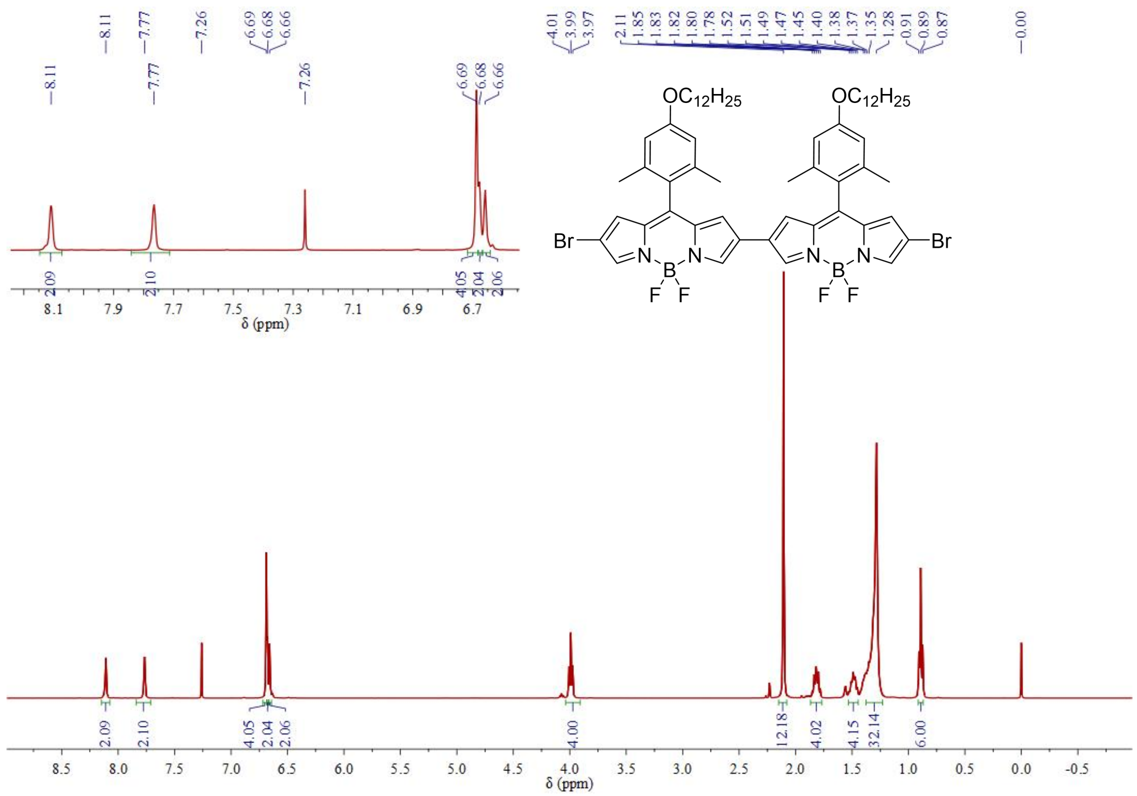

^{13}C NMR (100 MHz, $CDCl_3$) for **2Br**

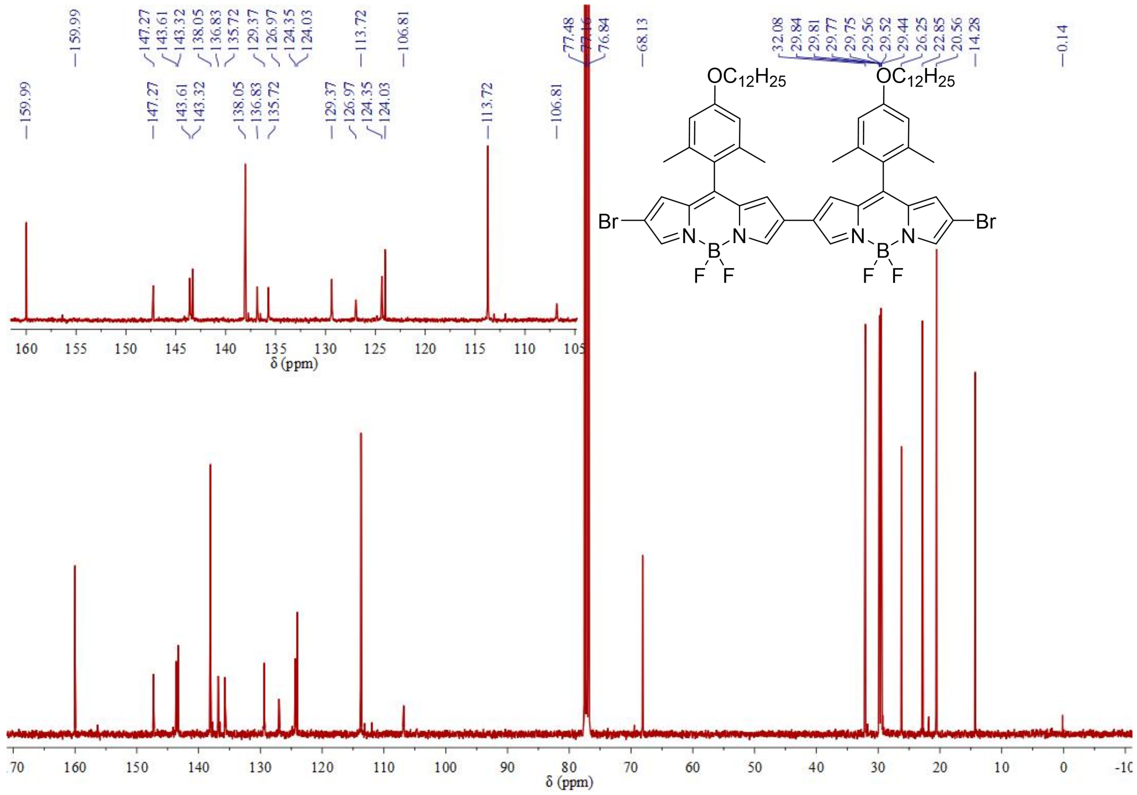

^{1}H NMR (400 MHz, $CDCl_3$) for **3**

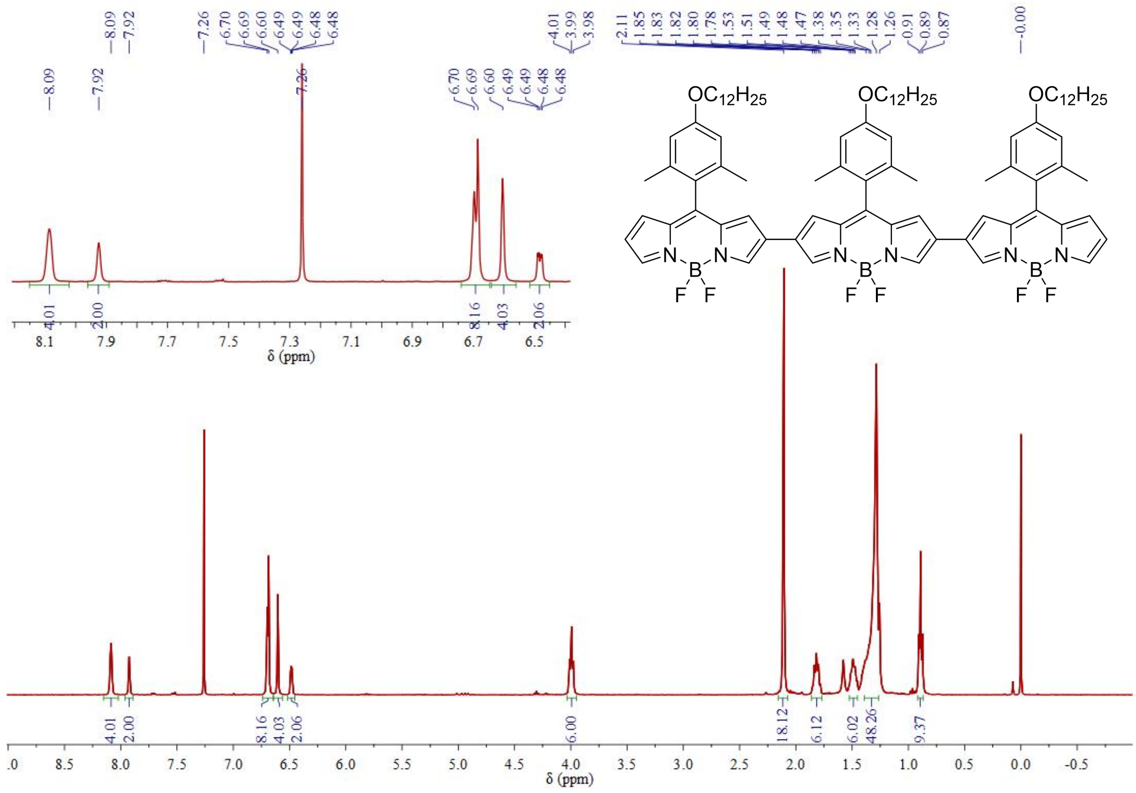

^{13}C NMR (100 MHz, $CDCl_3$) for **3**

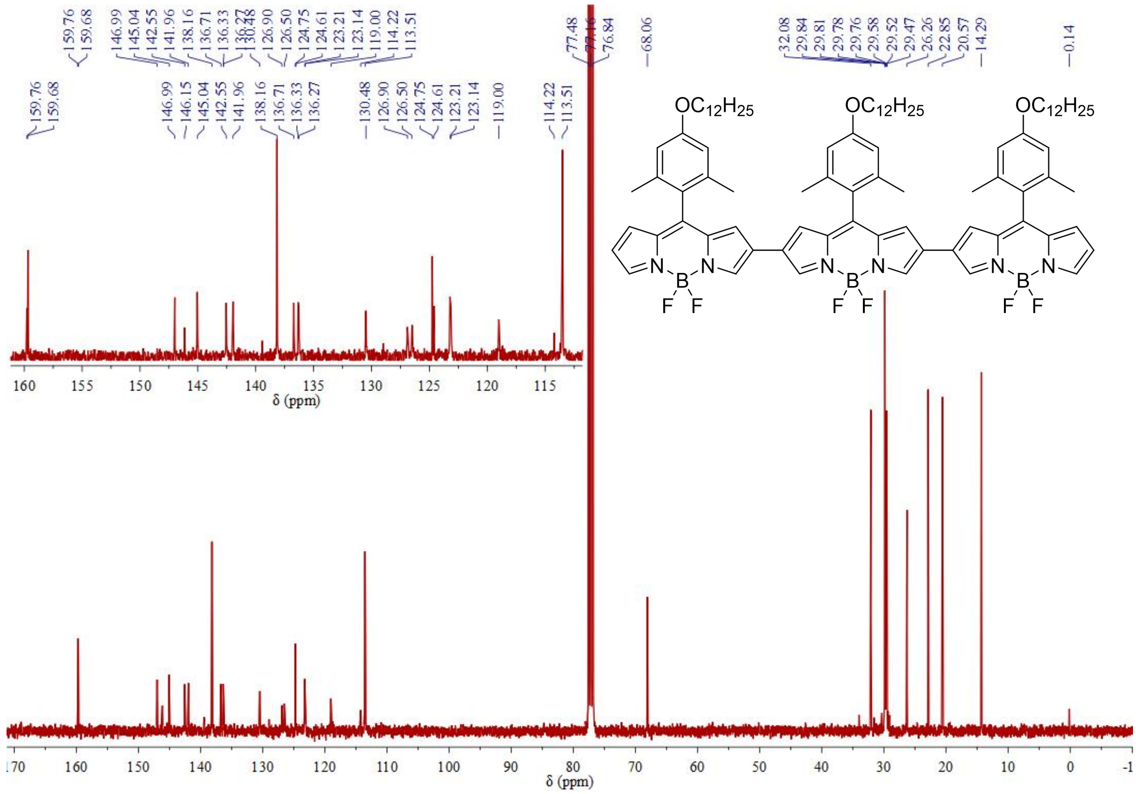

^{1}H NMR (400 MHz, $CDCl_3$) for **4**

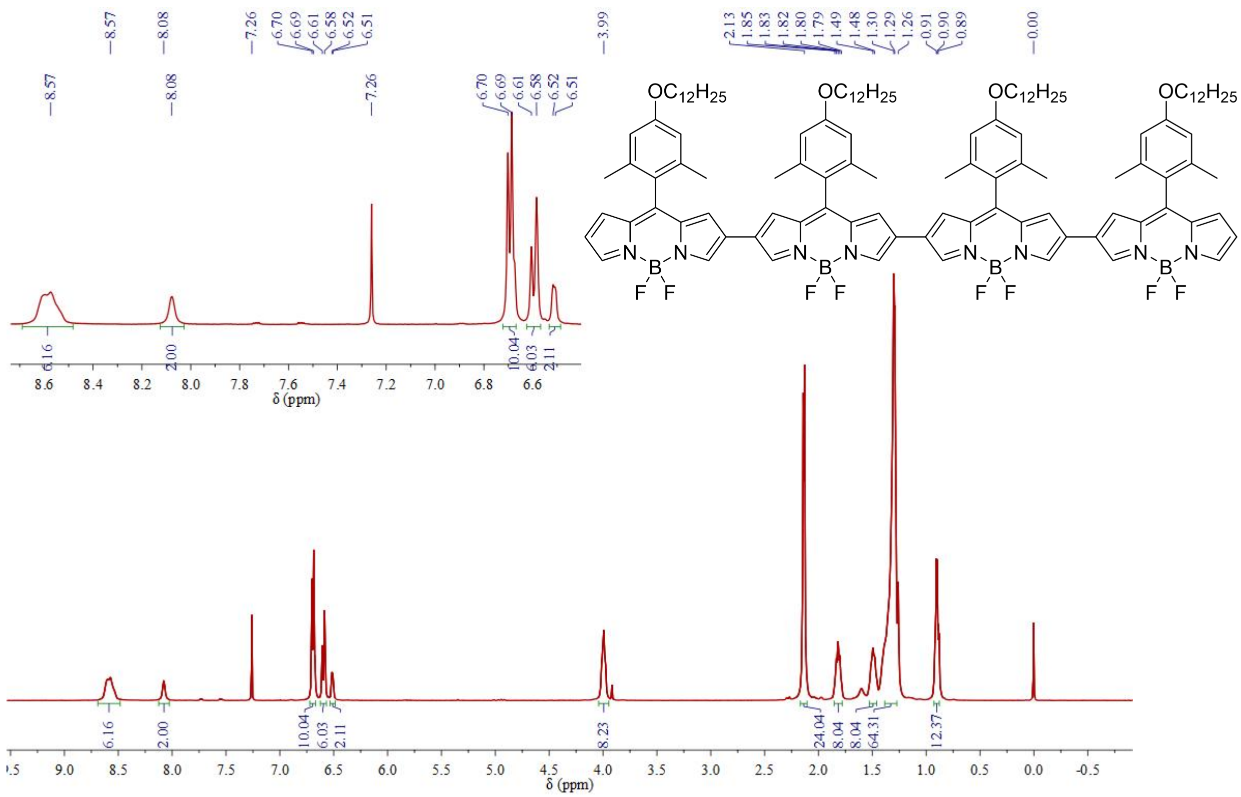

^{13}C NMR (100 MHz, $CDCl_3$) for **4**

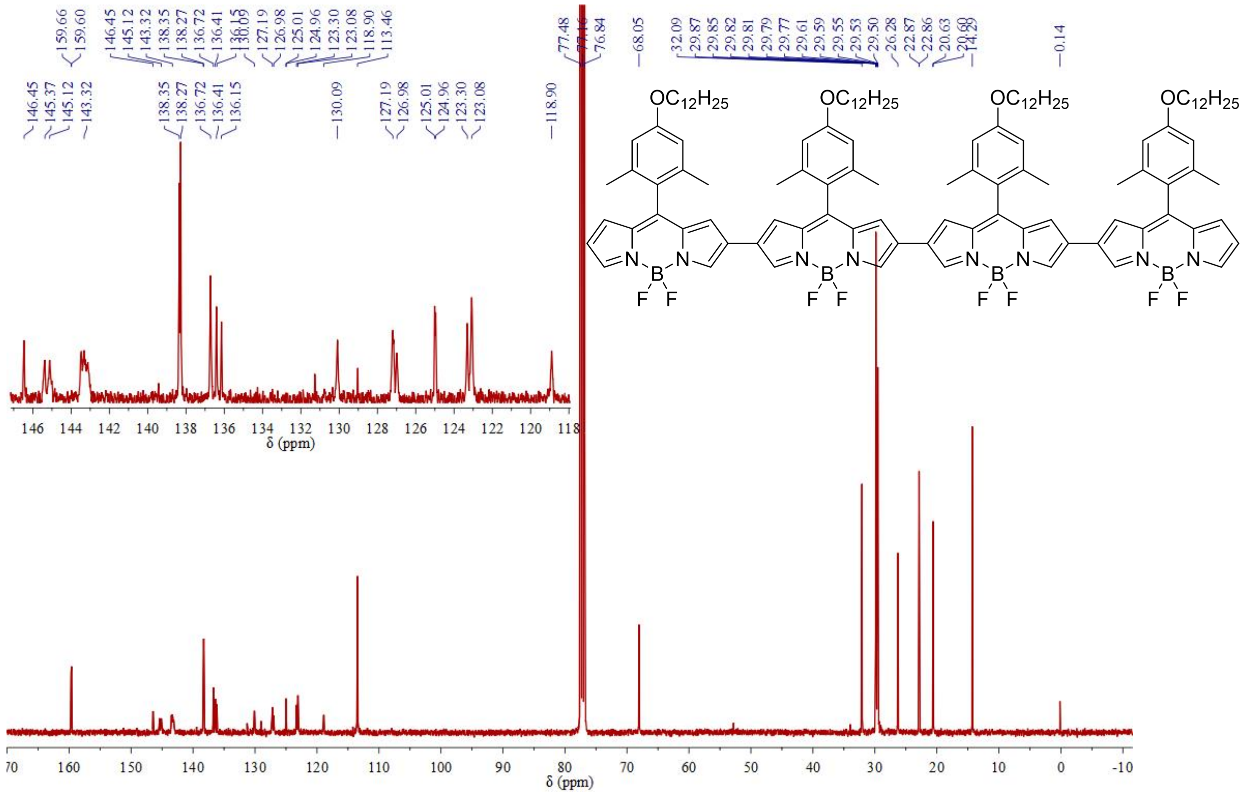

9. High resolution mass spectra for all new compounds

HRMS for **BDP-OH**

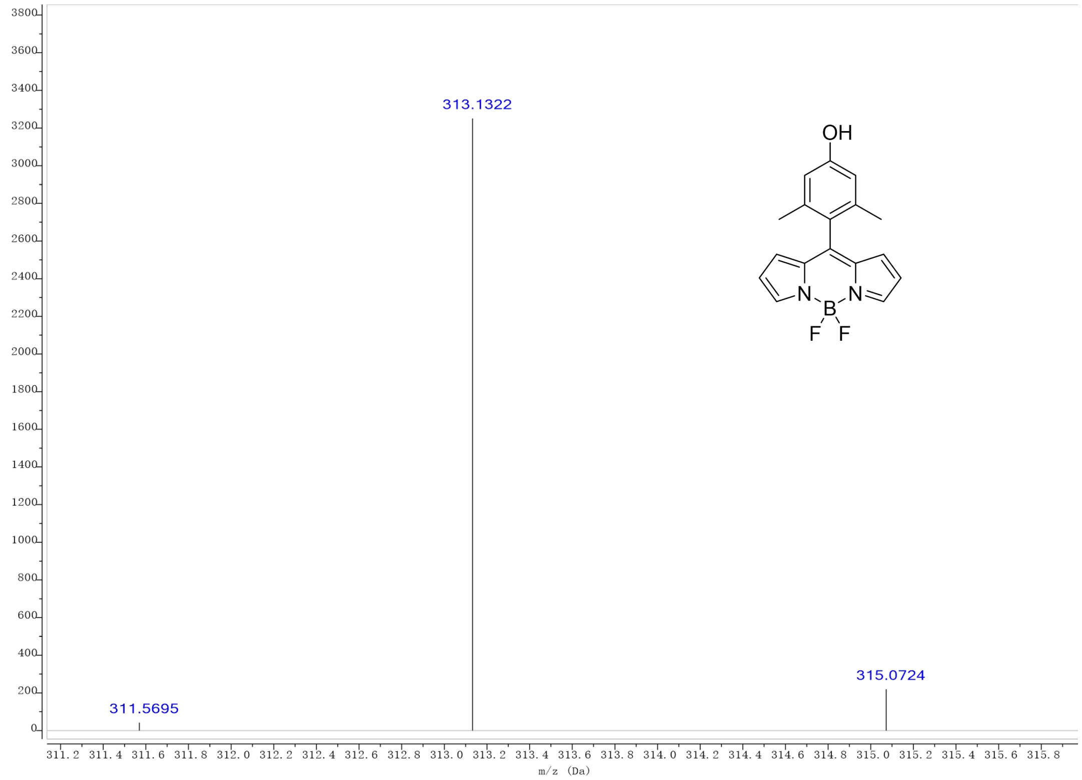

HRMS for **1**

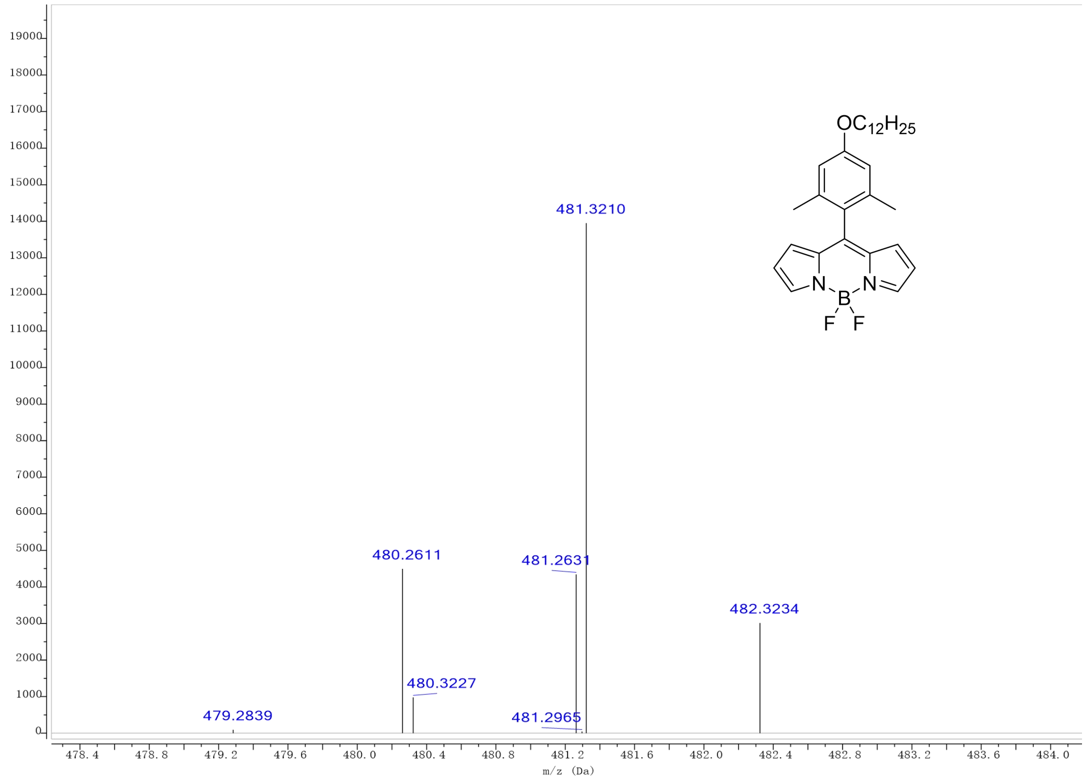

HRMS for **1Br**

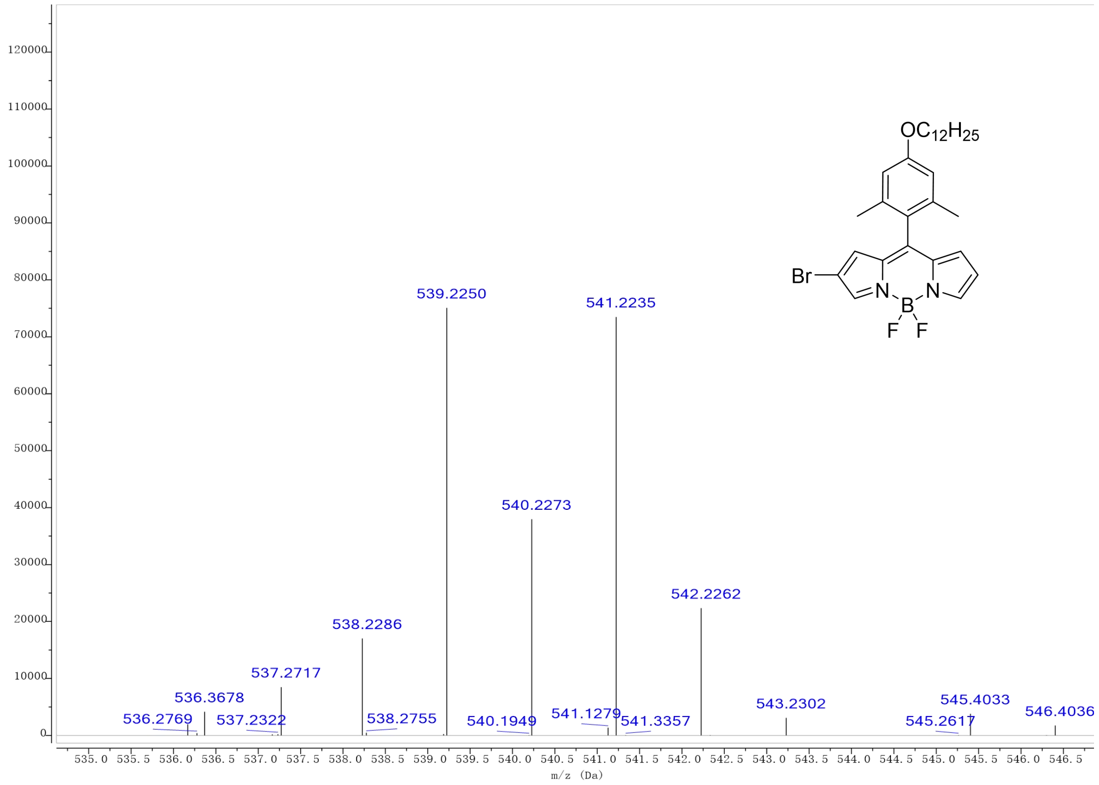

HRMS for **2**

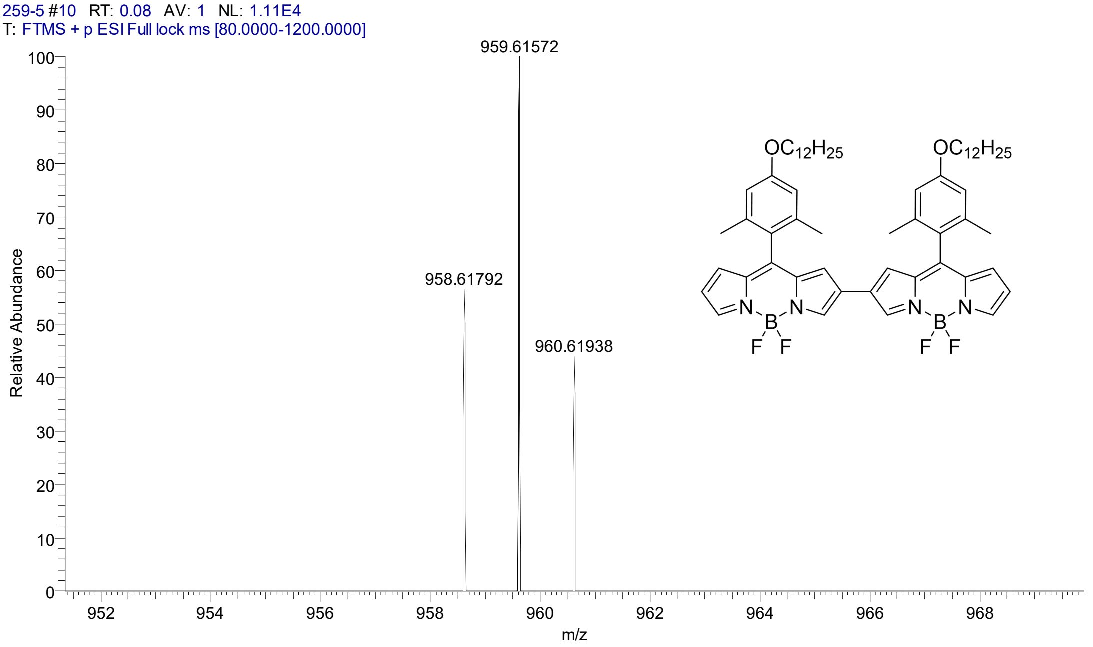

HRMS for **2Br**

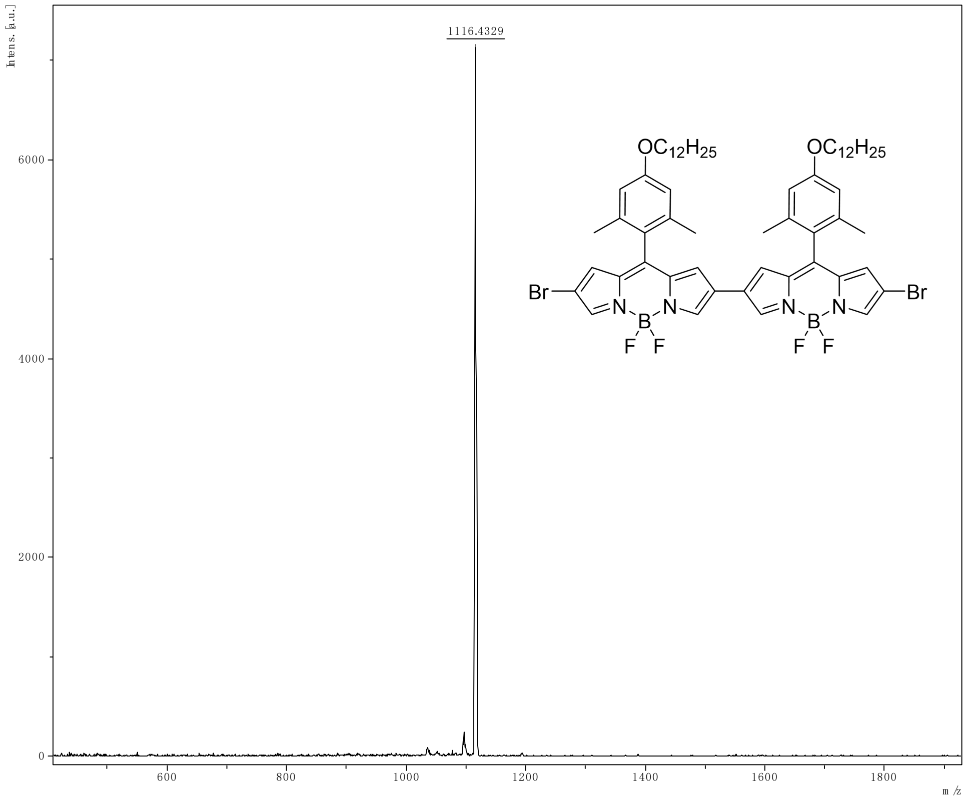

HRMS for **3**

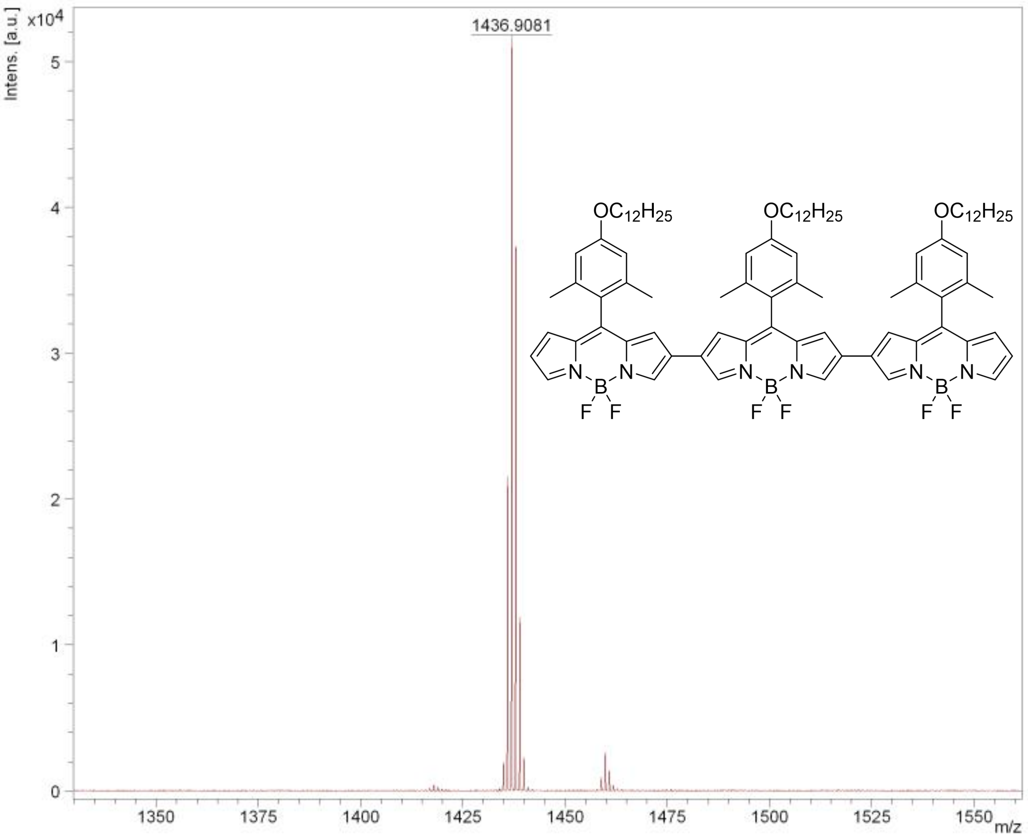

HRMS for **4**

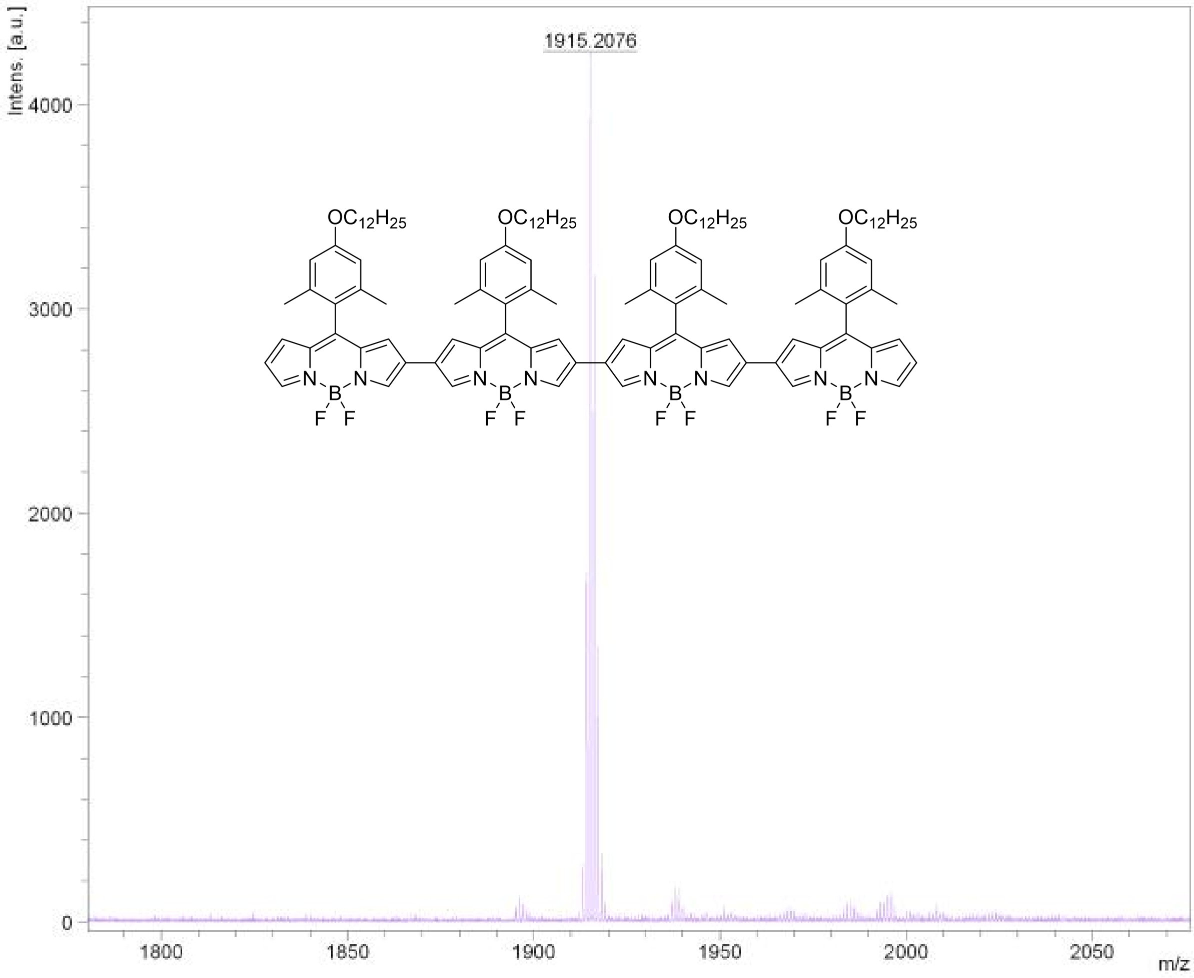

Intens. [a.u.]
1915.2076
4000
3000
2000
1000
0
1800
1850
1900
1950
2000
2050
m/z
OC12H25
N
B
F
F

10. Reference

1. Y. Kawamura, H. Sasabe and C. Adachi, *Jpn. J. Appl. Phys*. 2004, **43**, 7729–7730.
2. (a) Z. Feng, L. Jiao, Y. Feng, C. Yu, N. Chen, Y. Wei, X. Mu and E. Hao, *J. Org. Chem*. 2016, **81**, 6281–6291. (b) W. Li, L. Li, H. Xiao, R. Qi, Y. Huang, Z. Xie, X. Jing and H. Zhang, *RSC Adv*. 2013, **3**, 13417–13421.
3. S. Mishra, S. B. Shelar, S. Rout, P. A. Hassan, K. C. Barick and N. Agarwal, *ACS Appl. Bio. Mater*. 2024, **7**, 7207–7218.
4. (a) B. S. Lane, M. A. Brown and D. Sames, *J. Am. Chem. Soc.* 2005, **127**, 8050–8057. (b) Y. Li, W. Wang, S. Yang, B. Li, C. Feng and Z. Shi, *Chem. Commun.* 2010, **46**, 4553–4555. (c) Z. Liang, J. Zhao and Y. Zhang, *J. Org. Chem.* 2010, **75**, 170–177.
5. (a) L. J. Patalag, L. P. Ho, P. G. Jones and D. B. Werz, *J. Am. Chem. Soc.* 2017, **139**, 15104−15113. (b) S. F. Völker, A. Schmiedel, M. Holzapfel, K. Renziehausen, V. Engel and C. Lambert, *J. Phys. Chem. C* 2014, **118**, 17467-17482.
6. (a) C. Schäfer, R. Ringström, J. Hanrieder, M. Rahm, B. Albinsson and K. Börjesson, *Nat. Commun.* 2024, **15**, 8705. (b) Y. Hou, Q. Liu and J. Zhao, *Chem. Commun.* 2020, **56**, 1721–1724.
7. Y. Hayashi, S. Yamaguchi, W. Y. Cha, D. Kim and H. Shinokubo, *Org. Lett.* 2011, **13**, 2992–2995.
8. M. J. Frisch, G. W. Trucks, H. B. Schlegel, G. E. Scuseria, M. A. Robb, J. R. Cheeseman, G. Scalmani, V. Barone, B. Mennucci, G. A. Petersson, H. Nakatsuji, M. Caricato, X. Li, H. P. Hratchian, A. F. Izmaylov, J. Bloino, G. Zheng, J. L. Sonnenberg, M. Hada, M. Ehara, K. Toyota, R. Fukuda, J. Hasegawa, M. Ishida, T. Nakajima, Y. Honda, O. Kitao, H. Nakai, T. Vreven, J. A. Montgomery, J. E. Peralta, F. Ogliaro, M. Bearpark, J. J. Heyd, E. Brothers, K. N. Kudin, V. N. Staroverov, T. Keith, R. Kobayashi, J. Normand, K. Raghavachari, A. Rendell, J. C. Burant, S. S. Iyengar, J. Tomasi, M. Cossi, N. Rega, J. M. Millam, M. Klene, J. E. Knox, J. B. Cross, V. Bakken, C. Adamo, J. Jaramillo, R. Gomperts, R. E. Stratmann, O. Yazyev, A. J. Austin, R. Cammi, C. Pomelli, J. W. Ochterski, R. L. Martin, K. Morokuma, V. G. Zakrzewski, G. A. Voth, P. Salvador, J. J. Dannenberg, S. Dapprich, A. D. Daniels, O. Farkas, J. B. Foresman, J. V. Ortiz, J. Cioslowski and D. J. Fox, Gaussian 09, Revision D.01; Gaussian, Inc.: Wallingford, CT, 2013.
9. X. Gao, S. Bai, D. Fazzi, T. Niehaus, M. Barbatti and W. Thiel, *J. Chem. Theory Comput.* 2017, **13**, 515–524.
